# G-quadruplexes are transcription factor binding hubs in human chromatin

**DOI:** 10.1186/s13059-021-02324-z

**Published:** 2021-04-23

**Authors:** Jochen Spiegel, Sergio Martínez Cuesta, Santosh Adhikari, Robert Hänsel-Hertsch, David Tannahill, Shankar Balasubramanian

**Affiliations:** 1 Cancer Research UK Cambridge Institute, Li Ka Shing Centre, Robinson Way, Cambridge, CB2 0RE UK; 2 Department of Chemistry, University of Cambridge, Cambridge, CB2 1EW UK; 3 Present Address: Data Sciences and Quantitative Biology, Discovery Sciences, AstraZeneca, Cambridge, UK; 4 Present Address: Center for Molecular Medicine Cologne, University of Cologne, 50931 Cologne, Germany; 5 School of Clinical Medicine, University of Cambridge, Cambridge, CB2 0SP UK

**Keywords:** Transcription factor binding, DNA G-quadruplex, Gene expression, Chemical biology

## Abstract

**Background:**

The binding of transcription factors (TF) to genomic targets is critical in the regulation of gene expression. Short, double-stranded DNA sequence motifs are routinely implicated in TF recruitment, but many questions remain on how binding site specificity is governed.

**Results:**

Herein, we reveal a previously unappreciated role for DNA secondary structures as key features for TF recruitment. In a systematic, genome-wide study, we discover that endogenous G-quadruplex secondary structures (G4s) are prevalent TF binding sites in human chromatin. Certain TFs bind G4s with affinities comparable to double-stranded DNA targets. We demonstrate that, in a chromatin context, this binding interaction is competed out with a small molecule. Notably, endogenous G4s are prominent binding sites for a large number of TFs, particularly at promoters of highly expressed genes.

**Conclusions:**

Our results reveal a novel non-canonical mechanism for TF binding whereby G4s operate as common binding hubs for many different TFs to promote increased transcription.

## Introduction

Transcription factors (TFs) control gene expression and chromatin structure through precise protein-DNA interactions at specific genome locations [1]. Preferred binding sites for hundreds of TFs exhibit short, defined DNA recognition motifs, commonly called “consensus sequences,” based on in vitro binding studies [2–4] and also in chromatin using ChIP-seq [5]. Two modes of protein-DNA recognition are described to contribute to TF binding specificity [6]. The first, based on the nucleotide readout, involves hydrogen bonding and hydrophobic interactions between amino acid side chains of the TF with base pairs primarily in the major groove of the DNA helix [7]. The second mode uses shape readout and is mediated by local structural features of the DNA double helix, such as minor groove width, base roll, and helix twist [8–10]. TF binding specificity can also be influenced by co-binding proteins [4] as well as epigenetic features such as CpG-methylation [11] and nucleosome positioning [12]. Despite this progress, experimentally observed binding sites for many TFs have not been explained [13]. As it is an open question as to what possible genomic features determine such binding events, we set out to explore how alternative DNA secondary structures, called G-quadruplexes, contribute to TF binding.

DNA G-Quadruplexes (G4s) are secondary structures made up of stacked G-tetrads, with each tetrad formed from the co-planar arrangement of four Hoogsteen-bonded guanine bases (Additional file 1: Fig. S1a) [14]. G4 structures have been visualized in human cells [15] and mapped in chromatin to regulatory regions particularly in promoters of highly expressed cancer genes [16, 17]. Analysis of patient-derived breast cancer tumor xenograft models has recently revealed a relationship of G4s with somatic copy-number aberrations and underlying transcriptional programs [18]. This together with small molecule perturbation experiments [19] is suggestive of important roles for G4s in transcriptional regulation. Biophysical and biochemical affinity experiments have identified proteins, such as helicases and DNA binding proteins, that show selective recognition for G4s over double-stranded DNA in vitro [20, 21]*.* The detailed molecular and functional relationship between endogenous G4s and components of the transcription machinery therefore warrants thorough investigation.

Herein, we report that numerous TFs are recruited to sites of endogenous G4s in human chromatin. Supporting this, the binding of several TFs to G4 structures is shown to have affinities comparable to that of canonical DNA double-strand interactions. Promoter G4s also appear to be bound by a surprisingly large number of TFs, particularly for highly expressed genes. Moreover, within a chromatin context, we provide robust evidence to demonstrate that TF binding to G4s can be competed out with a G4-selective small molecule. We posit that G4s are a previously overlooked key element of gene regulation that serves as high-affinity hubs enabling the recruitment of many different TFs to the same site to promote active transcription.

## Results

### TF binding is tightly linked to endogenous G4 structures in the human genome

**Fig. 1:**
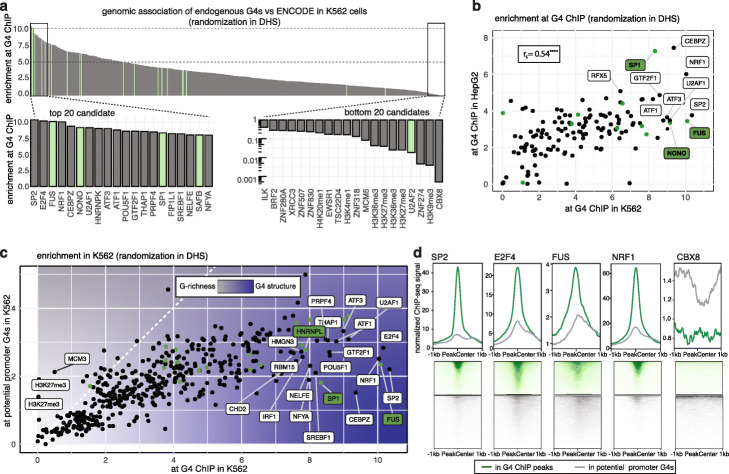
TF binding is tightly linked to endogenous G4 structures in the human genome. **a** Enrichment of 524 ENCODE proteins at G4 ChIP-seq sites in K562 for randomization in open chromatin (DHS). Top 20 and bottom 20 candidates are highlighted. Green shading indicates proteins with reported G4-association. **b** Genomic association with endogenous G4s is consistent in K562 and HepG2 cells. Spearman correlation test (*r*_s_, ****P < 0.0001) is based on the maximum enrichment observed for TFs that have been mapped in both K562 (*x*-axis) and HepG2 (*y*-axis) cells. Green shading indicates proteins with reported G4-association. **c** Genomic association of TFs obtained from ENCODE with endogenous G4s (*x*-axis) and potential G4 control sites at promoters (*y*-axis). Proteins for which binding is independent of secondary structure formation should show similar enrichment for both data sets (white dashed line). Green shading indicates proteins with reported G4 association. **d** Occupancy profiles of enriched candidates SP2, E2F4, FUS, and NRF1 and a non-enriched TF, CBX8, around endogenous G4 sites (green) and control sequences (gray). The strandedness of endogenous G4s was derived from stranded data of sequences with G4 forming potential [26] (see “Materials and methods”)

As DNA structure is fundamental to DNA-protein interactions, we explored the relationship of endogenous TF binding and G4 secondary structures. For this, we used human K562 chronic myelogenous leukemia cells and HepG2 hepatocellular carcinoma cells, as these have been extensively mapped for protein binding sites by ENCODE [22]. We first generated genome-wide maps of G4 structures (Additional file 1: Fig. S1b, hereafter referred to as endogenous G4s) from chromatin of K562 and HepG2 cells by G4 ChIP-seq [23] using the G4 structure-specific antibody BG4 [24]. To eliminate possible antibody interactions with chromatin-associated RNA or DNA/RNA hybrid G4s, chromatin was treated with RNase A prior to immunoprecipitation [15, 25]. We observed thousands of endogenous G4 sites in both K562 (9205 sites) and HepG2 (8805 sites) with 4825 sites in common between the cell lines (Additional file 1: Fig. S1c-e). Most endogenous G4s (8688/9205, 94% in K562; 6894/8805, 78% in HepG2) encompassed sequences previously shown to physically form G4 structures by an in vitro genome-wide DNA Polymerase stop-assay [26] (hereafter called potential G4s) (Additional file 1: Fig. S1f and g). The majority of endogenous G4s (9043/9205, 98% in K562; 8430/8805, 96% in HepG2) were located in open chromatin, as defined by overlap with DNase hypersensitivity sites. In both cell lines, many of these G4s (~ 40%) were found in promoters ~ 80 bp upstream of transcription start sites (TSS) (Additional file 1: Fig. S1h and i). We then compared endogenous G4s to the binding sites of various chromatin-associated proteins and histone marks derived from ENCODE (for a full list see Additional files 2 and 3: Supplemental Data Table S1 and S2). This analysis showed that many TFs were enriched at endogenous G4 sites and is immediately suggestive of direct TF-G4 interactions (Fig. 1a and Additional file 1: Fig. S2), particularly since several of the most enriched proteins, such as FUS and SP1, have previously been suggested to interact with DNA G4s in vitro [20]. Despite each cell line having a distinct G4 landscape, TFs mostly displayed a similar enrichment at endogenous G4s (Spearman correlation *r*_s_ = 0.54, see Fig. 1b and Additional file 4: Supplemental Data Table S3), suggesting that G4 binding is a general property of certain TFs. Endogenous G4s were substantially devoid of both transcriptional repressors (e.g., CBX8, ZNF318, EZH2 and PHB2) and repressive histone marks (e.g., H3K27me3, H3K9me3) (Fig. 1a and Additional file 1: Fig. S2) which is consistent with previous observations that endogenous promoter G4s are linked to high transcription levels [16, 17].

To confirm that the observed TF enrichment at G4s is not due to G-richness of primary sequences, but is dictated by secondary structure (Additional file 1: Fig. S3a), we evaluated control sites that have G4-forming potential [26] at promoters (1 kb upstream TSS as well as 5’UTR) of open chromatin, but have no detectable endogenous G4 structure (Additional file 1: Fig. S3b and c). Many TFs were found to display greater enrichment at endogenous G4s than at G-rich control sites (Fig. 1c and Additional file 5: Supplemental Data Table S4). For example, at endogenous G4s, SP2 is enriched 10.3-fold compared to 2.2-fold at G-rich control sites, which suggests that G4 secondary structure is important for particular TFs. The average TF ChIP-seq binding signal for the strongest enriched TFs was also much higher at endogenous G4s, compared to control sites (Fig. 1d). The average TF ChIP-seq signal for the strongest enriched TFs was also much higher at endogenous G4s, compared to control sites (Fig. 1d). Consistent with direct recruitment of TFs to G4 structures, the occupancy profile was generally centered around endogenous G4 sites for a large number of TFs (e.g., 100 TFs were within ± 20 bp and 177 within ± 40 bp) (Additional file 1: Fig. S2e).

R-loops (three-stranded DNA–RNA hybrids) form when nascent RNA anneals back to template DNA. R-loops have been associated with GC-rich promoter regions [27], while the interplay of G4s and R-loops has been suggested to influence transcription [28]. Using R-ChIP and DRIP-seq data for K562 cells [29], some co-occurrence of endogenous G4s and R-loop was observed (1431 overlapping peaks). R-loops were located mostly on the opposite strand and downstream (~ 140 bp) of the G4s (Additional file 1: Fig. S4a). While there are several TFs that appear to be enriched downstream of endogenous G4s indicating interactions with R-loops, the majority of TFs is centered on G4s (Additional file 1: Fig. S2e). Importantly, ChIP signal profiles for TF highly enriched at G4s, such as SP2, E2F4, NRF1, or FUS, were found to be centered on the G4s rather than R-loops supporting a direct recruitment to G4s rather than R-loops (Fig. S4b).

We next investigated the relative contributions of G4s and double-stranded DNA to TF recruitment, by comparing TF enrichment at endogenous G4s vs consensus binding sites obtained from JASPAR [30]. Most TFs (165/193, ~ 85%) showed equal or greater enrichment at endogenous G4s in K562 cells compared to consensus promoter binding sites (Additional file 1: Fig. S5a and Additional file 6: Supplemental Data Table S5) and 32 TFs (including SP2, SP1 and E2F4) displayed more than 2-fold stronger enrichment at endogenous G4s than at predicted consensus promoter binding sites in open chromatin (Additional file 1: Fig. S5b and Additional file 6: Supplemental Data Table S5). These data suggest that G4 secondary structures can recruit several TFs more effectively than double-stranded DNA.

Five of the twenty proteins most enriched at G4s in K562 cells (FUS, NONO, U2AF1, HNRNPK, and HNRNPL) are classified as recognizing RNA or single-stranded DNA (Additional file 1: Fig. S5c) and often not considered as conventional TFs, as they lack specific double-stranded DNA binding sequences [1]. These proteins are clearly important in transcriptional regulation [31], but it is not known whether they bind DNA directly. Our findings support that these factors are tightly associated with G4s and that some of these proteins can be recruited to chromatin via DNA G4 structures.

### TFs selectively bind G4 structures

**Fig. 2:**
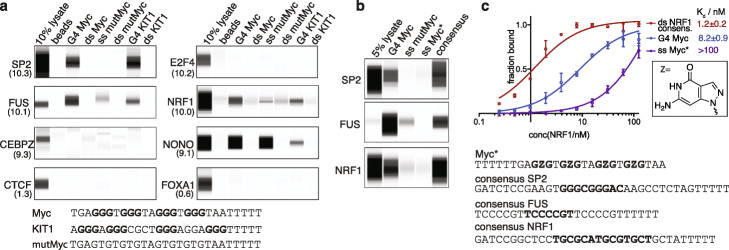
TFs selectively bind to G4 structures. **a** Affinity pull-down western blot analysis of different G4 oligonucleotides and control sequences. Genomic enrichment at endogenous G4s in K562 for randomization in open chromatin is shown in brackets. **b** Affinity pull-down of SP2, FUS, and NRF1 using a G4 oligomer, single-stranded oligomers unable to form a G4 structure (ss mutMyc and ss Myc*) and respective consensus sequences. **c** Binding curves as determined by ELISA show high-affinity binding of recombinant FLAG-NRF1 to a NRF1 double-stranded DNA consensus sequence and G4 structures, but significantly weaker binding to a single-stranded 7-deaza control (error bars display standard deviation, *N* = 3)

To confirm that the endogenous G4-enriched TFs identified above bind directly to DNA G4s, we carried out biophysical interaction assays. Single-stranded, 3′-biotinylated oligonucleotides that fold into well-characterized (Additional file 1: Table S1) G4 structures (G4 Myc and G4 Kit1) were deployed alongside double-stranded DNA control oligonucleotides and mutated or 8-aza-7-deazaguanosine-substituted [32] (ssMyc*) controls that cannot fold into G4s, with the ssMyc* control maintaining the same G-richness of the parent sequence. The presence or absence of G4 formation was confirmed via circular dichroism spectroscopy (Additional file 1: Fig. S6). G4-binding TFs were affinity captured from K562 nuclear extracts using immobilized oligonucleotides followed by western blotting analysis using specific antibodies. Based on their enrichment at endogenous G4s in K562 and HepG2 cells, we selected 33 highly enriched TFs to investigate their G4 binding properties. Strikingly, a large fraction of TFs (22/33, 66%) showed capacity to bind to G4 structures (Fig. 2a, Additional file 1: Fig. S7 and Table S2). Most of the candidates bound to both Myc G4 and Kit1 G4, while few TFs (e.g., SRSF1, RBM15) had a preference for one G4 structure. Crucially, there was little or no binding to mutant single-stranded and double-stranded controls for the majority of G4-binding TFs (17/22). Furthermore, little or no binding was seen with a single-stranded 7-deazaguanine control sequence (ssMyc*) for the top enriched candidates (SP2, FUS, and NRF1; Fig. 2b), which further confirms that G4 structure formation alone and not G-richness is required for binding. In contrast, four candidates (e.g., NONO) were more promiscuous and bound G4s to a similar extent to that of at least one of the control sequences, while TARDBP showed a very strong preference for single-strand DNA. No detectable G4 binding was seen for some highly enriched TFs, such as E2F4 and CEBPZ, so these proteins may be recruited to G4s via other indirect interactions. Alternatively, structural features co-incident with endogenous G4s, such as i-motifs [33, 34] or R-loops [35], possibly contribute to their recruitment. The enrichment level of SP2, NRF1, FUS, MYC, YY1, and ZHX1 was comparable to their binding to consensus sequence controls (Fig. 2b and Additional file 1: S7b), which is mostly in line with previous reports [36–38]. Importantly, two negative controls, FOXA1 and CTCF (Fig. 2a), that show low enrichment at endogenous G4s (Additional file 2: Supplemental Data Table S1), did not bind to the G4 oligonucleotides, with CTCF also serving as a control due to its G-rich consensus binding motif. Notably, affinity enrichment experiments from nuclear lysate cannot distinguish direct G4 binding from co-binding events; however, our findings are consistent with the recruitment of numerous TFs to G4 structures in chromatin (Additional file 1: Fig. S8).

To measure the apparent binding affinities of TF-G4 interactions, we employed an enzyme-linked immunosorbent assay (ELISA) with NRF1 as an exemplar, since it was highly enriched at endogenous G4s in both K562 and HepG2 chromatin, but notably, does not have a G-rich double-stranded DNA consensus motif (Additional file 1: Fig. S3). Recombinant NRF1 displayed strong binding to a double-stranded DNA consensus sequence (*K*_d_ = 1.2 ± 0.2 nM) and folded G4 Myc structure (*K*_d_ = 8.2 ± 0.9 nM), but considerably weaker binding to single-stranded 8-aza-7-deazaguanosine-substituted ssMyc* (*K*_d_ > 100 nM) (Fig. 2c). We also observed nanomolar affinity for four other defined G4 structures tested (*K*_d_ ranging from 1.9–7.5 nM) and 4–14 fold selectivity over their corresponding double-stranded control sequences, highlighting the importance of G4 secondary structure formation for binding at these sites (Additional file 1: Fig. S9).

### Competition of TF binding to G4s in native chromatin by small molecule ligands

**Fig. 3:**
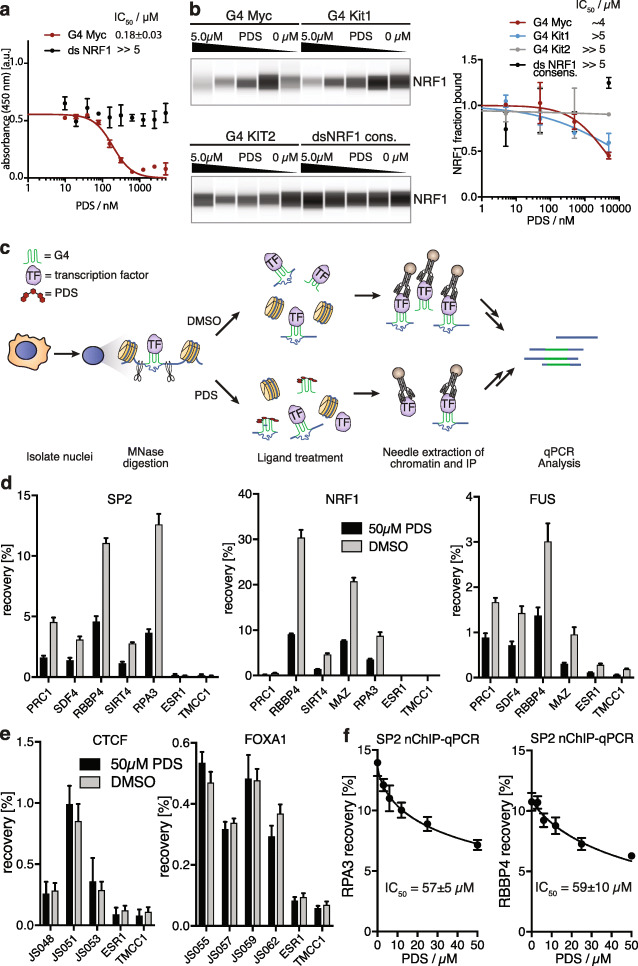
Competition of TF binding to G4s in native chromatin by small molecule ligands. **a** Competition ELISA. Immobilized G4 Myc and a double-stranded DNA consensus oligomer were pre-incubated with increasing concentrations of G4 ligand PDS followed by recombinant FLAG-NRF1 (20 nM) (error bars display standard deviation, *N* = 3). **b** PDS dose-dependent competition for NRF1 in K562 cell nuclear lysates. PDS displaces TFs from different G4 oligomers, but does not interfere with binding to the double-stranded DNA consensus oligomer (error bars display standard deviation, *N* = 2). **c** Scheme for TF displacement upon G4 ligand treatment and detection via native ChIP. **d** Native ChIP-qPCR for G4-associated SP2, NRF1, and FUS binding shows a PDS-dependent signal reduction. *x*-axis, selected positive regions for G4 ChIP-seq and TF ENCODE ChIP signal and two negative control regions (ESR1, TMCC1) with no G4 and TF ChIP-seq signal (error bars display standard error of the mean, *N* = 4). **e** Native ChIP-qPCR of control CTCF and FOXA1 are not displaced by PDS (error bars display standard error of the mean, *N* = 4). **f** PDS-dependent signal reduction in native SP2 ChIP-qPCR at two positive regions (error bars display standard error of the mean, *N* = 3)

Chromatin architecture affects both TF recruitment and the G4 landscape [16]; therefore, it is essential to validate and study TF-G4 interactions in a native chromatin context. Genome-editing of G4-forming sequences in promoters would unavoidably change TF binding site sequences in double-stranded DNA, so we employed a G4-specific small molecule to selectively compete with TFs at endogenous G4 sites. We assessed the small molecule pyridostatin (PDS) [39] for selective competition using ELISAs. PDS competed with human NRF1 binding to Myc G4 DNA with an *IC*_50_ value of 0.18 ± 0.03 μM, which is in agreement with the previously determined G4 binding affinity for PDS [40] (Fig. 3a). In contrast, PDS did not impair NRF1 binding to its double-stranded DNA consensus sequence (Fig. 3a). Similarly, affinity enrichment experiments for SP2, NRF1, and FUS from K562 nuclear lysates showed that PDS could inhibit binding to folded G4 oligomers for all three TFs in a dose-dependent manner with IC_50_ values ranging from 60 nM to > 5 μM, with no competition when duplex consensus sequences were used (Fig. 3b and Additional file 1: S10). We then studied PDS competition with TFs at G4 sites in K562 chromatin. We used isolated nuclei that maintain transcriptional activity [41], chromatin organization [42], and TF binding profiles [43] to improve control of small molecule dosing and adapted a ChIP approach for native, rather than cross-linked, chromatin for profiling TF binding [43] (Fig. 3c). TF occupancy at known endogenous G4 structures (from TF ChIP-seq and G4 ChIP-seq) was measured via ChIP-qPCR (Additional file 1: Table S5). PDS treatment caused a substantial reduction in SP2, NRF1, and FUS occupancy (47–71%) at the G4 sites tested (Fig. 3d). No changes were observed in occupancy for the control (non-G4 binding) TFs FOXA1 and CTCF (Fig. 3e). SP2, NRF1, and FUS binding to G4 sites in chromatin is thus reduced by a competing G4 ligand, as would be expected by TF recruitment to a G4 structure. For SP2, an IC_50_ value of ~ 60 μM was estimated from dose response experiments (Fig. 3f) and agrees with a one-site direct competition model with TF affinity of ~ 10 nM and nuclear TF protein concentration of ~ 1.5 μM (see Additional file 7: Supplemental Discussion).

### G4s are hubs for the recruitment of TFs to enhance transcription

**Fig. 4:**
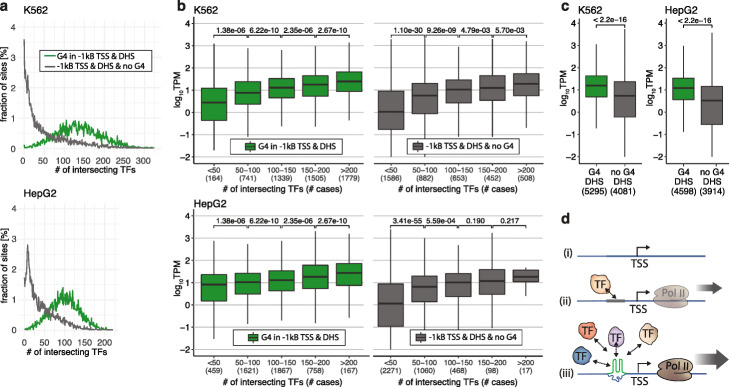
G4s are hubs for the recruitment TFs to enhance transcription. Throughout panels **a**–**c** gene endogenous G4 in promoters accessible in open chromatin (− 1 kb upstream TSS, DHS positive) are colored in green whereas promoters lacking an endogenous G4 are represented in gray. **a** Endogenous G4s mark genomic regions that are highly occupied by TFs. Proportion of G4s overlapping with multiple different TFs in K562 cells (top) and HepG2 cells (bottom). **b** Distributions of transcript levels split by the number of TFs binding at G4s in promoters or at promoters lacking G4s in K562 (top) and HepG2 cells (bottom) (unpaired Wilcoxon test). The number of cases (shown in brackets) for higher TF occupancy is substantially higher for G4s. **c** The average transcriptional output (displayed in transcripts per million (TPM), log_10_ scale) is compared for genes with and without endogenous G4s in promoters in K562 (left) and HepG2 cells (right) (unpaired Wilcoxon test). **d** A model for how endogenous G4s can enhance occupancy by multiple TFs at promoters: (i) Repressed promoters are unoccupied by TFs. (ii) Double-stranded DNA consensus binding sites recruit particular TFs to promoters resulting in active transcription. (iii) G4s can recruit numerous different TFs causing even more actively transcribed genes

We noted that a considerable number of TFs bind to the same G4 structures both in vitro (Fig. 2a and Additional file 1: S7) and in chromatin (Fig. 3d and Additional file 1: S8). In K562 and HepG2 chromatin, most endogenous G4s (located in promoters accessible in open chromatin) overlap with considerably more TF binding sites than promoters lacking endogenous G4s (Fig. 4a). In previous studies, thousands of high-occupancy targets to which many different TFs bound were highlighted in mammalian genomes [44, 45]. While this observation has partly been attributed to technical ChIP artifacts at highly expressed genes and GC-rich loci [46, 47], recent studies suggest that this binding phenomenon is not an artifact and is based on direct TF-DNA interactions [48, 49]. A major point of contention is the finding that many TF binding sites do not match known consensus motifs [47]. We now hypothesize that DNA secondary structures such as G4s are a recognition feature that explains how multiple TFs bind to the same genomic loci. Furthermore, we found that as the number of TFs binding at endogenous G4s increased so did RNA Polymerase 2 occupancy and transcriptional activity (Fig. 4b and Additional file 1: Fig. S11). A similar correlation was observed for promoters lacking G4s, but it should be noted that endogenous G4s are considerably more occupied by a greater number of TFs (see different categories in Fig. 4b). This now provides a mechanistic explanation of why genes marked by endogenous promoter G4s show higher overall transcriptional levels (*P* < 2.22 × 10^− 16^, unpaired Wilcoxon test) (Fig. 4c), as previously observed in human epidermal keratinocyte cells [16]. Taken together, we propose that endogenous G4s provide non-canonical docking sites for many different TF complexes, to enable more frequent and productive interactions through increased RNA Polymerase 2 recruitment leading to greater transcriptional output (Fig. 4d). It is unlikely that dozens or hundreds of TFs simultaneously co-occupy a genomic locus in a single large molecular complex in the same cell [48]. While certain TFs may bind their targets cooperatively [4], it is more likely that many TFs can dynamically compete for binding to the same G4 locus. In a large population of cells, this would result in the apparent co-localization at the same site due to signal averaging across the cellular population.

## Discussion

A fundamental feature of transcriptional regulation is the ability of TFs to recognize specific DNA binding sites. In this study, we present an alternative view to the established model of consensus sequence motif binding whereby endogenous G4 structures in promoters frequently serve as docking sites for TFs in human chromatin. Our work supports that DNA secondary structure recognition is an important mode by which TFs can read the genome. By mapping the G4 landscape in two human cancer cell lines and comparing these to hundreds of TF binding maps, we reveal that many TFs are highly enriched at endogenous G4 sites. This enrichment is comparable to that of dsDNA consensus binding making it highly probable that G4s have a similar capacity to recruit TFs in a cellular context.

Validating this model, we observe that several TFs bind G4s with affinities comparable to their consensus dsDNA both in vitro and in a chromatin context and that small molecule ligands can displace TFs from endogenous G4s, but not consensus dsDNA sites. Given that ENCODE has only mapped ~ 450 out of ~ 2800 potential TFs in K562 and HepG2 cells [1], there is every prospect that many more TFs will be recruited to endogenous G4.

Recently, endogenous expression of a small, engineered G4-binding protein was reported for detection of DNA G4s via ChIP-seq in human cells [50]. This alternative mapping approach observed G4s to be enriched at promoters, associated with highly expressed genes, and enrichment of certain proteins (FUS, TAF15, RBM14, TARDBP, HNRNPK, PCBP1) at G4 loci. In contrast to G4 ChIP-seq on fixed chromatin, the study mapped over 100,000 G4s and observed considerable G4 formation downstream of the TSS in addition to promoter G4s. Endogenous expression of a probe may be able to detect weaker, more transient G4s. However, it may also perturb the endogenous G4 landscape and shift the equilibrium to stabilize G4s that do not normally form under physiological conditions.

A remaining challenge in the understanding of mechanisms that regulate transcription is how a large number of different TFs bind to the same genomic site and cannot be explained by the presence of their respective consensus motifs [1]. For some TFs, our work gives an immediate explanation into how this might be resolved through TF recruitment to G4 secondary structures rather than dsDNA consensus motifs. Furthermore, TF recruitment by G4s may explain the recognition mode for TFs with non-canonical binding properties. For example, recruitment of SP2, a TF with strong G4 association, is thought to be independent of its zinc finger dsDNA-binding domain and requires only a glutamine-rich, positively charged N-terminal region for binding [51]. Further structural investigation into of TF-G4 complexes [21] will be needed to unravel the molecular details of how TFs bind G4 structures.

Based on computationally predicted G4 forming sequences, earlier work has proposed that G4s may interfere with TF binding causing transcriptional repression and that G4s may need to be resolved by G4 binding proteins to facilitate transcription [52–54]. In contrast, endogenous promoter G4s are predominantly found at highly active genes [16, 17]. Here, we now show that in fact several TFs can selectively bind G4s, with little interaction with corresponding dsDNA sequences, and that G4s are promiscuous hubs for the binding of many different TFs. We propose a fundamental mechanism of transcriptional regulation that may apply to many genes, whereby G4 structures recruit a multitude of TFs causing more frequent engagement of TFs in promoters and thereby stimulating transcriptional output (Fig. 4d). Further functional studies are required to ascertain whether there is a universally positive role of promoter G4s in transcription and to explore the details of mechanisms that maintain the endogenous G4 landscape in chromatin [55]. Alternative DNA structures should thus be seriously considered as a means to recruit TFs.

## Materials and methods

### Cell culture

Mycoplasma-free human chronic myelogenous leukemia K562 cells (CCL-243) derived from a 53-year-old female were purchased from ATCC. HepG2 (HB-805) cells derived from a 15-year-old male were kindly provided by M. Narita (CRUK Cambridge Institute, University of Cambridge). Both cell lines were grown in accordance with ENCODE cell culture protocols and periodically tested for mycoplasma contamination and identity confirmed by STR typing. Briefly, K562 cells were cultured in RPMI1640 (Glutamine plus, Life Technologies) supplemented with 10% of fetal bovine serum (Life Technologies) at 37 °C in 5% CO_2_. HepG2 were grown in DMEM (high glucose without sodium pyruvate, Life Technologies) supplemented with 10% of fetal bovine serum (Life Technologies) at 37 °C in 5% CO_2_.

### Affinity enrichment and WES analysis

Exponentially growing K562 cells were lysed by swelling and mechanical force using hypotonic buffer (20 mM HEPES pH 7.4, 10 mM NaCl, 3 mM MgCl_2_, 0.2 mM EDTA, 1 mM dithiothreitol (DTT) containing complete protease inhibitor cocktail (PIC) (Thermo Fisher, cat. no. 87786)). Nuclei were then collected by centrifugation, lysed in high salt buffer (20 mM HEPES pH 7.4, 500 mM NaCl, 3 mM MgCl_2_, 0.2 mM EDTA, 0.5% NP40, 1 mM DTT and PIC), and sonicated in a Diagenode Bioruptor Plus (5 cycles 30 s each, 30 s ON and 30 s OFF at high setting). Protein concentrations were assessed using a Direct Detect infrared spectrometer (Merck).

For affinity enrichments (AEs), 50 μL of a slurry of streptavidin magnetic beads (Promega, cat. no. Z5481) was blocked in pull-down buffer (25 mM HEPES, 10.5 mM, 110 mM KCl, 1 mM MgCl_2_, 0.01 mM ZnCl_2_, 10% glycerol, 0.01% Igepal C-630, 1 mM DDT) containing 3% BSA and bound to folded, biotinylated oligonucleotides. Magnetic beads were incubated with ~ 0.25 mg of nuclear lysate in 250 μL pull-down buffer containing PIC and 0.2 g/L salmon sperm DNA at 4 °C overnight and washed three times with pull-down buffer. For competition binding experiments, incubations were performed in the presence of respective concentrations of the G4 ligand pyridostatin (PDS) [39]. The magnetic beads were then resuspended in 25 μL NuPAGE LDS sample buffer (Invitrogen, cat. no. NP0007) and heated to 70 °C for 10 min. Next, 1 μL of the 25 μL AEs in LDS sample buffer were analyzed via capillary-based immunoassays on a Wes Protein Simple Western System (ProteinSimple) according to the manufacturer’s protocol (https://proteinsimple.com/) using an anti-rabbit, anti-mouse, or anti-goat detection module and corresponding antibodies (Additional file 1: Table S4). Bands were quantified as area-under-the-curve using Compass software (ProteinSimple).

### G-quadruplex ChIP-seq

ChIP-seq for G-quadruplex structures (G4-ChIP-seq) in K562 and HepG2 cells was performed using the G4-specific antibody BG4 essentially as described previously [24]. Previous data for G4 ChIP-seq of K562 cells (NCBI GEO GSE107690) were also considered.

### Native TF ChIP and G4 ligand treatment

Native ChIP for TFs was adapted from established protocols for yeast and drosophila [43, 56]. For each ChIP 1 × 10^7^ log phase, K562 cells were pelleted by centrifugation (250*g*, 4 °C, 5 min) and washed twice with PBS and resuspended in TM2+ buffer (10 mM Tris, pH 7.5, 10 mM NaCl, 2 mM MgCl_2_, PIC) to a concentration of 2 × 10^8^ cells per mL, followed by addition of an equal amount of TM2+ containing 1.0% (v/v) tween-20 and intermittent vortexing for 10 min. To release nuclei, the cell suspension was homogenized in an all-glass Dounce homogenizer with 10 strokes of a “tight” pestle. Nuclei were then collected at 1000*g*, washed with TM2+, and resuspended in digestion buffer (10 mM Tris, pH 7.5, 10 mM NaCl, 1 mM CaCl_2_, 2 mM MgCl_2_, PIC) to an approximate DNA concentration of ~ 0.5 mg/mL (based on A_260_). Next, 125 μL nuclei were preheated at 37 °C for 3 min and incubated for 5 min with 250 U of micrococcal nuclease (MNase; NEB, cat. no. M0247). Digestion was stopped by addition of 5 mM EGTA and nuclei transferred to ice. The salt concentration was then adjusted to 150 mM NaCl, and nuclei were treated with a respective concentration of PDS [39] or DMSO at 37 °C for 10 min, followed by incubation on ice for 5 min. Nuclei were disrupted and chromatin solubilized by passing through a 26-gauge needle (10×). Soluble chromatin solution (S1) was separated from the insoluble pellet by centrifugation (10,000*g*, 10 min, 4 °C) and the pellet resuspended in 140 μL ChIP buffer (10 mM Tris, pH 7.5, 150 mM NaCl, 2 mM MgCl_2_, 2 mM EGTA, 0.1% Triton X-100) and incubated for 2 h at 10 °C with rotation. Salt-extracted chromatin was then clarified by centrifugation (16,000*g*, 10 min, 4 °C) and the supernatant retained (S2). Fractions S1 and S2 were combined for ChIP reactions, 1% was kept at 4 °C as input control, while 3 μg of antibody was added to the ChIP reaction and incubated for 12 h at 4 °C. Next, 25 μL of Protein G Dynabeads beads (Thermo Fisher, cat. no. 10004D) pre-blocked with 5 g/L BSA in PBS were incubated with the ChIP reaction and washed twice with wash buffer (10 mM Tris, pH 7.4, 150 mM NaCl, 0.75 mM EDTA). The beads were then resuspended in 91 μL elution buffer (10 mM Tris, pH 7.4, 50 mM NaCl, 0.1 mM EDTA) and sequentially incubated with 2 μg RNase A (Ambion, cat. no. AM2271) for 30 min at 37 °C, 100 μg proteinase K (Ambion, cat. no. AM2546) and 1% SDS at 65 °C for 30 min, and eluted DNA was purified from supernatant using a MinElute kit (Qiagen, cat. no. 28206).

### TF native ChIP-qPCR

Eluted DNA from native TF ChIP reactions was used to quantify TF enrichment via qPCR, using Fast SYBR PCR mix (Thermo Fisher, cat. no. 4385610), with a Bio-Rad CFX384 quantitative PCR machine. Cycling conditions were 95 °C for 20 s followed by 40 cycles of 3 s at 95 °C and 30 s at 60 °C. Based on ENCODE ChIP-seq data sets, primer pairs targeting TF and G4 ChIP positive and negative regions were used (Additional file 1: Table S5). Relative enrichments were derived with respect to their inputs and normalized to a TF- and G4-free enhancer control region from the TMCC1 gene (Additional file 1: Table S5).

### Other methods

Other standard methods [oligonucleotide folding, circular dichroism spectroscopy, enzyme-linked immunosorbent assay] as well as oligonucleotide and primer sequences are reported in Additional file 7: Supplemental Information.

## Supplementary Information

**Additional file 1: Fig. S1.** Endogenous G4 landscape in human K562 and HepG2 cells. **Fig. S2.** Genomic association of TFs and endogenous G4s is independent of the genomic regions used for randomization and the cell line. **Fig. S3.** TF binding is independent of G-richness. **Fig. S4.** R-loops vs. endogenous G4s. **Fig. S5.** Double-stranded DNA consensus binding motifs vs. endogenous G4s. **Fig. S6.** Structural verification of oligonucleotides used in this study. **Fig. S7.** TFs selectively bind to G4 structures. **Fig. S8.** TFs are recruited to G4s in chromatin. **Fig. S9.** Structural specificity of TF-G4 interactions. **Fig. S10.** G4 ligands compete with TFs for binding to G4 structures. **Fig. S11.** RNA Polymerase 2 occupancy depends on TF occupancy, but not on G4s. **Table S1.** DNA oligonucleotides used in this study. **Table S2.** Western-blot quantification corresponding to Fig. 2a and S7*.* **Table S3.** Western-blot quantification corresponding to Fig. 2b*.* **Table S4.** Antibodies used in this study. **Table S5.** qPCR control regions for TF native ChIP experiments.

**Additional file 2: Table S1.** Randomization of G4 ChIP-seq peaks different workspaces contrasted to TF ChIP-seq peaks from ENCODE for K562 cells.

**Additional file 3: Table S2.** Randomization of G4 ChIP-seq peaks different workspaces contrasted to TF ChIP-seq peaks from ENCODE for HepG2 cells.

**Additional file 4: Table S3.** Enrichment at G4 ChIP for TFs that have been mapped in both K562 and HepG2. The maximum enrichment was used if TFs have been mapped multiple times.

**Additional file 5: Table S4.** Randomization of control sites (potential G4, open chromatin, promoter&5’UTR, no endogenous G4) in open chromatin. Genomic associations of endogenous and control sites are contrasted for K562 cells.

**Additional file 6: Table S5.** Randomization of predicted consensus dsDNA binding sites (from JASPAR) or endogenous G4s in promoters / open chromatin promoters. Enrichment at TF chromatin binding sites is contrasted for K562 cells.

**Additional file 7.** Supplemental Information (Supplemental Methods; Supplemental Data Analysis; Supplemental Discussion.).

**Additional file 8.** Uncropped western blotting analysis.

**Additional file 9.** Review history.

## Data Availability

A detailed description of bioinformatics and data analysis is reported in SI Data analysis. The data reported in this paper are available at the NCBI GEO repository under accession number GSE145090, https://www.ncbi.nlm.nih.gov/geo/query/acc.cgi?acc=GSE145090 [23]. Results from the genomic association analysis including the corresponding ENCODE accession numbers are included in Additional files 2 and 3: Supplemental Data Table S1 and S2. All scripts are available on github, https://github.com/sblab-bioinformatics/G4-vs-TFs [57].

## References

[1] Lambert SA, Jolma A, Campitelli LF, Das PK, Yin Y, Albu M, Chen X, Taipale J, Hughes TR, Weirauch MT (2018). The human transcription factors. Cell..

[2] Badis G, Berger MF, Philippakis AA, Talukder S, Gehrke AR, Jaeger SA, Chan ET, Metzler G, Vedenko A, Chen X, Kuznetsov H, Wang CF, Coburn D, Newburger DE, Morris Q, Hughes TR, Bulyk ML (2009). Diversity and complexity in DNA recognition by transcription factors. Science..

[3] Yan J, Enge M, Whitington T, Dave K, Liu J, Sur I, Schmierer B, Jolma A, Kivioja T, Taipale M, Taipale J (2013). Transcription factor binding in human cells occurs in dense clusters formed around cohesin anchor sites. Cell..

[4] Jolma A, Yin Y, Nitta KR, Dave K, Popov A, Taipale M, Enge M, Kivioja T, Morgunova E, Taipale J (2015). DNA-dependent formation of transcription factor pairs alters their binding specificity. Nature..

[5] Wang J, Zhuang J, Iyer S, Lin XY, Greven MC, Kim BH, et al. Factorbook.org: A Wiki-based database for transcription factor-binding data generated by the ENCODE consortium. Nucleic Acids Res. 2013;41:171–6. 10.1093/nar/gks1221. 10.1093/nar/gks1221 PMC3531197 23203885

[6] Slattery M, Zhou T, Yang L, Dantas Machado AC, Gordân R, Rohs R (2014). Absence of a simple code: how transcription factors read the genome. Trends Biochem Sci.

[7] Seeman NC, Rosenberg JM, Rich A (1976). Sequence-specific recognition of double helical nucleic acids by proteins. Proc Natl Acad Sci.

[8] Rohs R, West SM, Sosinsky A, Liu P, Mann RS, Honig B (2009). The role of DNA shape in protein-DNA recognition. Nature..

[9] Abe N, Dror I, Yang L, Slattery M, Zhou T, Bussemaker HJ, Rohs R, Mann RS (2015). Deconvolving the recognition of DNA shape from sequence. Cell..

[10] Ibarra IL, Hollmann NM, Klaus B, Augsten S, Velten B, Hennig J, Zaugg JB (2020). Mechanistic insights into transcription factor cooperativity and its impact on protein-phenotype interactions. Nat Commun.

[11] Yin Y, Morgunova E, Jolma A, Kaasinen E, Sahu B, Khund-Sayeed S, et al. Impact of cytosine methylation on DNA binding specificities of human transcription factors. Science. 2017;356:eaaj2239. 10.1126/science.aaj2239. 10.1126/science.aaj2239 PMC8009048 28473536

[12] Zhu F, Farnung L, Kaasinen E, Sahu B, Yin Y, Wei B, Dodonova SO, Nitta KR, Morgunova E, Taipale M, Cramer P, Taipale J (2018). The interaction landscape between transcription factors and the nucleosome. Nature..

[13] Orenstein Y, Shamir R (2014). A comparative analysis of transcription factor binding models learned from PBM, HT-SELEX and ChIP data. Nucleic Acids Res.

[14] Varshney D, Spiegel J, Zyner K, Tannahill D, Balasubramanian S (2020). The regulation and functions of DNA and RNA G-quadruplexes. Nat Rev Mol Cell Biol.

[15] Biffi G, Tannahill D, McCafferty J, Balasubramanian S (2013). Quantitative visualization of DNA G-quadruplex structures in human cells. Nat Chem.

[16] Hänsel-Hertsch R, Beraldi D, Lensing SV, Marsico G, Zyner K, Parry A, di Antonio M, Pike J, Kimura H, Narita M, Tannahill D, Balasubramanian S (2016). G-quadruplex structures mark human regulatory chromatin. Nat Genet.

[17] Kouzine F, Wojtowicz D, Baranello L, Yamane A, Nelson S, Resch W, et al. Permanganate/S1 nuclease footprinting reveals non-B DNA structures with regulatory potential across a mammalian genome. Cell Syst. 2017;4:344–356.e7. 10.1016/j.cels.2017.01.013. 10.1016/j.cels.2017.01.013 PMC7432990 28237796

[18] Hänsel-Hertsch R, Simeone A, Shea A, Hui WWI, Zyner KG, Marsico G, Rueda OM, Bruna A, Martin A, Zhang X, Adhikari S, Tannahill D, Caldas C, Balasubramanian S (2020). Landscape of G-quadruplex DNA structural regions in breast cancer. Nat Genet.

[19] Marchetti C, Zyner KG, Ohnmacht SA, Robson M, Haider SM, Morton JP, Marsico G, Vo T, Laughlin-Toth S, Ahmed AA, di Vita G, Pazitna I, Gunaratnam M, Besser RJ, Andrade ACG, Diocou S, Pike JA, Tannahill D, Pedley RB, Evans TRJ, Wilson WD, Balasubramanian S, Neidle S (2018). Targeting multiple effector pathways in pancreatic ductal adenocarcinoma with a G-quadruplex-binding small molecule. J Med Chem.

[20] Mishra SK, Tawani A, Mishra A, Kumar A (2016). G4IPDB: a database for G-quadruplex structure forming nucleic acid interacting proteins. Sci Rep.

[21] Chen MC, Tippana R, Demeshkina NA, Murat P, Balasubramanian S, Myong S, Ferré-D’Amaré AR (2018). Structural basis of G-quadruplex unfolding by the DEAH/RHA helicase DHX36. Nature..

[22] Gerstein MB, Kundaje A, Hariharan M, Landt SG, Yan KK, Cheng C, Mu XJ, Khurana E, Rozowsky J, Alexander R, Min R, Alves P, Abyzov A, Addleman N, Bhardwaj N, Boyle AP, Cayting P, Charos A, Chen DZ, Cheng Y, Clarke D, Eastman C, Euskirchen G, Frietze S, Fu Y, Gertz J, Grubert F, Harmanci A, Jain P, Kasowski M, Lacroute P, Leng J, Lian J, Monahan H, O’Geen H, Ouyang Z, Partridge EC, Patacsil D, Pauli F, Raha D, Ramirez L, Reddy TE, Reed B, Shi M, Slifer T, Wang J, Wu L, Yang X, Yip KY, Zilberman-Schapira G, Batzoglou S, Sidow A, Farnham PJ, Myers RM, Weissman SM, Snyder M (2012). Architecture of the human regulatory network derived from ENCODE data. Nature..

[23] Spiegel J, Martinez Cuesta S, Adhikari S, Hänsel-Hertsch R, Tannahill D, Balasubramanian S. G-quadruplexes are transcription factor binding hubs in human chromatin. Datasets. Gene Expression Omnibus (GEO). https://www.ncbi.nlm.nih.gov/geo/query/acc.cgi?acc=GSE145090. Accessed 23 Mar 2021. 10.1186/s13059-021-02324-z PMC8063395 33892767

[24] Hänsel-Hertsch R, Spiegel J, Marsico G, Tannahill D, Balasubramanian S (2018). Genome-wide mapping of endogenous G-quadruplex DNA structures by chromatin immunoprecipitation and high-throughput sequencing. Nat Protoc.

[25] Wanrooij PH, Uhler JP, Shi Y, Westerlund F, Falkenberg M, Gustafsson CM (2012). A hybrid G-quadruplex structure formed between RNA and DNA explains the extraordinary stability of the mitochondrial R-loop. Nucleic Acids Res.

[26] Chambers VS, Marsico G, Boutell JM, Di Antonio M, Smith GP, Balasubramanian S (2015). High-throughput sequencing of DNA G-quadruplex structures in the human genome. Nat Biotechnol.

[27] Sanz LA, Hartono SR, Lim YW, Steyaert S, Rajpurkar A, Ginno PA, Xu X, Chédin F (2016). Prevalent, dynamic, and conserved R-loop structures associate with specific epigenomic signatures in mammals. Mol Cell.

[28] Lee CY, McNerney C, Ma K, Zhao W, Wang A, Myong S. R-loop induced G-quadruplex in non-template promotes transcription by successive R-loop formation. Nat Commun. 2020;11:1–15. 10.1038/s41467-020-17176-7. 10.1038/s41467-020-17176-7 PMC7341879 32636376

[29] Chen L, Chen JY, Zhang X, Gu Y, Xiao R, Shao C, et al. R-ChIP Using Inactive RNase H Reveals Dynamic Coupling of R-loops with Transcriptional Pausing at Gene Promoters. Mol Cell. 2017;68:745–57.e5. 10.1016/j.molcel.2017.10.008. 10.1016/j.molcel.2017.10.008 PMC5957070 29104020

[30] Fornes O, Castro-Mondragon JA, Khan A, van der Lee R, Zhang X, Richmond PA, et al. JASPAR 2020: update of the open-access database of transcription factor binding profiles. Nucleic Acids Res. 2019;48:87–92. 10.1093/nar/gkz1001. 10.1093/nar/gkz1001 PMC7145627 31701148

[31] Xiao R, Chen J-Y, Liang Z, Luo D, Chen G, Lu ZJ, et al. Pervasive chromatin-RNA binding protein interactions enable RNA-based regulation of transcription. Cell. 2019;178:107–21.e18. 10.1016/j.cell.2019.06.001. 10.1016/j.cell.2019.06.001 PMC6760001 31251911

[32] Kutyavin IV, Lokhov SG, Afonina IA, Dempcy R, Gall AA, Gorn VV, Lukhtanov E, Metcalf M, Mills A, Reed MW, Sanders S, Shishkina I, Vermeulen NM (2002). Reduced aggregation and improved specificity of G-rich oligodeoxyribonucleotides containing pyrazolo [3,4-d] pyrimidine guanine bases. Nucleic Acids Res.

[33] Kang HJ, Kendrick S, Hecht SM, Hurley LH (2014). The transcriptional complex between the BCL2 i-motif and hnRNP LL is a molecular switch for control of gene expression that can be modulated by small molecules. J Am Chem Soc.

[34] Sutherland C, Cui Y, Mao H, Hurley LH (2016). A mechanosensor mechanism controls the G-quadruplex/i-motif molecular switch in the MYC promoter NHE III1. J Am Chem Soc.

[35] Wang IX, Grunseich C, Fox J, Burdick J, Zhu Z, Ravazian N, Hafner M, Cheung VG (2018). Human proteins that interact with RNA/DNA hybrids. Genome Res.

[36] Raiber EA, Kranaster R, Lam E, Nikan M, Balasubramanian S (2012). A non-canonical DNA structure is a binding motif for the transcription factor SP1 in vitro. Nucleic Acids Res.

[37] Li L, Williams P, Ren W, Wang MY, Gao Z, Miao W, et al. YY1 interacts with guanine quadruplexes to regulate DNA looping and gene expression. Nat Chem Biol. 2021;17(2):161–8. 10.1038/s41589-020-00695-1. 10.1038/s41589-020-00695-1 PMC7854983 33199912

[38] Yagi R, Miyazaki T, Oyoshi T (2018). G-quadruplex binding ability of TLS/FUS depends on the β-spiral structure of the RGG domain. Nucleic Acids Res.

[39] Rodriguez R, Müller S, Yeoman JA, Trentesaux C, Riou JF, Balasubramanian S (2008). A novel small molecule that alters shelterin integrity and triggers a DNA-damage response at telomeres. J Am Chem Soc.

[40] Le DD, Di Antonio M, Chan LKM, Balasubramanian S (2015). G-quadruplex ligands exhibit differential G-tetrad selectivity. Chem Commun.

[41] Core LJ, Waterfall JJ, Lis JT (2008). Nascent RNA sequencing reveals widespread pausing and divergent initiation at human promoters. Science..

[42] Sardo L, Lin A, Khakhina S, Beckman L, Ricon L, Elbezanti W, Jaison T, Vishwasrao H, Shroff H, Janetopoulos C, Klase ZA (2017). Real-time visualization of chromatin modification in isolated nuclei. J Cell Sci.

[43] Kasinathan S, Orsi GA, Zentner GE, Ahmad K, Henikoff S. High-resolution mapping of transcription factor binding sites on native chromatin. Nat Methods. 2014;11:203–9. 10.1038/nmeth.2766. 10.1038/nmeth.2766 PMC3929178 24336359

[44] Yip KY, Cheng C, Bhardwaj N, Brown JB, Leng J, Kundaje A, Rozowsky J, Birney E, Bickel P, Snyder M, Gerstein M (2012). Classification of human genomic regions based on experimentally determined binding sites of more than 100 transcription-related factors. Genome Biol.

[45] Xie D, Boyle AP, Wu L, Zhai J, Kawli T, Snyder M (2013). Dynamic trans-acting factor colocalization in human cells. Cell..

[46] Wreczycka K, Franke V, Uyar B, Wurmus R, Bulut S, Tursun B, et al. HOT or not: examining the basis of high-occupancy target regions. Nucleic Acids Res. 2019;47(11):5735–45. 10.1093/nar/gkz460. 10.1093/nar/gkz460 PMC6582337 31114922

[47] Gheorghe M, Sandve GK, Khan A, Chèneby J, Ballester B, Mathelier A (2019). A map of direct TF-DNA interactions in the human genome. Nucleic Acids Res.

[48] Partridge EC, Chhetri SB, Prokop JW, Ramaker RC, Jansen CS, Goh S (2020). Occupancy maps of 208 chromatin-associated proteins in one human cell type. Nature..

[49] Ramaker RC, Hardigan AA, Goh S-T, Partridge EC, Wold B, Cooper SJ, Myers RM (2020). Dissecting the regulatory activity and sequence content of loci with exceptional numbers of transcription factor associations. Genome Res.

[50] Zheng K, Zhang J, He Y, Gong J, Wen C, Chen J, Hao YH, Zhao Y, Tan Z (2020). Detection of genomic G-quadruplexes in living cells using a small artificial protein. Nucleic Acids Res.

[51] Völkel S, Stielow B, Finkernagel F, Stiewe T, Nist A, Suske G. Zinc finger independent genome-wide binding of Sp2 potentiates recruitment of histone-fold protein Nf-y distinguishing it from Sp1 and Sp3. PLoS Genet. 2015;11:1–25. 10.1371/journal.pgen.1005102. 10.1371/journal.pgen.1005102 PMC4368557 25793500

[52] Thakur RK, Kumar P, Halder K, Verma A, Kar A, Parent JL, Basundra R, Kumar A, Chowdhury S (2009). Metastases suppressor NM23-H2 interaction with G-quadruplex DNA within c-MYC promoter nuclease hypersensitive element induces c-MYC expression. Nucleic Acids Res.

[53] Cogoi S, Shchekotikhin AE, Xodo LE (2014). HRAS is silenced by two neighboring G-quadruplexes and activated by MAZ, a zinc-finger transcription factor with DNA unfolding property. Nucleic Acids Res.

[54] David AP, Pipier A, Pascutti F, Binolfi A, Weiner AMJ, Challier E, Heckel S, Calsou P, Gomez D, Calcaterra NB, Armas P (2019). CNBP controls transcription by unfolding DNA G-quadruplex structures. Nucleic Acids Res.

[55] Roychoudhury S, Pramanik S, Harris HL, Tarpley M, Sarkar A, Spagnol G, et al. Endogenous oxidized DNA bases and APE1 regulate the formation of G-quadruplex structures in the genome. Proc Natl Acad Sci 2020;117(21):11409–20. 10.1073/pnas.1912355117. 10.1073/pnas.1912355117 PMC7260947 32404420

[56] Orsi GA, Kasinathan S, Zentner GE, Henikoff S, Ahmad K. Mapping regulatory factors by Immunoprecipitation from native chromatin. Curr Protoc Mol Biol. 2015;110:21.31.1–25. 10.1002/0471142727.mb2131s110. 10.1002/0471142727.mb2131s110 PMC4410783 25827087

[57] Spiegel J, Martinez Cuesta S, Adhikari S, Hänsel-Hertsch R, Tannahill D, Balasubramanian S. G-quadruplexes are transcription factor binding hubs in human chromatin. Github. 2021. https://github.com/sblab-bioinformatics/G4-vs-TFs. Accessed 23 Mar 2021. 10.1186/s13059-021-02324-z PMC8063395 33892767

