# Supplementary material for: G-quadruplexes are transcription factor binding hubs in human chromatin
Source: Genome Biol. 2021 Apr 23;22:117. doi: 10.1186/s13059-021-02324-z (PMC8063395; doi:10.1186/s13059-021-02324-z)
Supplement: Supplementary file 1 — Additional file 1: Fig. S1. Endogenous G4 landscape in human K562 and HepG2 cells. Fig. S2. Genomic association of TFs and endogenous G4s is independent of the genomic regions used for randomization and the cell line. Fig. S3. TF binding is independent of G-richness. Fig. S4. R-loops vs. endogenous G4s. Fig. S5. Double-stranded DNA consensus binding motifs vs. endogenous G4s. Fig. S6. Structural verification of oligonucleotides used in this study. Fig. S7. TFs selectively bind to G4 structures. Fig. S8. TFs are recruited to G4s in chromatin. Fig. S9. Structural specificity of TF-G4 interactions. Fig. S10. G4 ligands compete with TFs for binding to G4 structures. Fig. S11. RNA Polymerase 2 occupancy depends on TF occupancy, but not on G4s. Table S1. DNA oligonucleotides used in this study. Table S2. Western-blot quantification corresponding to Fig. 2a and S7. Table S3. Western-blot quantification corresponding to Fig. 2b. Table S4. Antibodies used in this study. Table S5. qPCR control regions for TF native ChIP experiments. [file 13059_2021_2324_MOESM1_ESM.docx]

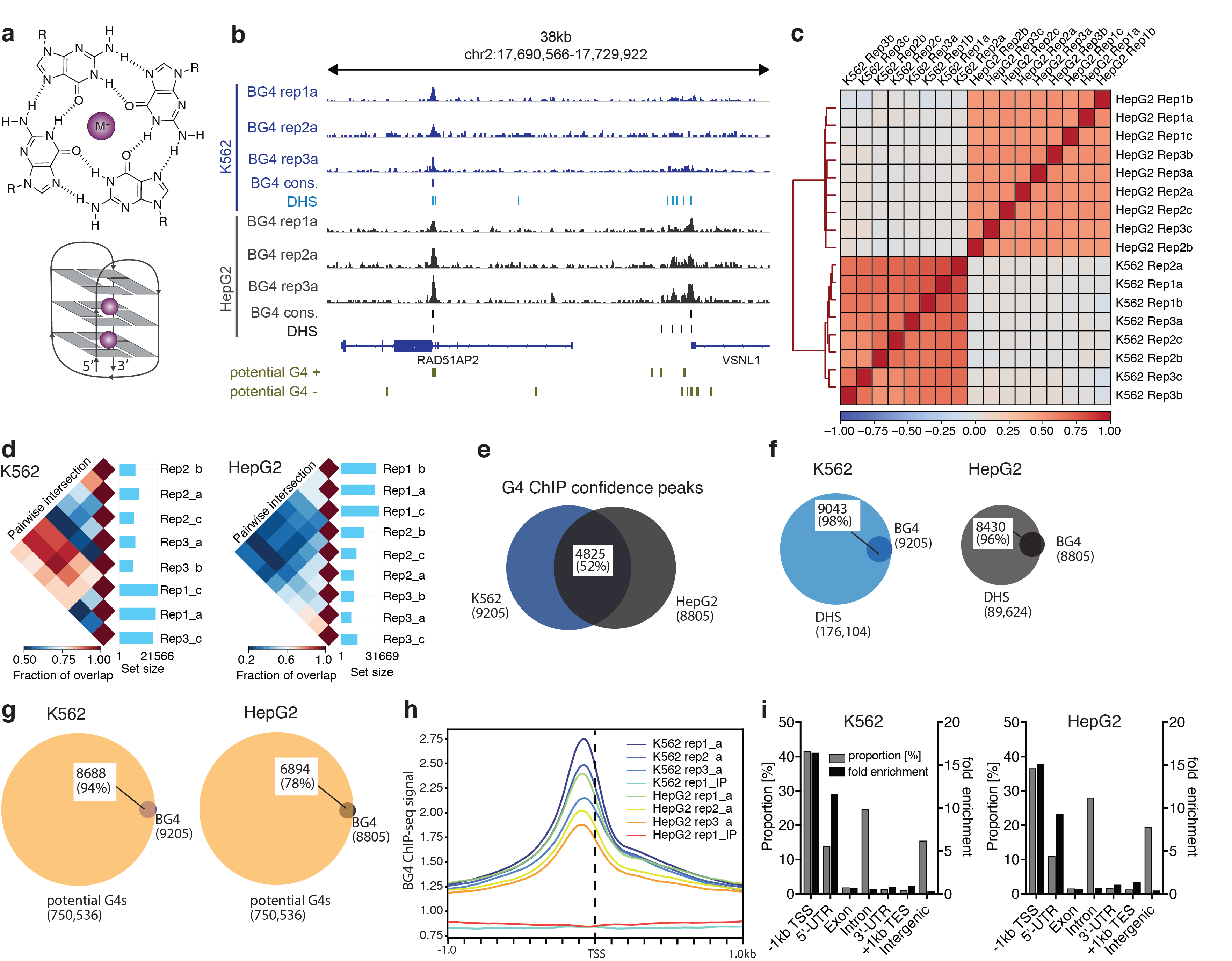


**Fig. S1.** Endogenous G4 landscape in human K562 and HepG2 cells.

**a** Top. A planar G-tetrad showing guanine bases stabilized by Hoogsteen-bonds and a central cation (purple). Bottom. Stacked G4-tetrads forming an intramolecular G-quadruplex structure.

**b** Example IGV genome browser screenshot. Tracks show three representative G4 ChIP-seq replicates in K562 and HepG2. BG4 high confidence peaks and DNase hypersensitivity sites (DHS) in both cell lines as well as potential G4 sites [1] on the forward (+) and reverse (-) strand are indicated.

**c** Correlation of read coverage in union of K562 and HepG2 G4 ChIP-seq peaks. The heatmap shows the pairwise Pearson correlation coefficient of three independent G4 ChIP-seq experiments (two to three technical replicates each) in K562 and HepG2 cells.

**d** Peak overlap of G4 ChIP-seq replicates in K562 and HepG2 cells.

**e** Overlap of G4 ChIP-seq high confidence peaks in K562 (in 5 out of 8 replicates) and HepG2 (in 6 out of 9 replicates).

**f** Endogenous G4s in K562 and HepG2 cells are exclusively found in open chromatin as shown by the overlap with DHS.

**g** Endogenous G4s are predominantly found at potential G4 sites [1].

**h** G4 ChIP-seq signal distribution around TSS in K562 and HepG2.

**i** Enrichment of G4 ChIP-seq peaks relative to expected outcomes from random shuffling of G4 ChIP-seq peaks in potential G4s, and the absolute proportion of G4 ChIP-seq peaks across different genomic features. TSS, transcription start site; 5’ untranslated region, 5’-UTR; TES, transcription end site.


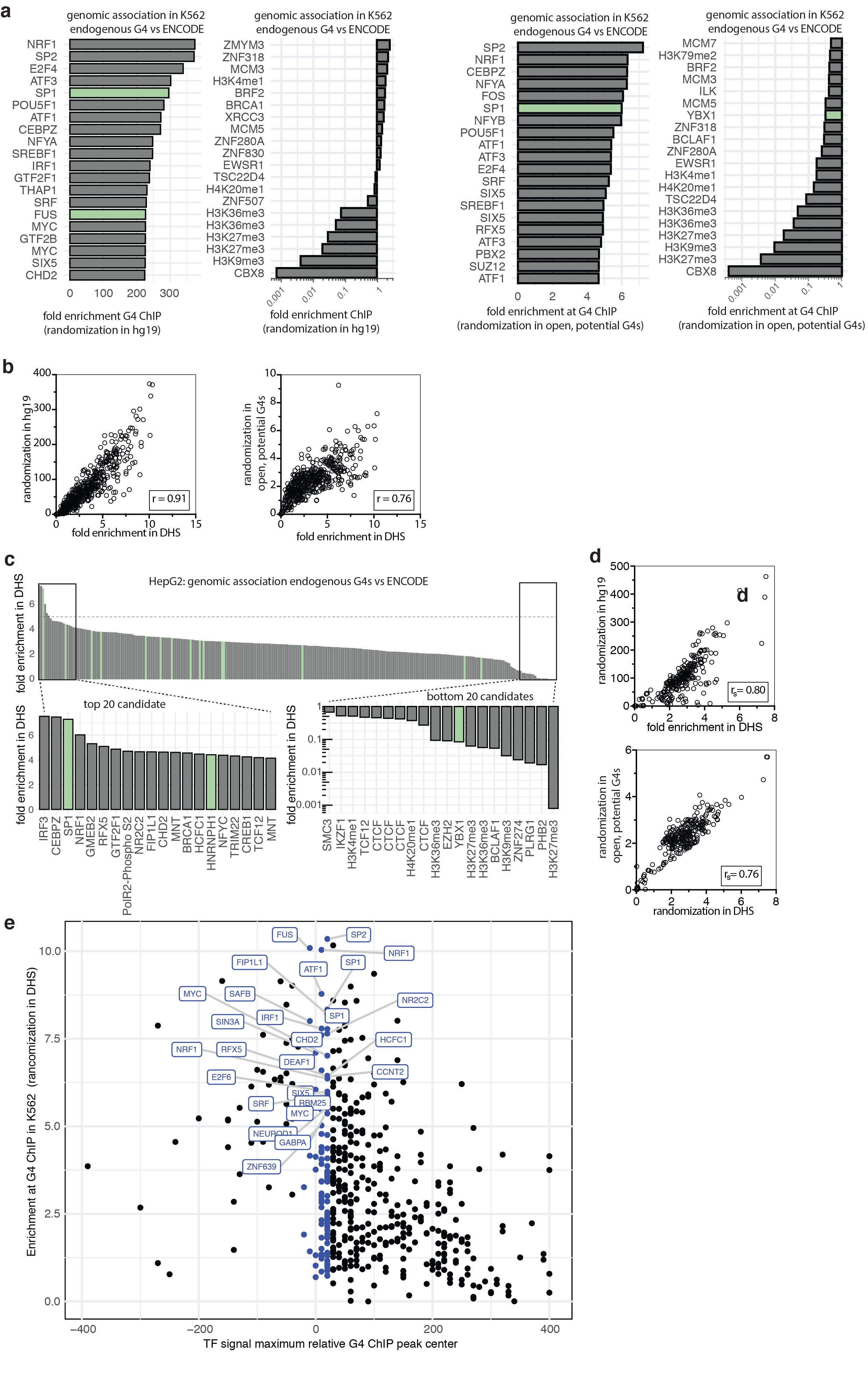


**Fig. S2.** Genomic association of TFs and endogenous G4s is independent of the genomic regions used for randomization and the cell line.

**a** Enrichment of TFs at endogenous G4s in K562 for randomization in different workspaces. The top 20 and bottom 20 candidates as well as Spearman correlation (**b**) analysis (r_s_, *****P*<0.0001) is performed with randomization in the white-listed genome and randomization in the potential G4s that are located in open chromatin, with respect to randomization in open chromatin regions (DHS).

**c** Enrichment of 278 ENCODE markers at endogenous in HepG2 for randomization in open chromatin. The top 20 and bottom 20 candidates as well as Spearman correlation (**d**) (r_s_, *****P*< 0.0001) are shown; analyses performed as in K562.

**e** TF occupancy relative to G4 ChIP-seq sites in K562. Distance of maximum TF signal relative to the G4 peak center (x-axis) and enrichment at endogenous G4s in K562 cells (y-axis) is shown. TFs centered on G4s within ±20 bp are highlighted in blue (100/488 TFs).


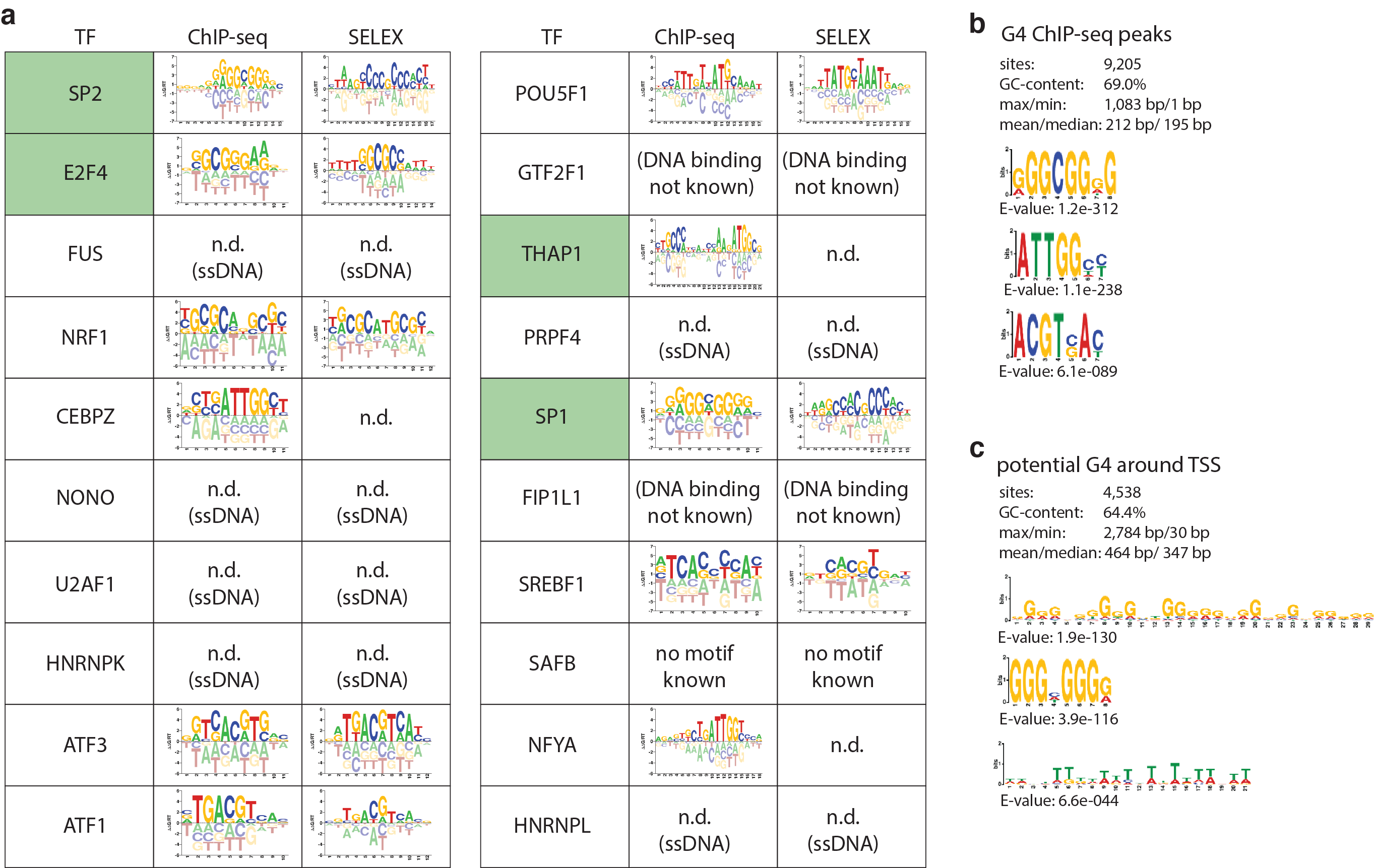


**Fig. S3.** TF binding is independent of G-richness.

**a** Consensus motifs for the top 20 candidates in K562 cells derived from ChIP-seq and SELEX experiments [2]. Many TFs (e.g. SP1, SP2, E2F4) display a G-rich binding motif (highlighted in green). Several top candidates are known to be single-strand binding proteins.

**b** Properties of endogenous G4s as detected by G4 ChIP-seq in K562 cells and **c** control data set obtained from sequences with G4-forming potential located in open chromatin around transcription start sites. The top three MEME-ChIP motifs are shown.


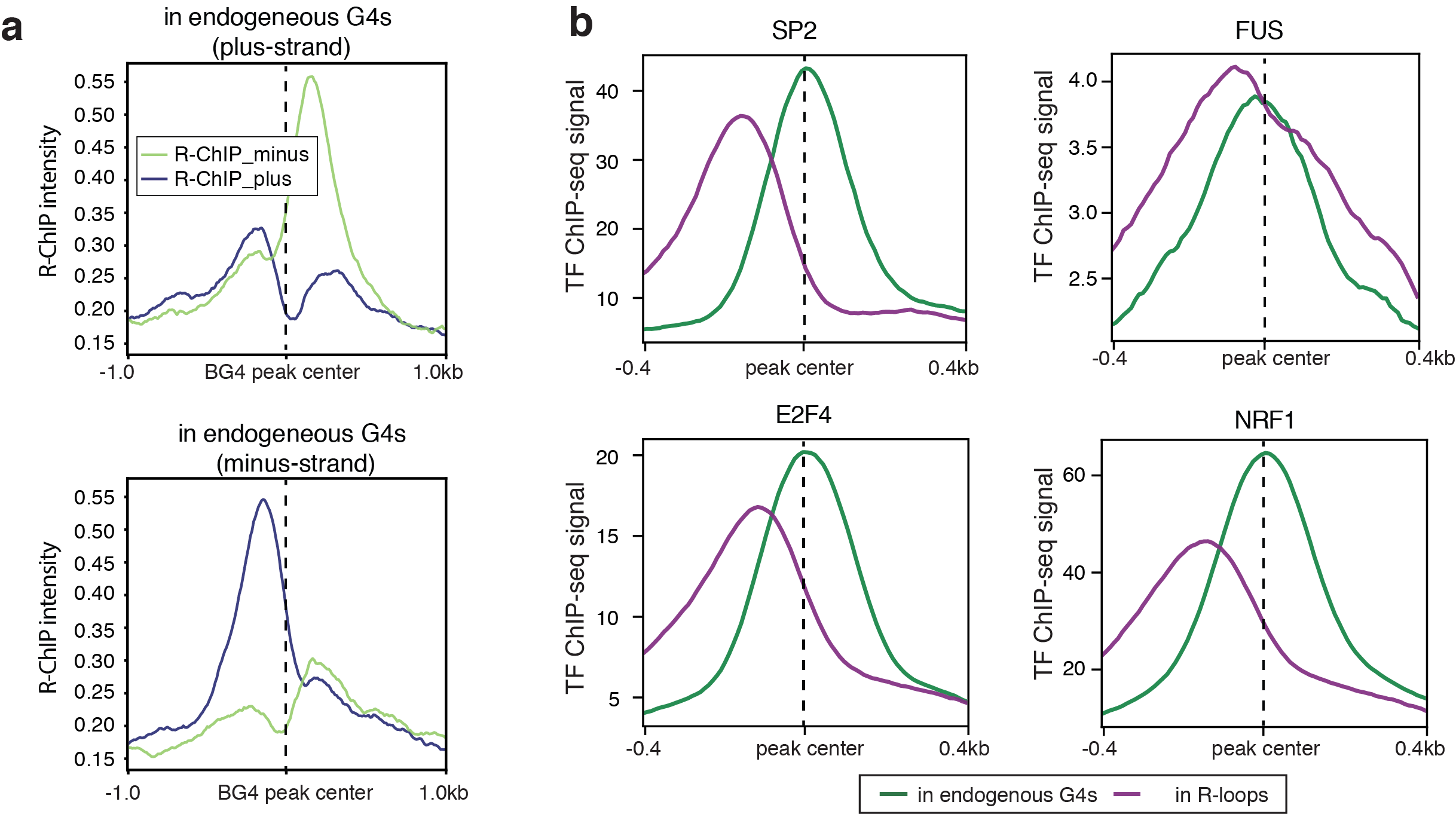


**Fig. S4.** R-loops vs. endogenous G4s.

**a** R-ChIP signal distribution around endogenous G4s in K562. The strandedness of endogenous G4s was derived from stranded data of sequences with G4 forming potential [1] (see METHODS).

**b** TF occupancy profiles of candidates SP2, E2F4, FUS, and NRF1 around endogenous G4 sites (green) and R-loops (purple).


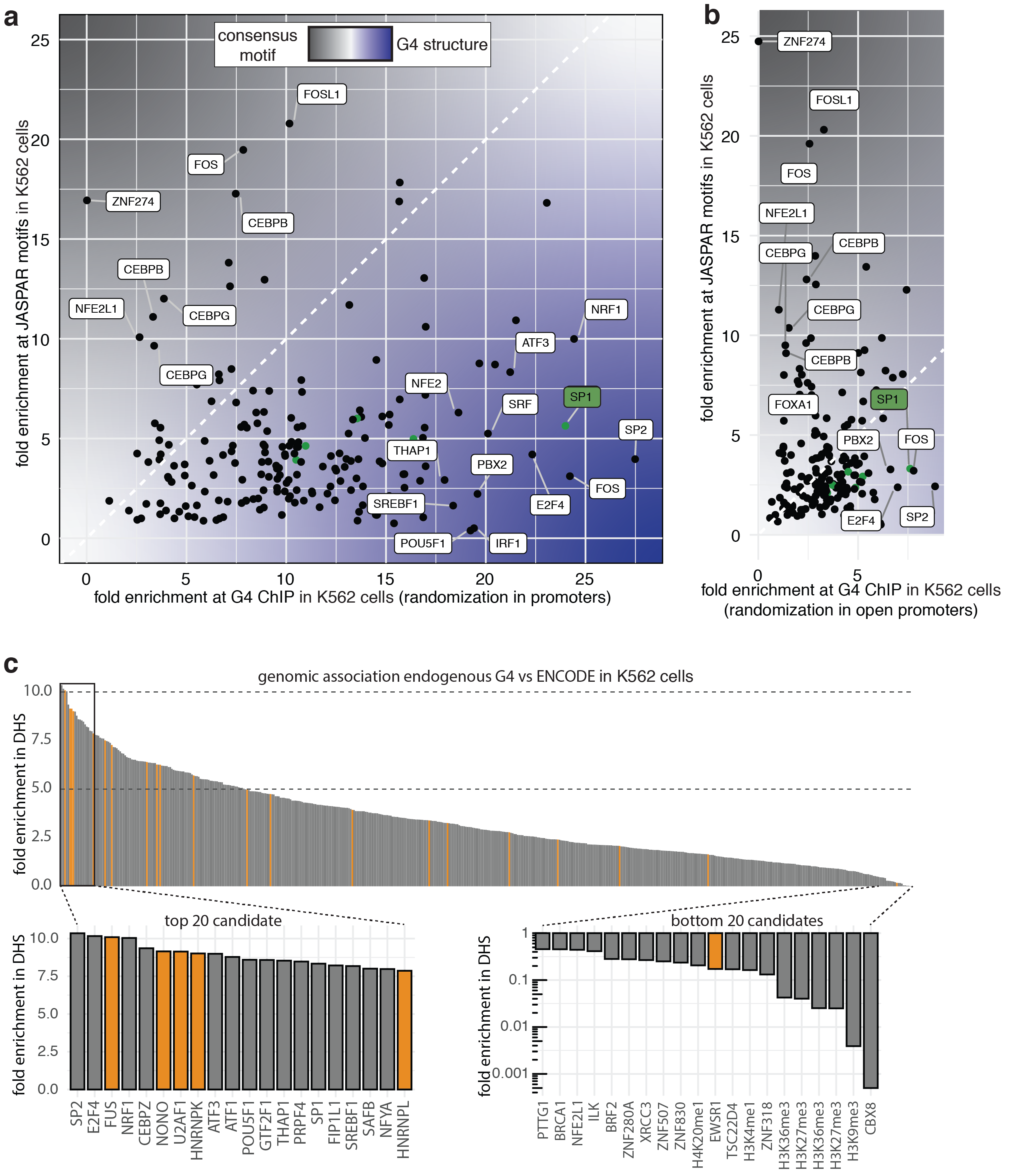


**Fig. S5.** Double-stranded DNA consensus binding motifs vs. endogenous G4s.

**a** Genomic association analysis in K562 cells showing the enrichment of TFs binding at G4 structures (x-axis) compared to consensus motif matches (y-axis) using gene promoters as genomic regions allowed for randomization.

**b** A similar, albeit weaker trend is observed when gene promoters accessible in open chromatin are used for randomization (known G4-associated proteins are highlighted in green).

**c** Many factors commonly classified as “ssDNA/RNA binding” are enriched at endogenous G4 sites (orange shading). Five of the 21 ssDNA/RNA binding proteins that have been mapped in K562 cells by ENCODE are among the top 20 G4-associated proteins after randomization in K562 open chromatin.


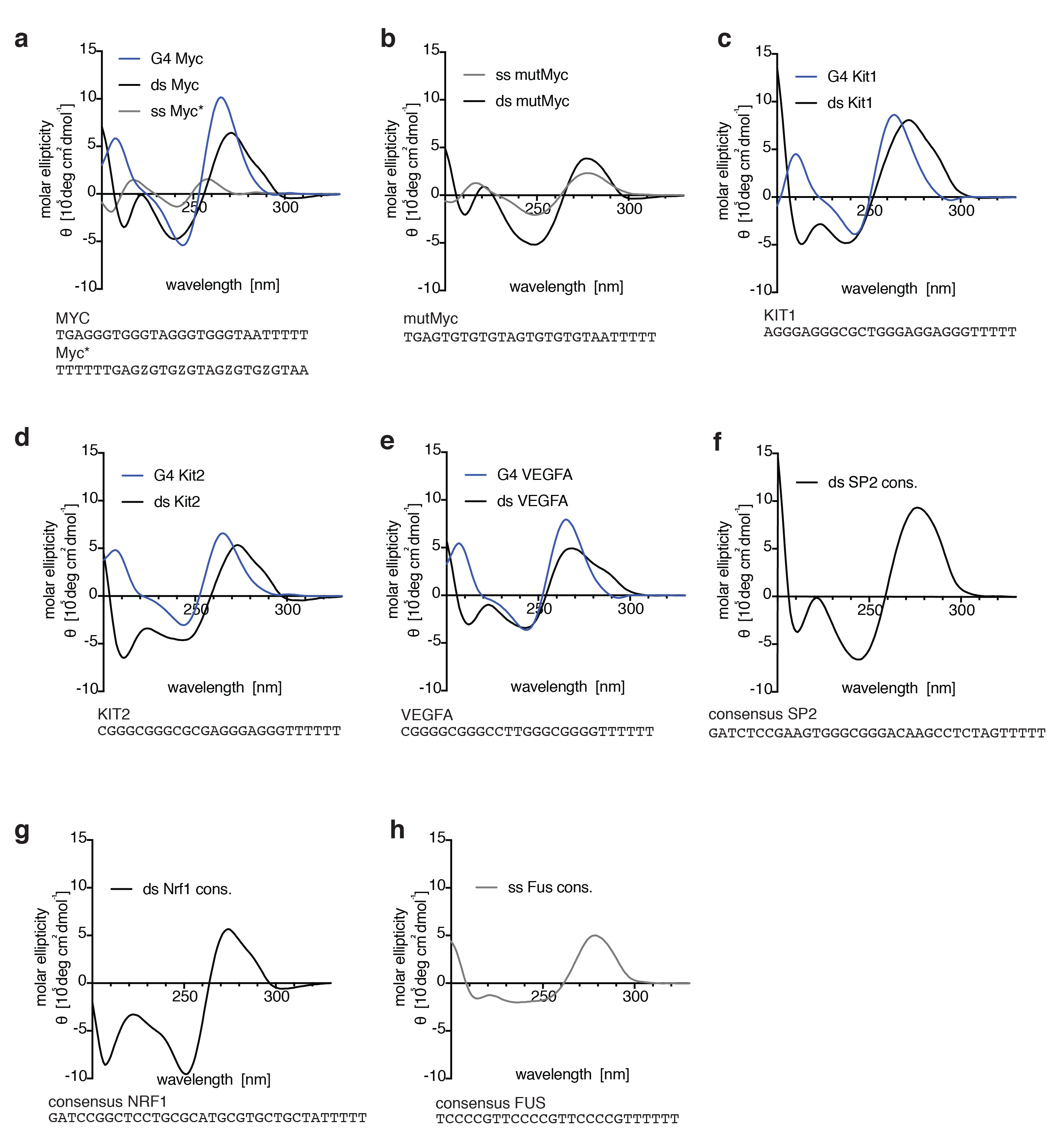


**Fig. S6.** Structural verification of oligonucleotides used in this study.

Circular dichroism spectra [3] of previously characterized (see Table S1) parallel G4 structures G4 Myc (**a**), G4 Kit1 (**c**), G4 Kit2 (**d**) and G4 VEGFA (**e**) show characteristic positive peaks at ~260 nm and 210 nm. Single-stranded, (**b**) mutated Myc and 8-aza-7-deazaguanosine (Z) substituted Myc* (**a**) do not have the capacity to form G4s and therefore have lost these characteristic signals. Double-stranded DNA is characterized by a positive long wavelength band at 260nm - 280nm and a negative band at 245 nm (**a-g**). **f** Random coiled single-stranded DNA.


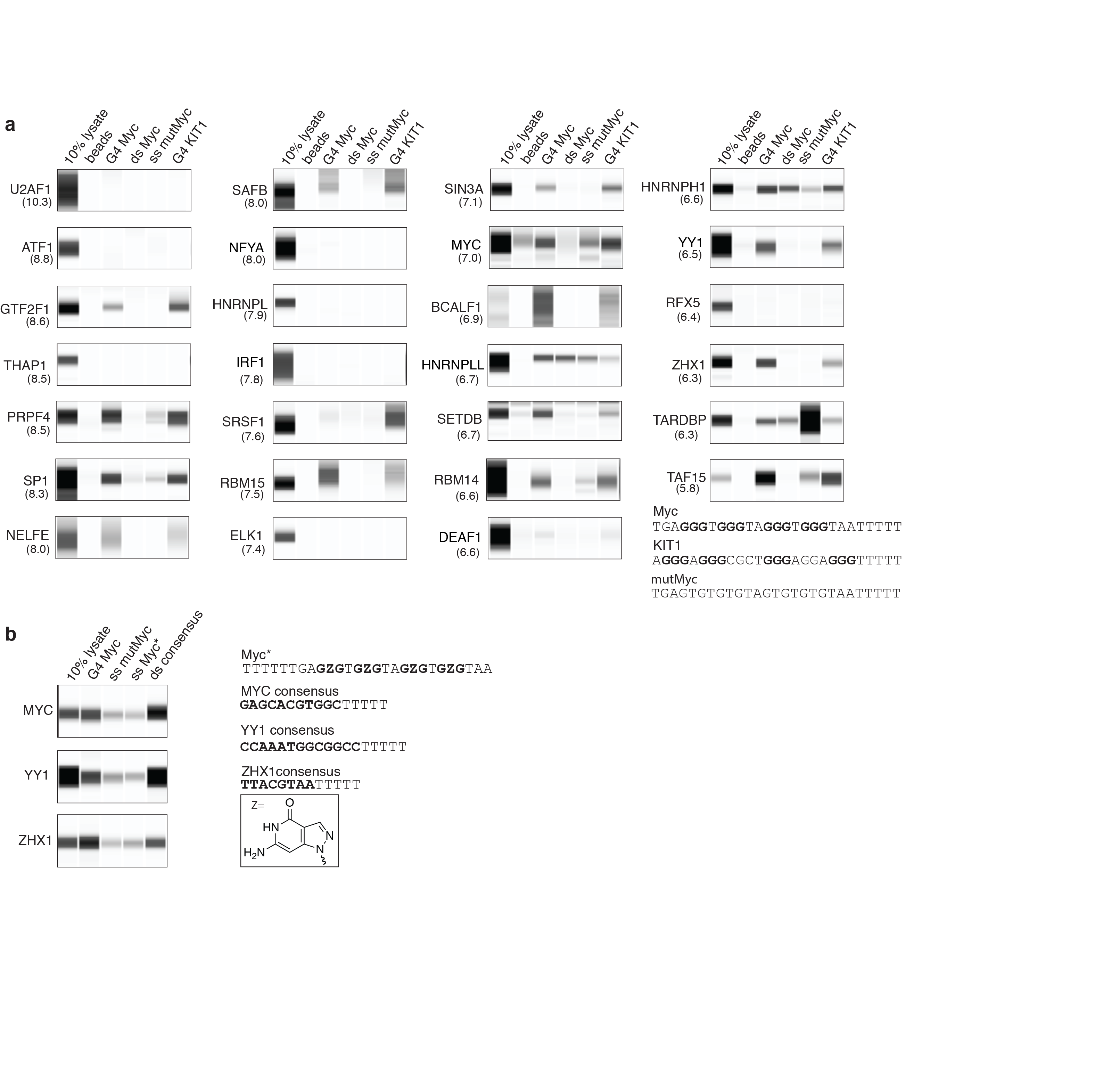


**Fig. S7** TFs selectively bind to G4 structures.

**a** Affinity pull-down western blot analysis of different G4 oligonucleotides and control sequences. Genomic enrichment at endogenous G4s in K562 for randomization in open chromatin is shown in brackets.

**b** Affinity pull-down of MYC, YY1 and ZHX1 including a single-stranded, G-rich oligomer unable to form a G4 structure (ss Myc*) and respective double-stranded DNA consensus sequences.


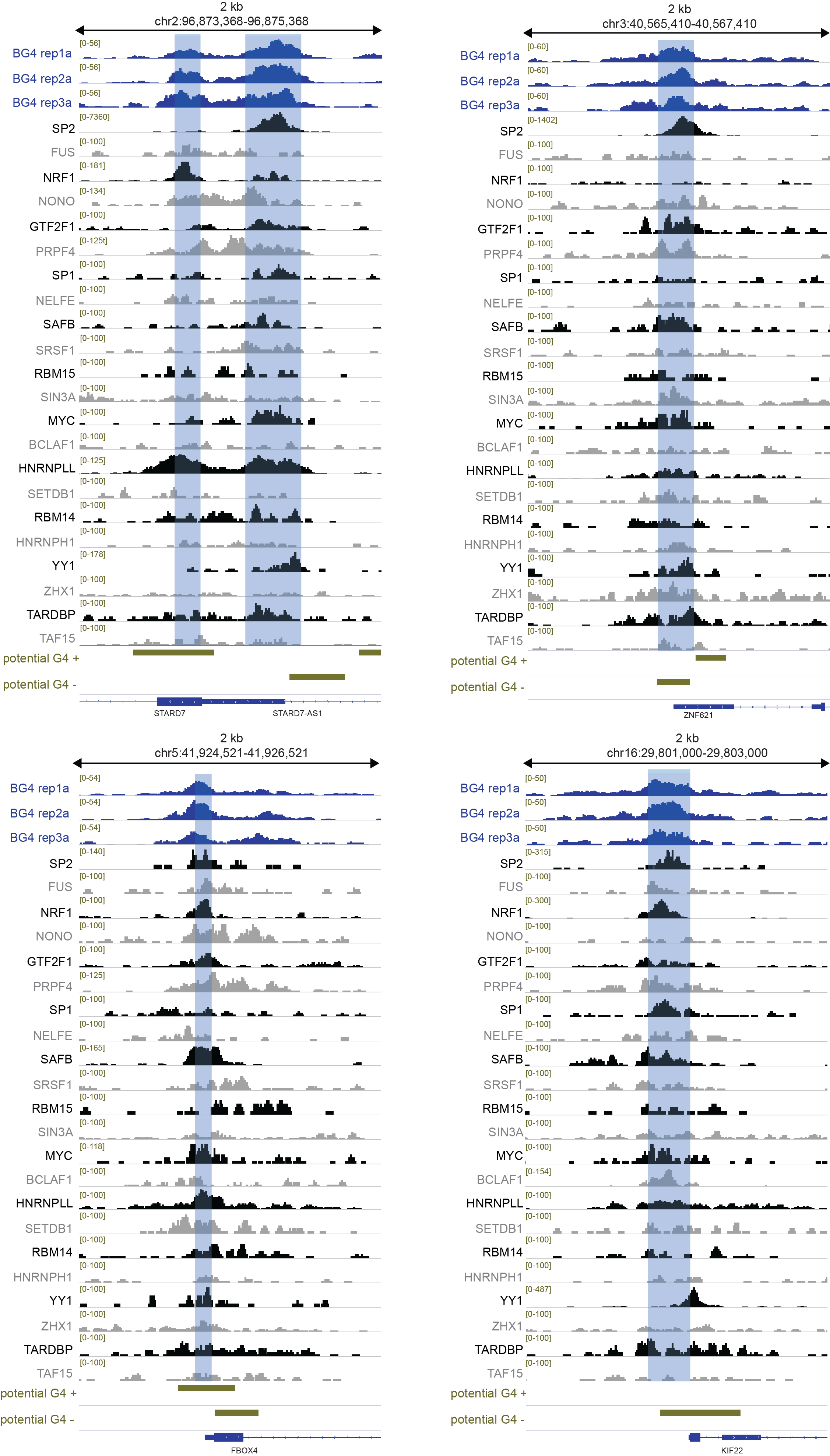


**Fig. S8.** TFs are recruited to G4s in chromatin.

ChIP-seq density from K562 cells for BG4 ChIP-seq (blue, three representative replicates) and ENCODE ChIP-seq (black and grey) for TFs at four different promoters. The 22 TFs (one representative replicate each) are shown that have been validated to bind DNA G4 oligonucleotides from K562 nuclear lysate. Potential G4 sites [10] on the forward (+) and reverse (-) strand (ochre) as well as BG4 high confidence peak sites (blue shade) are indicated. (SP2 (ENCFF153ANI), FUS (ENCFF958RAN), NRF1 (ENCFF674LRE), NONO (ENCFF984PYA), GTF2F1 (ENCFF698GEX), PRPF4 (ENCFF103MEZ), SP1 (ENCFF593LCA), NELFE (ENCFF000YUF), SAFB (ENCFF908KFH), SRSF1 (ENCFF975FFH), RBM15 (ENCFF402MOS), SIN3A (ENCFF154ZWB), MYC (ENCFF000YKV), BCLAF1(ENCFF144FIS), HNRNPLL(ENCFF768SVS), SETDB1 (ENCFF247BJO), RBM14 (ENCFF957SXO), HNRNPH1 (ENCFF594IKC), YY1 (ENCFF193QAR), ZHX1 (ENCFF899GZH), TARDBP (ENCFF216EBS) and TAF15 (ENCFF818HIH)).
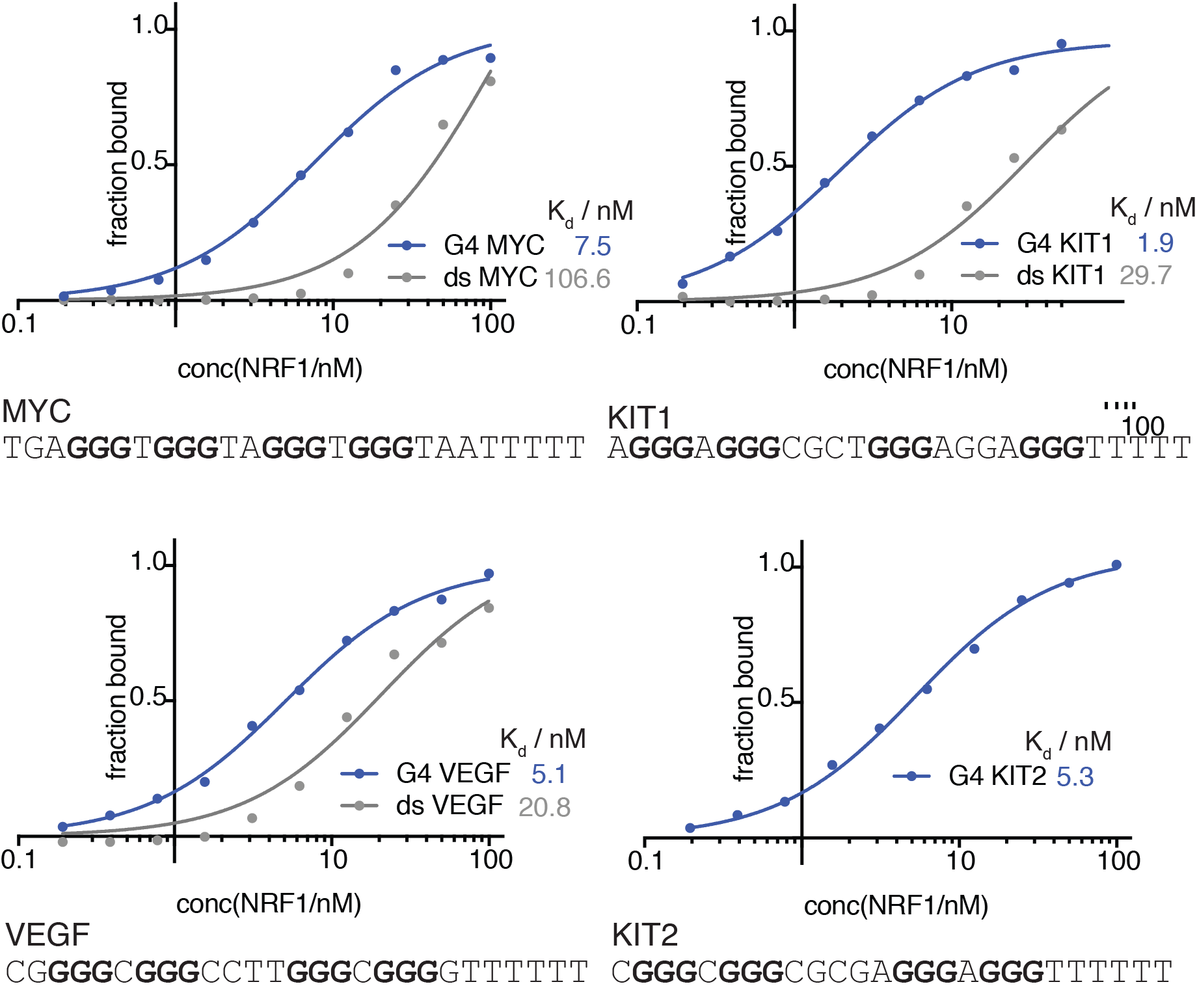


**Fig. S9.** Structural specificity of TF-G4 interactions.

Binding curves as determined by ELISA show high-affinity recombinant FLAG-NRF1 binding to different G4 structures and selectivity over respective double-stranded (N = 1).


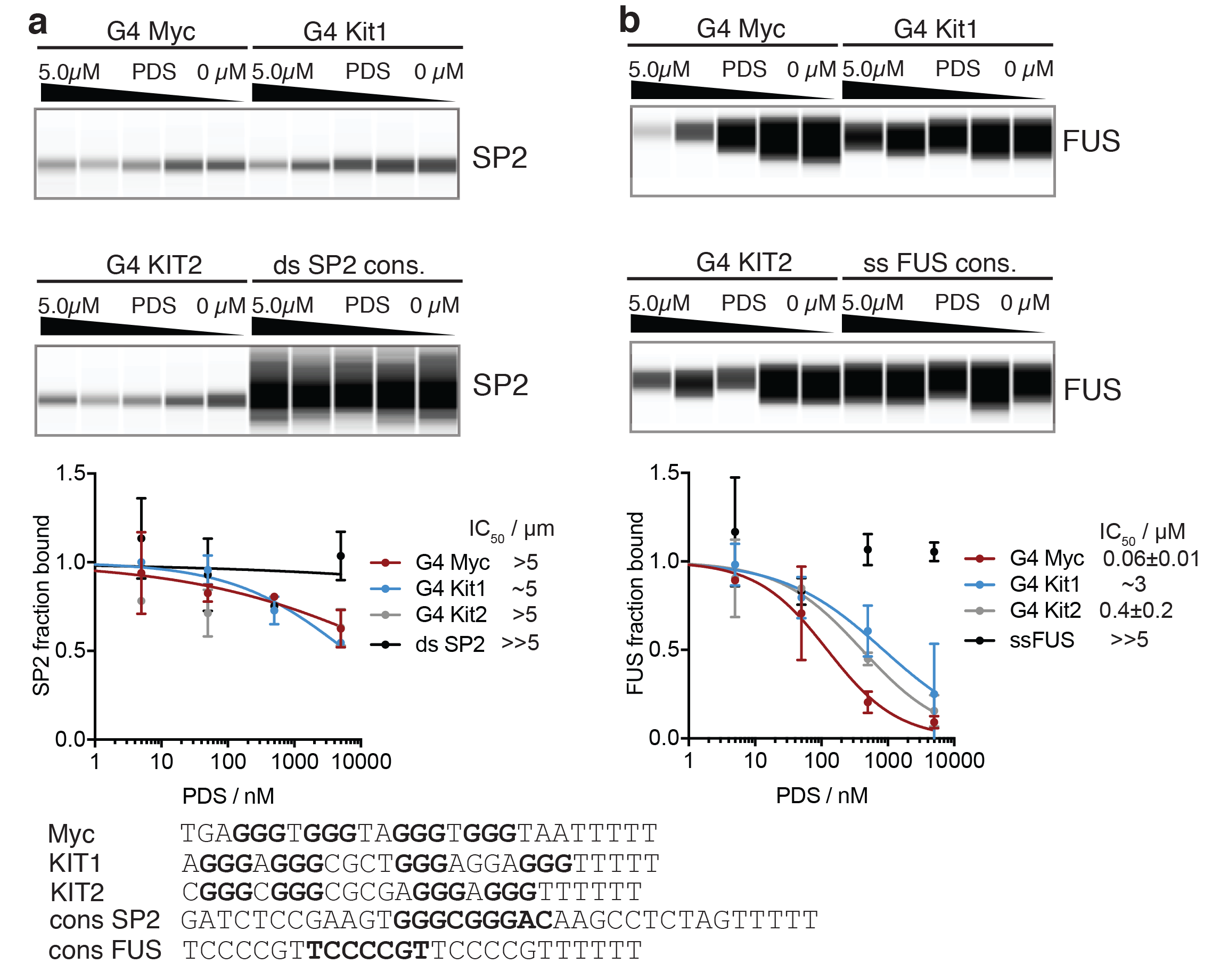


**Fig. S10.** G4 ligands compete with TFs for binding to G4 structures.

PDS dose-dependent competition for different G4-associated TFs (**a**) SP2 and (**b**) FUS from K562 cell nuclear lysate. PDS shows varying degrees of displacement of both TF from different G4 structures. However, PDS does not interfere with binding to the respective consensus oligomer (error bars display standard deviation, N = 2).


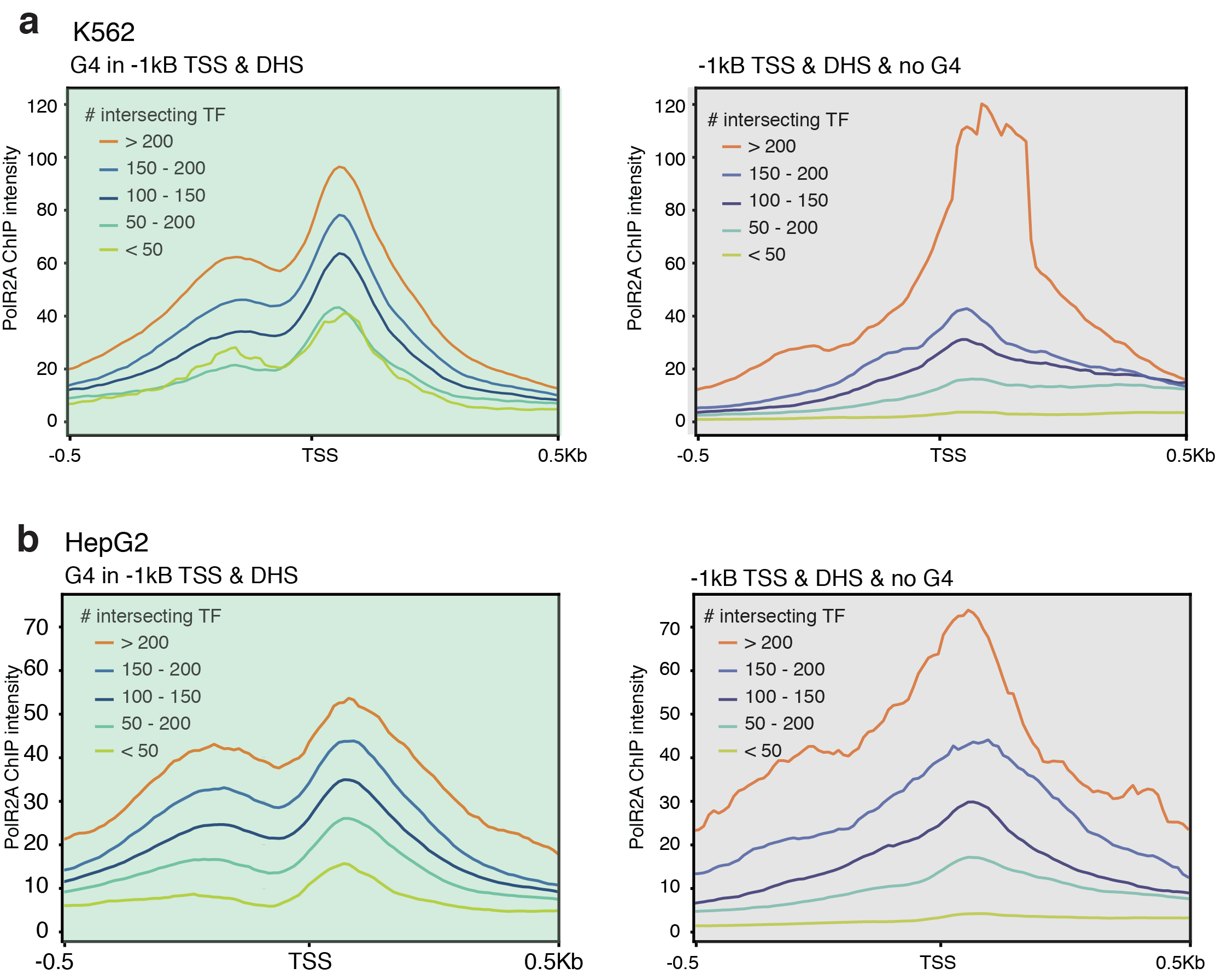


**Fig. S11.** RNA Polymerase 2 occupancy depends on TF occupancy, but not on G4s.

PolR2A ChIP-seq signal (ENCODE ENCFF000YWV) distribution around TSS depending on occupancy categories in K562 (**a**) and HepG2 (**b**). Sites marked by endogenous G4 in promoters accessible in open chromatin (-1kb upstream TSS, DHS positive) are coloured in green whereas promoters lacking an endogenous G4 are represented in grey.

Table S1. DNA oligonucleotides used in this study.

| Oligomer | Sequence (sense 5’  3’) | PDB | Ref |
| --- | --- | --- | --- |
| G4 Myc | TGA**GGG**T**GGG**TA**GGG**T**GGG**TAATTTTT [BtnTg] | 1XAV | [4] |
| G4 KIT1 | A**GGG**A**GGG**CGCT**GGG**AGGA**GGG**TTTTT [BtnTg] | 4WO3 | [5] |
| G4 KIT2 | C**GGG**C**GGG**CGCGA**GGG**A**GGG**TTTTTT [BtnTg] | 2KYP | [6] |
| G4 VEGFA | CG**GGG**C**GGG**CCTT**GGG**C**GGG**GTTTTTT [BtnTg] | 2M27 | [7] |
| ss mutMyc | TGA**GTG**T**GTG**TA**GTG**T**GTG**TAATTTTT [BtnTg] | - | - |
| ss Myc* | [Biosg] TTTTTTGA**GZG**T**GZG**TA**GZG**T**GZG**TA | - | - |
| ds Myc | TGA**GGG**T**GGG**TA**GGG**T**GGG**TAATTTTT [BtnTg] | - | - |
|  | TTACCCACCCTACCCACCCTCA |  |  |
| ds mutMyc | TGA**GTG**T**GTG**TA**GTG**T**GTG**TAATTTTT [BtnTg] | - | - |
|  | TTACACACACTACACACACTCA |  |  |
| ds KIT1 | A**GGG**A**GGG**CGCT**GGG**AGGA**GGG**TTTTT [BtnTg] | - | - |
|  | CCCTCCTCCCAGCGCCCTCCCT |  |  |
| ds consensus. NRF1 | GATCCGGCTCCTGCGCATGCGTGCTGCTATTTTT [BtnTg] | - | [8] |
|  | TAGCAGCACGCATGCGCAGGAGCCGGATC |  |  |
| ds consensus Sp2 | GATCTCCGAAGTGGGCGGGACAAGCCTCTAGTTTTT [BtnTg] | - | [9] |
|  | CTAGAGGCTTGTCCCGCCCACTTCGGAGATC |  |  |
| ss consensus FUS | TCCCCGTTCCCCGTTCCCCGTTTTTT [BtnTg] | - | [10] |
| ds consensus MYC | GAGCACGTGGCTTTTT [BtnTg] |  | [2] ^a^ |
|  | GCCACGTGCTC |  |  |
| ds consensus YY1 | CCAAATGGCGGCCTTTTT [BtnTg] |  | [2] ^a^ |
|  | GGCCGCCATTTGG |  |  |
| ds consensus ZHX1 | TTACGTAATTTTT [BtnTg] |  | [2]^a^ |
|  | TTACGTAA |  |  |

BtnTg = biotin tetraethylene glycol; Biosg = biotin amino-PEG1; Z = 8-aza-7-deazaguanosine; ^a^ Derived from reported high confidence PWM

**Table S2**. Western-blot quantification corresponding to Fig. 2a and S7*.*

| Target | Binding | Area [% total lysate]^b^ | | | | | | |
| --- | --- | --- | --- | --- | --- | --- | --- | --- |
|  | **Properties^a^** | beads | G4  Myc | ds  Myc | ss  mut Myc | ds  mut Myc | G4  Kit 1 | ds  Kit1 |
| SP2 | G4 | 0.01±0.00 | 1.00±0.02 | 0.01±0.01 | 0.02±0.01 | 0.04±0.01 | 0.95±0.46 | 0.01±0.01 |
| E2F4 | no binding | <0.05 | <0.05 | <0.05 | <0.05 | <0.05 | <0.05 | <0.05 |
| FUS | G4 | 0.01±0.00 | 19.74±0.79 | 0.09±0.06 | 8.30±0.14 | 0.00±0.01 | 10.42±1.09 | 0.00±0.00 |
| NRF1 | G4 | 0.37±0.02 | 1.00±0.12 | 0.23±0.08 | 0.34±0.06 | 0.39±0.22 | 0.63±0.05 | 0.23±0.01 |
| CEBPZ | no binding | <0.05 | <0.05 | <0.05 | <0.05 | <0.05 | <0.05 | <0.05 |
| NONO | non-selective | 0.13±0.07 | 7.03±3.00 | 0.10±0.04 | 9.71±6.55 | 0.08±0.01 | 0.60±0.14 | 0.11±0.05 |
| U2AF1 | no binding | <0.05 | <0.05 | <0.05 | <0.05 | <0.05 | <0.05 | <0.05 |
| ATF1 | no binding | <0.05 | <0.05 | <0.05 | <0.05 | <0.05 | <0.05 | <0.05 |
| GTF2F1 | G4 | 0.3±0.3 | 4.0±2.4 | 0.1±0.1 | 0.7±0.3 | n.d. | 5.1±1.2 | n.d. |
| THAP1 | no binding | <0.05 | <0.05 | <0.05 | <0.05 | n.d. | <0.05 | n.d. |
| PRPF4 | G4 | 0.7±0.0 | 8.8±0.3 | 0.6±0.4 | 2.4±0.5 | n.d. | 7.2±0.0 | n.d. |
| SP1 | G4 | 0.2±0.0 | 1.2±0.3 | 0.2±0.1 | 0.3±0.1 | n.d. | 0.8±0.0 | n.d. |
| NELFE | G4 | 0.1±0.0 | 10.1±5.0 | 0.2±0.1 | 0.3±0.1 | n.d. | 6.1±2.0 | n.d. |
| SAFB | G4 | 0.0±0.0 | 5.5±3.7 | 0.0±0.0 | 1.5±0.0 | n.d. | 4.8±0.1 | n.d. |
| NFYA | no binding | <0.05 | <0.05 | <0.05 | <0.05 | <0.05 | <0.05 | <0.05 |
| HNRNPL | no binding | <0.05 | <0.05 | <0.05 | <0.05 | n.d. | <0.05 | n.d. |
| IRF1 | no binding | <0.05 | <0.05 | <0.05 | <0.05 | n.d. | <0.05 | n.d. |
| SRSF1 | G4 | 0.1±0.1 | 1.9±0.3 | 0.7±0.5 | 1.2±0.4 | n.d. | 6.0±1.1 | n.d. |
| RBM15 | G4 | 0.0±0.0 | 11.8±15.2 | 0.1±0.0 | 3.5±3.1 | n.d. | 0.7±0.1 | n.d. |
| ELK1 | no binding | <0.05 | <0.05 | <0.05 | <0.05 | n.d. | <0.05 | n.d. |
| SIN3A | G4 | 1.3±1.5 | 3.1±2.2 | 0.3±0.1 | 0.3±0.5 | n.d. | 3.2±4.6 | n.d. |
| MYC | G4 | 0.7±0.0 | 1.4±0.3 | 0.3±0.1 | 0.7±0.4 | n.d. | 1.5±0.5 | n.d. |
| BCLAF1 | G4 | 0.0±0.0 | 25.3±23.1 | 0.1±0.0 | 0.9±0.2 | n.d. | 24.4±2.6 | n.d. |
| HNRNPLL | G4 | 4.5±2.8 | 3.1±1.8 | 1.2±1.7 | 2.3±1.6 | n.d. | 13.1±12.7 | n.d. |
| SETDB1 | non-selective | 0.6±0.9 | 4.6±0.8 | 5.0±5.8 | 1.6±0.5 | n.d. | 3.2±0.2 | n.d. |
| RBM14 | G4 | 0.1±0.0 | 0.8±0.1 | 0.1±0.0 | 0.3±0.1 | n.d. | 0.9±0.2 | n.d. |
| DEAF1 | no binding | <0.05 | <0.05 | <0.05 | <0.05 | n.d. | <0.05 | n.d. |
| HNRNPH1 | non-selective | 0.9±0.1 | 4.4±0.1 | 2.7±0.5 | 1.7±0.1 | n.d. | 3.0±0.4 | n.d. |
| YY1 | G4 | 0.4±0.1 | 2.1±0.6 | 0.3±0.0 | 0.4±0.1 | n.d. | 1.6±0.3 | n.d. |
| RFX5 | no binding | <0.05 | <0.05 | <0.05 | <0.05 | n.d. | <0.05 | n.d. |
| ZHX1 | G4 | 0.2±0.0 | 3.0±1.2 | 0.2±0.1 | 0.4±0.2 | n.d. | 1.7±0.5 | n.d. |
| TARDBP | ssDNA | 0.9±0.1 | 4.6±0.7 | 3.3±1.1 | 61.9±11.7 | n.d. | 2.9±0.0 | n.d. |
| TAF15 | G4 | 0.7±0.3 | 59.0±25.9 | 1.9±0.6 | 15.5±5.7 | n.d. | 26.6±4.7 | n.d. |
| FOXA1 | no binding | <0.05 | <0.05 | <0.05 | <0.05 | <0.05 | <0.05 | <0.05 |
| CTCF | no binding | <0.05 | <0.05 | <0.05 | <0.05 | <0.05 | <0.05 | <0.05 |

**^a^ “**G4”: G4-binding >2-fold over beads and any of the controls; “non-selective”: G4-binding >2-fold over beads, but not controls; “ssDNA”: ssDNA-binding >2-fold over any G4; “no binding”: binding not detected to any of the baits

**^b^** Area normalized to loading control. Error bars display standard deviation, N =2.

**Table S3**. Western-blot quantification corresponding to Fig. 2b*.*

|  | Area [% total lysate]^b^ | | | |
| --- | --- | --- | --- | --- |
|  | G4  Myc | ss  mut Myc | ss  Myc* | consensus |
| SP2 | 1.7±0.7 | 0.4±0.1 | 0.3±0.2 | 3.7±0.8 |
| FUS | 56.3±11.5 | 16.0±6.1 | 0.8±0.7 | 32.4±3.8 |
| NRF1 | 1.3±0.0 | 0.6±0.2 | 0.6±0.2 | 2.0±0.0 |
| MYC | 13.0±2.1 | 5.4±0.4 | 5.0±1.0 | 19.7±2.2 |
| YY1 | 1.8±2.4 | 0.7±1.3 | 0.6±1.0 | 12.8±11.7 |
| ZHX | 15.9±0.3 | 4.5±0.0 | 4.7±0.5 | 8.4±0.1 |

**^a^** Area normalized to loading control. Error bars display standard deviation, N =2.

**Table S4**. Antibodies used in this study.

| # | Target | Species | Type | Company | Cat. no. |
| --- | --- | --- | --- | --- | --- |
| 1 | SP2 | rabbit | poly | Abcam | ab229468 |
| 2 | E2F4 | goat | poly | Bio-techne | AF5139 |
| 3 | FUS | mouse | mono | Santa-cruz | sc-47711 |
| 4 | NRF1 | mouse | mono | CDI | R157.1.3D4 |
| 5 | CEBPZ | rabbit | poly | Sigma | AV100660 |
| 6 | NONO | mouse | mono | Santa-cruz | sc-166702 |
| 7 | U2AF1 | rabbit | mono | Proteintech | 10334-1-AP |
| 8 | ATF1 | rabbit | poly | Genetex | GTX129120 |
| 9 | GTF2F1 | rabbit | mono | Proteintech | 10093-2-AP |
| 10 | THAP1 | rabbit | mono | Proteintech | 12584-1-AP |
| 11 | SP1 | rabbit | mono | Proteintech | 21962-1-AP |
| 12 | NELFE | rabbit | mono | Proteintech | 10705-1-AP |
| 13 | SAFB | rabbit | mono | Proteintech | 21857-1-AP |
| 14 | NFYA | rabbit | poly | Abcam | ab6558 |
| 15 | HNRNPL | rabbit | mono | Proteintech | 18354-1-AP |
| 16 | IRF1 | rabbit | mono | Proteintech | 11335-1-AP |
| 17 | SRSF1 | rabbit | mono | Proteintech | 12929-2-AP |
| 18 | RBM15 | rabbit | mono | Proteintech | 10587-1-AP |
| 19 | ELK1 | rabbit | mono | Proteintech | 27420-1-AP |
| 20 | SIN3A | rabbit | mono | Proteintech | 14638-1-AP |
| 21 | MYC | rabbit | mono | Cell Signaling | D84C12 |
| 22 | BCLAF1 | rabbit | mono | Proteintech | 26809-1-AP |
| 23 | HNRNPLL | rabbit | mono | Proteintech | 26769-1-AP |
| 24 | SETDB1 | rabbit | mono | Proteintech | 11231-1-AP |
| 25 | RBM14 | rabbit | mono | Proteintech | 10196-1-AP |
| 26 | DEAF1 | rabbit | mono | Proteintech | 18323-1-AP |
| 27 | HNRNPH1 | rabbit | mono | Proteintech | 14774-1-AP |
| 28 | YY1 | rabbit | mono | Proteintech | 22156-1-AP |
| 29 | RFX5 | rabbit | mono | Proteintech | 12137-1-AP |
| 30 | ZHX1 | rabbit | mono | Proteintech | 13903-1-AP |
| 31 | TARDBP | rabbit | mono | Proteintech | 10782-2-AP |
| 32 | TAF15 | rabbit | mono | Proteintech | 25521-1-AP |
| 33 | FOXA1 | rabbit | poly | Abcam | ab23738 |
| 34 | CTCF | rabbit | poly | Merck Millipore | 07-729 |
| 35 | FLAG-tag^1^ | goat | poly | Abcam | ab1238 |
| 36 | G4s^2^ | ScFv^2^ | mono^2^ | -^2^ | -^2^ |

^1^ horseradish peroxidase (HRP)-conjugated antibody

^2^ custom G4-structure-specific single-chain antibody BG4 was prepared in BL21(DE3) E. coli using the expression vector pSANG10-3F-BG4 (Addgene, plasmid #55756) (Nat. Chem. 5, 182–6 (2013))

**Table S5**. qPCR control regions for TF native ChIP experiments.

| **Region** | **coordinates** | **Sequence of primer pairs (5’-3’)** | **G4 ChIP** | **TF** |
| --- | --- | --- | --- | --- |
| *PRC1* | chr15:91,537,740-91,538,106 | CAATCGGGGTGGGGACTCG  CACAGACAGTGACGTCATCCCC | **+** | **SP2, NRF1, FUS** |
| *SDF4* | chr1:1,167,340-1,167,697 | GCGCCACCATCTCTCCTC  CTCGGGCACCCTTGTCATT | **+** | **SP2, FUS** |
| *RBBP4* | chr1:33,116,658-33,116,826 | GAAAGCTACTCCGCGCGTCT  ACCTTCGCGCCAACATCAG | **+** | **SP2, NRF1, FUS** |
| *SIRT4* | chr12:120,755,329-120,755,549 | GGCCGCCATTCACCAATAGA  CCGCCACAGGATGTGATTGT | **+** | **SP2, NRF1** |
| *RPA3* | chr7:7,680,284-7,680,365 | CGGAAGTTGACAGATACAGGG  GATCGCAGAAAGGTAGTCTCAG | **+** | **SP2, NRF1** |
| *MAZ* | chr16:29,816,814-29,816,903 | ACTCAGCGCAGGATTGTAAATA  CCTCATGCTTCGGCTTCC | **+** | **NRF1, FUS** |
| *JS048* | chr1:6,101,625-6,101,752 | GAAACTGGCTCCGGGTCTCT  CTCAGGGCAGTCTCTGAGGAA | **-** | **CTCF** |
| *JS051* | chr3:140,911,187-140,911,419 | GATTTGGGGACCACAGGCTC  GCTAACTAGCAGCAGTCACCA | **-** | **CTCF** |
| *JS053* | chr17:32,688,573-32,688,804 | AAGCTCAGCGCTCCCTCTA  GATCACCTGCTCAATCTCTCCC | **-** | **CTCF** |
| *JS055* | chr1:28,306,102-28,306,409 | GATGACTCAATCCTCCCTCCAC  AGACCCTATTGGGGTTGTGTG | **-** | **FOXA1** |
| *JS057* | chr1:28,306,102-28,306,409 | TGCTTCTGCCACCTTCTTCAG  GTCACTCTGTTGGGCAAGGG | **-** | **FOXA1** |
| *JS059* | chr1:29,253,501-29,253,672 | ACAGCAAGTCTGGCTTCAGAG  AACACCCTCCCAACTCAAACC | **-** | **FOXA1** |
| *JS062* | chr3:38,028,327-38,028,871 | GGGGGATGAAGCCTGGGATA  TGAAGGAGTGGCCCCAAGAT | **-** | **FOXA1** |
| *TMCC1* | chr3:129,392,843-129,392,937 | GTGGTACACTGCCTACAGTATT  GTATAACGCCTGGGCTATGT | **-** | **-** |
| *ESR1* | chr6:151,937,355-151,937,453 | GAAACAGCCCCAAATCTCAA  TTGTAGCCAGCAAGCAAATG | **-** | **-** |

**SUPPLEMENTAL REFERENCES**

1. Chambers VS, Marsico G, Boutell JM, Di Antonio M, Smith GP, Balasubramanian S. High-throughput sequencing of DNA G-quadruplex structures in the human genome. Nat Biotechnol. 2015;33:877–81.

2. Lambert SA, Jolma A, Campitelli LF, Das PK, Yin Y, Albu M, et al. The Human Transcription Factors. Cell.; 2018;172:650–65.

3. Kypr J, Kejnovská I, Renčiuk D, Vorlíčková M. Circular dichroism and conformational polymorphism of DNA. Nucleic Acids Res. 2009;37:1713–25.

4. Ambrus A, Chen D, Dai J, Jones RA, Yang D. Solution structure of the biologically relevant G-quadruplex element in the human c-MYC promoter. Implications for G-quadruplex stabilization. Biochemistry. 2005;44:2048–58.

5. Wei D, Parkinson GN, Reszka AP, Neidle S. Crystal structure of a c-kit promoter quadruplex reveals the structural role of metal ions and water molecules in maintaining loop conformation. Nucleic Acids Res. 2012;40:4691–700.

6. Kuryavyi V, Phan AT, Patel DJ. Solution structures of all parallel-stranded monomeric and dimeric G-quadruplex scaffolds of the human c-kit2 promoter. Nucleic Acids Res. 2010;38:6757–73.

7. Agrawal P, Hatzakis E, Guo K, Carver M, Yang D. Solution structure of the major G-quadruplex formed in the human VEGF promoter in K+: insights into loop interactions of the parallel G-quadruplexes. Nucleic Acids Res. 2013;41:10584–92.

8. Chau CM, Evans MJ, Scarpulla RC. Nuclear respiratory factor 1 activation sites in genes encoding the gamma-subunit of ATP synthase, eukaryotic initiation factor 2 alpha, and tyrosine aminotransferase. Specific interaction of purified NRF-1 with multiple target genes. J Biol Chem. 1992;267:6999–7006.

9. Terrados G, Finkernagel F, Stielow B, Sadic D, Neubert J, Herdt O, et al. Genome-wide localization and expression profiling establish Sp2 as a sequence-specific transcription factor regulating vitally important genes. Nucleic Acids Res. 2012;40:7844–57.

10. Tan AY, Riley TR, Coady T, Bussemaker HJ, Manley JL. TLS/FUS (translocated in liposarcoma/fused in sarcoma) regulates target gene transcription via single-stranded DNA response elements. Proc Natl Acad Sci. 2012;109:6030–5.
