# Supplementary material for: G-quadruplexes are transcription factor binding hubs in human chromatin
Source: Genome Biol. 2021 Apr 23;22:117. doi: 10.1186/s13059-021-02324-z (PMC8063395; doi:10.1186/s13059-021-02324-z)
Supplement: Supplementary file 7 — Additional file 7. Supplemental Information (Supplemental Methods; Supplemental Data Analysis; Supplemental Discussion.). [file 13059_2021_2324_MOESM7_ESM.docx]

**Supplemental Information for**

G-quadruplexes are transcription factor binding hubs in human chromatin

Jochen Spiegel^1^, Sergio Martínez Cuesta^1,2^, Santosh Adhikari^2^, Robert Hänsel-Hertsch^1,4^, David Tannahill^1^, Shankar Balasubramanian^1,2,3^*

^1^Cancer Research UK Cambridge Institute, Li Ka Shing Centre, Robinson Way, Cambridge, CB2 0RE, UK.

^2^Department of Chemistry, University of Cambridge, Cambridge, CB2 1EW, UK

^3^School of Clinical Medicine, University of Cambridge, Cambridge, CB2 0SP, UK

^4^Current address: Center for Molecular Medicine Cologne, University of Cologne, 50931 Cologne, Germany

*Correspondence: [sb10031@cam.ac.uk](mailto:sb10031@cam.ac.uk) (S.B.)

**This PDF file includes:**

Supplemental Methods

Supplemental Data analysis

Supplemental Discussion

**Supplemental METHODS**

**Oligonucleotide folding**

Oligonucleotides were PAGE purification quality obtained from Sigma and Integrated DNA Technologies. For G4 formation, 10 µM DNA oligonucleotide was annealed in 10 mM Tris HCl, pH 7.4, 100 mM KCl by heating at 95 °C for 5 min followed by gradually cooling to 20°C. For double-stranded DNA, 10 µM forward and reverse strand oligonucleotides were mixed and annealed in 10 mM Tris HCl, pH 7.4, 100 mM NaCl in the same manner.

**Circular dichroism spectroscopy**

Circular dichroism spectra were recorded on an Applied Photo-physics Chirascan circular dichroism spectropolarimeter using a 1 mm path length quartz cuvette. Circular dichroism measurements were performed at 298 K over a range of 200 – 330 nm using a response time of 0.5 s, 1 nm pitch, and 1 nm bandwidth. The recorded spectra represent a smoothed average of three scans, zero-corrected at 330 nm (molar ellipticity θ is quoted in 105° cm^2^ dmol^−1^). The buffer absorbance was subtracted from the recorded spectra. The data was matched against reference spectra from previous full structural characterizations (see Table S1).

**Enzyme-linked immunosorbent assay**

ELISAs for binding affinity and specificity were performed as described previously[1] with minor modifications. Briefly, biotinylated oligonucleotides were bound to Pierce Streptavidin Coated High Capacity Plates (ThermoFisher) followed by blocking with 3% BSA and incubation with recombinant FLAG-tagged full-length recombinant protein for human nuclear respiratory factor 1 (NRF1), transcript variant 2, (Insight Biotechnology, cat. no. TP317852) in ELISA buffer (25 mM HEPES, 10.5 mM, 110mM KCl, 1 mM MgCl_2_, 10% glycerol, 0.01% Igepal C-630, 1 mM DDT). After three washes with ELISA buffer, detection was achieved with an anti-FLAG horseradish peroxidase (HRP)-conjugated antibody (ab1238, Abcam) and 3,3′,5,5′-tetramethylbenzidine (TMB) ELISA substrate (Slow Kinetic Rate, ab171525, Abcam). Signal intensity was measured at 450 nm on a PHERAstar microplate reader (BMG Labtech). Dissociation constants (*K*_d_) and half maximal inhibitory concentrations (IC_50_) were calculated from binding curves assuming using Prism (GraphPad Software Inc.). Standard error of means (s.e.m.) were calculated from three replicates.

**Supplemental Data analysis.**

Capillary-based immunoassays on a Wes Protein Simple Western System were analysed using Compass for SW (4.1) (Protein Simple). qPCR data ELISA data and PDS dose-dependent competition experiments were processed with Prism 7 (GraphPad Software Inc.). Boxplots were generated ggplot2 v3.2.1 in R; all other graphs were plotted using either Prism 7 (Graphpad), or visualized with R. In the box plots, the box limits are the first and third quartiles respectively and the whiskers are the 1.5x interquartile range. The center line within the box represents the median.

**Bioinformatics tools and scripts.**

Bioinformatics data analyses and processing were performed using Bash, R and Python programming languages. The following tools were also used: cutadapt (v 1.16) [2], BWA (v 0.7.15)[3], Picard (v 2.14.0; http://broadinstitute.github.io/picard), MACS2 (v 2.1.1) [4], bedtools (v 2.26.0; http://bedtools.readthedocs.io/en/latest/content/overview.html), samtools (v 1.6) [5], deepTools (v 3.1.2) [6], Intervene(0.6.4), GAT (v 1.3.5)[7] and FIMO (v4.11.2) [8]. All scripts are available at https://github.com/sblab-bioinformatics/G4-vs-TFs.

**Mapping, peak calling and peak processing.**

Raw fastq reads from G4 or native TF ChIP-seq were trimmed with cutadapt[2] to remove adapter sequences and low-quality reads (mapping quality < 10). Reads were aligned to the human genome (version hg19) with BWA[3] and duplicates were marked using Picard (v 2.14.0; <http://broadinstitute.github.io/picard>) and removed using samtools[5]. G4 ChIP and TF ChIP peaks were called by MACS2[4] (q-value < 0.05) as previously outlined (https://github.com/sblab-bioinformatics/dna-secondary-struct-chrom-lands/blob/master/Methods.md). Peak overlaps in different replicates were visualized with Intervene[9]. Peaks were merged from replicates with bedtools multiinter. High confidence peaks were defined as those overlapping in five out of eight replicates (G4 ChIP-seq) in K562 cells or six out of nine replicates in HepG2 cells.

**Functional annotation of G4 ChIP–seq peaks and enrichment analysis.**

Datasets for DHS in K562 (ENCSR000EPC) and HepG2 (ENCFF571RHF) as well as potential G4 forming sequences[10] (potassium and PDS induced) (GSE63874) were downloaded from ENCODE and NCBI GEO, respectively, to calculate the overlap with G4 ChIP-seq high confidence peaks.

PAVIS[11] was used to annotate the G4 ChIP–seq peaks. The G4 ChIP–seq file was randomly shuffled across all potential G4s (command shuffleBed of the BEDTools package) three independent times and their genomic annotations retrieved via PAVIS. To calculate enrichments of annotated features, ratios of the annotated G4 ChIP–seq proportions were calculated against the proportions of the randomly shuffled features (N = 3).

**Generation control data set of potential G4 around TSS in K562.**

Datasets for DHSs (ENCSR000EPC) and potential G4 forming sequences[10] (GSE63874) were downloaded from ENCODE and GEO, respectively. To reflect the genomic features and location of endogenous G4 in the control data set, potential G4 forming sequences located in open chromatin (DHS) and not overlapping with endogenous G4 ChIP-seq peaks were annotated using PAVIS[11] (hg19, “known gene”) to select for sites located around TSS (1kb upstream TSS & 5’UTR). Motif analysis in G4 ChIP-seq high confidence peaks and control sites was performed using MEME-ChIP [12].

**Inference of strand information for endogenous G4s in K562.**

G4 ChIP does not provide stranded information. To infer positional information G4 ChIP-seq peaks were intersected with potential G4s[10] containing strand-specific information (bedtools intersect) and for cases where G4 forming potential could be attributed unambiguously to a single strand these were assigned to the endogenous G4 sites (2161 peaks exclusive to the plus and 2271 peaks exclusive to the minus strand).

**Statistical analysis of genomic association.**

Genomic binding sites for chromatin-associated factors and histone marks (aligned to hg19) were downloaded from ENCODE. All available ChIP-seq datasets were considered (524 data sets comprising 358 unique markers in K562 and 278 data sets comprising 241 unique markers in HepG2; see also Supplemental Data Table 1 and 2). To maximize the robustness of our analyses, where possible we selected ‘released’ over ‘archived’ data and generally chose ‘optimal idr’ over ‘conservative idr’ and ‘replicated’ peaks (a complete list of analyzed data sets can be found in Supplemental Data Table 1 and 2). Different ChIP-seq experiments targeting the same factor were treated independently. We used high confidence G4 ChIP-seq peaks described above. Randomization (10,000 times) of endogenous of G4s or control sites and statistical analysis was performed using GAT[7] in different workspaces (hg19, open chromatin, potential G4s[10] and potential G4s in open chromatin) in which ENCODE blacklisted regions had been excluded. Data sets with a q-value of < 0.05 and at least 100 peaks were considered. A list of proteins with known G4-association was derived from the G4-interacting protein data base (G4IPDB) [13].

For TFs that had been mapped in multiple different experiments, the maximum observed enrichment was considered, when comparing the enrichment in K562 and HepG2 cells.

**R loop association**

R-ChIP and DRIP-seq data mapping R-loops in K562 cells were downloaded from GEO (GSE97072) [14]. A set of overlapping peaks identified by R-ChIP and DRIP-seq were considered as R-loop confidence sites (GSE97072). The strandedness of endogenous G4s was derived as described above.

**TF consensus motif scanning and genomic association.**

TF consensus binding motifs were downloaded as position weight matrices (PWMs) from JASPAR2020[15] and scanned against the human genome using FIMO[8] with default parameters to generate maps of individual motif occurrences (available for 193 TFs that had been mapped in K562 cells representing 116 unique TFs). Genomic association testing was performed using GAT[7] by randomization of TF ChIP-seq sites (10,000 times) and statistical analysis for enrichment at predicted consensus binding sites and G4 ChIP-seq sites in either promoters (1kb upstream TSS). In addition, we performed the analysis restricted to promoters accessible in open chromatin to control for the strong overlap of endogenous G4s with open chromatin regions.

**Profile plots in genomic regions.**

Fragment coverage bigWig files were computed at 10 bp resolution, 200 bp average fragment size and normalization to sequencing depth (RPKM) using deepTools[6]. Signal distribution of ENCODE marks in K562 G4 ChIP-seq peaks and control sites was computed using plotProfile from deepTools [6].

*Expression analysis.* Gene expression levels quantified as transcripts per million (TPM) were downloaded from ENCODE for K562 (ENCSR000AEM, ENCSR545DKY) and HepG2 (ENCSR000CPE, ENCSR561FEE). GTF annotation files were downloaded from GENCODE v19 and promoters were defined as 1kb upstream the transcription start sites (TSS). Expression levels were compared for open chromatin promoters in which ENCODE markers intersected high confidence G4 ChIP-seq peaks (in 5 out of 8 replicates for K562 and 6 out of 9 replicates for HepG2) and open chromatin promoters, where ENCODE markers do not overlap with G4 ChIP-seq peaks (as in 2 out of 8 or 9 replicates).

In addition, promoters were classified depending on the number of binding TFs alone or the number of TFs intersecting a G4 ChIP-seq sites, respectively. Expression levels were compared for different occupancy categories ranging from <50 TFs up to >200 TFs.

**Supplemental Discussion.**

Example calculation for TF displacement in K562 nuclei by PDS

The average number of TF molecules per mammalian nucleus [25] is approximated to

$$N_{TF}={10}^{5}$$

Using a diameter of $d_{nucl}=6 \mu m$, the volume of a spherical K562 nucleus is approximated as

$$V_{nucl}=\frac{1}{6} \pi d_{nucl}^{3}=1.13\cdot{10}^{-13}L$$

The average number of TF molecules is converted to molar concentrations using the relationship:

$$c_{TF}=\frac{N_{TF}}{N_{A}\cdot V_{TF}}=1.5 \mu M$$

where *N_A_* is the Avogadro number ($6.0223\cdot{10}^{23}{mol}^{-1}$).

Based on previously described[26] biophysical experiments, the equilibrium dissociation constant of SP2 is estimated as $K_{d,SP2}=0.01 \mu M$

Using the Cheng-Prusoff[27] equation to approximate the equilibrium dissociation constant $K_{d,PDS}$ for PDS from the experimentally determined ${IC}_{50}=60\mu M$

$$K_{d,PDS}=\frac{{IC}_{50}}{\frac{c_{TF}}{K_{d,TF}}+1}=0.4\mu M$$

This corresponds closely to the sub-micromolar dissociation constants previously determined in biophysical experiments of PDS binding to different G4 oligomers [28].

**Supplemental References**

1. Biffi G, Tannahill D, McCafferty J, Balasubramanian S. Quantitative visualization of DNA G-quadruplex structures in human cells. Nat Chem. 2013;5:182–6.

2. Martin M. Cutadapt removes adapter sequences from high-throughput sequencing reads. EMBnet.journal. 2011;17:10.

3. Li H. Aligning sequence reads, clone sequences and assembly contigs with BWA-MEM. arXiv:13033997. 2013;

4. Zhang Y, Liu T, Meyer CA, Eeckhoute J, Johnson DS, Bernstein BE, et al. Model-based Analysis of ChIP-Seq (MACS). Genome Biol. 2008;9:R137.

5. Li H, Handsaker B, Wysoker A, Fennell T, Ruan J, Homer N, et al. The Sequence Alignment/Map format and SAMtools. Bioinformatics. 2009;25:2078–9.

6. Ramírez F, Ryan DP, Grüning B, Bhardwaj V, Kilpert F, Richter AS, et al. deepTools2: a next generation web server for deep-sequencing data analysis. Nucleic Acids Res. 2016;44:W160–5.

7. Heger A, Webber C, Goodson M, Ponting CP, Lunter G. GAT: A simulation framework for testing the association of genomic intervals. Bioinformatics. 2013;29:2046–8.

8. Grant CE, Bailey TL, Noble WS. FIMO: Scanning for occurrences of a given motif. Bioinformatics. 2011;27:1017–8.

9. Khan A, Mathelier A. Intervene: A tool for intersection and visualization of multiple gene or genomic region sets. BMC Bioinformatics. BMC Bioinformatics; 2017;18:1–8.

10. Chambers VS, Marsico G, Boutell JM, Di Antonio M, Smith GP, Balasubramanian S. High-throughput sequencing of DNA G-quadruplex structures in the human genome. Nat Biotechnol. Nature Publishing Group; 2015;33:877–81.

11. Huang W, Loganantharaj R, Schroeder B, Fargo D, Li L. PAVIS: A tool for Peak Annotation and Visualization. Bioinformatics. 2013;29:3097–9.

12. Machanick P, Bailey TL. MEME-ChIP: Motif analysis of large DNA datasets. Bioinformatics. 2011;27:1696–7.

13. Mishra SK, Tawani A, Mishra A, Kumar A. G4IPDB: A database for G-quadruplex structure forming nucleic acid interacting proteins. Sci Rep. Nature Publishing Group; 2016;6:38144.

14. Chen L, Chen JY, Zhang X, Gu Y, Xiao R, Shao C, et al. R-ChIP Using Inactive RNase H Reveals Dynamic Coupling of R-loops with Transcriptional Pausing at Gene Promoters. Mol Cell. Elsevier Inc.; 2017;68:745-757.e5.

15. Fornes O, Castro-Mondragon JA, Khan A, van der Lee R, Zhang X, Richmond PA, et al. JASPAR 2020: update of the open-access database of transcription factor binding profiles. Nucleic Acids Res. 2019;48:87–92.

16. Lambert SA, Jolma A, Campitelli LF, Das PK, Yin Y, Albu M, et al. The Human Transcription Factors. Cell. Elsevier Inc.; 2018;172:650–65.

17. Kypr J, Kejnovská I, Renčiuk D, Vorlíčková M. Circular dichroism and conformational polymorphism of DNA. Nucleic Acids Res. 2009;37:1713–25.

18. Ambrus A, Chen D, Dai J, Jones RA, Yang D. Solution structure of the biologically relevant G-quadruplex element in the human c-MYC promoter. Implications for G-quadruplex stabilization. Biochemistry. 2005;44:2048–58.

19. Wei D, Parkinson GN, Reszka AP, Neidle S. Crystal structure of a c-kit promoter quadruplex reveals the structural role of metal ions and water molecules in maintaining loop conformation. Nucleic Acids Res. 2012;40:4691–700.

20. Kuryavyi V, Phan AT, Patel DJ. Solution structures of all parallel-stranded monomeric and dimeric G-quadruplex scaffolds of the human c-kit2 promoter. Nucleic Acids Res. 2010;38:6757–73.

21. Agrawal P, Hatzakis E, Guo K, Carver M, Yang D. Solution structure of the major G-quadruplex formed in the human VEGF promoter in K+: insights into loop interactions of the parallel G-quadruplexes. Nucleic Acids Res. 2013;41:10584–92.

22. Chau CM, Evans MJ, Scarpulla RC. Nuclear respiratory factor 1 activation sites in genes encoding the gamma-subunit of ATP synthase, eukaryotic initiation factor 2 alpha, and tyrosine aminotransferase. Specific interaction of purified NRF-1 with multiple target genes. J Biol Chem. 1992;267:6999–7006.

23. Terrados G, Finkernagel F, Stielow B, Sadic D, Neubert J, Herdt O, et al. Genome-wide localization and expression profiling establish Sp2 as a sequence-specific transcription factor regulating vitally important genes. Nucleic Acids Res. 2012;40:7844–57.

24. Tan AY, Riley TR, Coady T, Bussemaker HJ, Manley JL. TLS/FUS (translocated in liposarcoma/fused in sarcoma) regulates target gene transcription via single-stranded DNA response elements. Proc Natl Acad Sci. 2012;109:6030–5.

25. Biggin MD. Animal Transcription Networks as Highly Connected, Quantitative Continua. Dev Cell. Elsevier Inc.; 2011;21:611–26.

26. Raiber EA, Kranaster R, Lam E, Nikan M, Balasubramanian S. A non-canonical DNA structure is a binding motif for the transcription factor SP1 in vitro. Nucleic Acids Res. 2012;40:1499–508.

27. Yung-Chi C, Prusoff WH. Relationship between the inhibition constant (KI) and the concentration of inhibitor which causes 50 per cent inhibition (I50) of an enzymatic reaction. Biochem Pharmacol. 1973;22:3099–108.

28. Le DD, Di Antonio M, Chan LKM, Balasubramanian S. G-quadruplex ligands exhibit differential G-tetrad selectivity. Chem Commun. 2015;51:8048–50.
