# Supplementary figures and images for: G-quadruplexes are transcription factor binding hubs in human chromatin
Source: Genome Biol. 2021 Apr 23;22:117. doi: 10.1186/s13059-021-02324-z (PMC8063395; doi:10.1186/s13059-021-02324-z)

Full scans of WES data presented in figures 2, 3, S7 and S9:

**
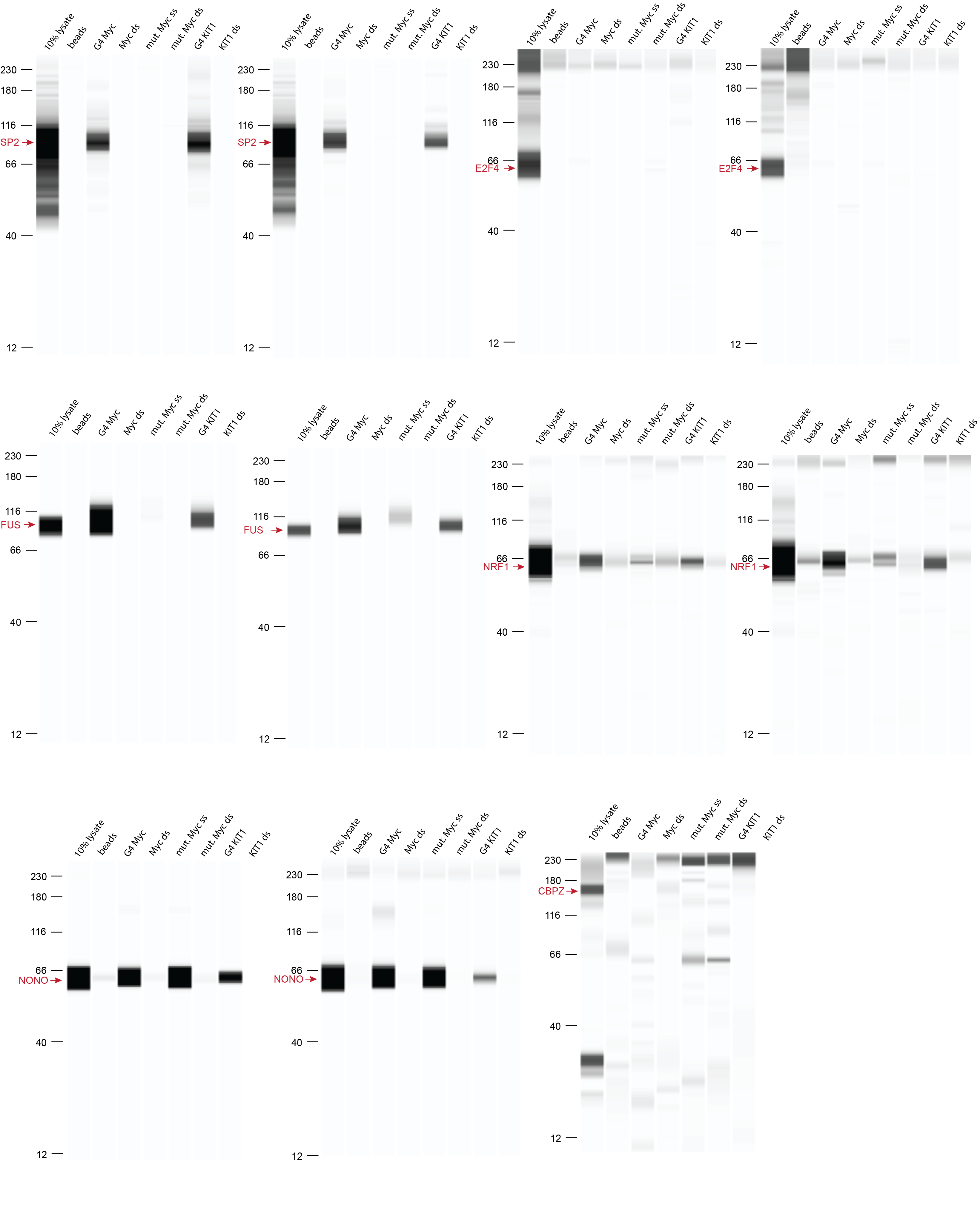
**

**
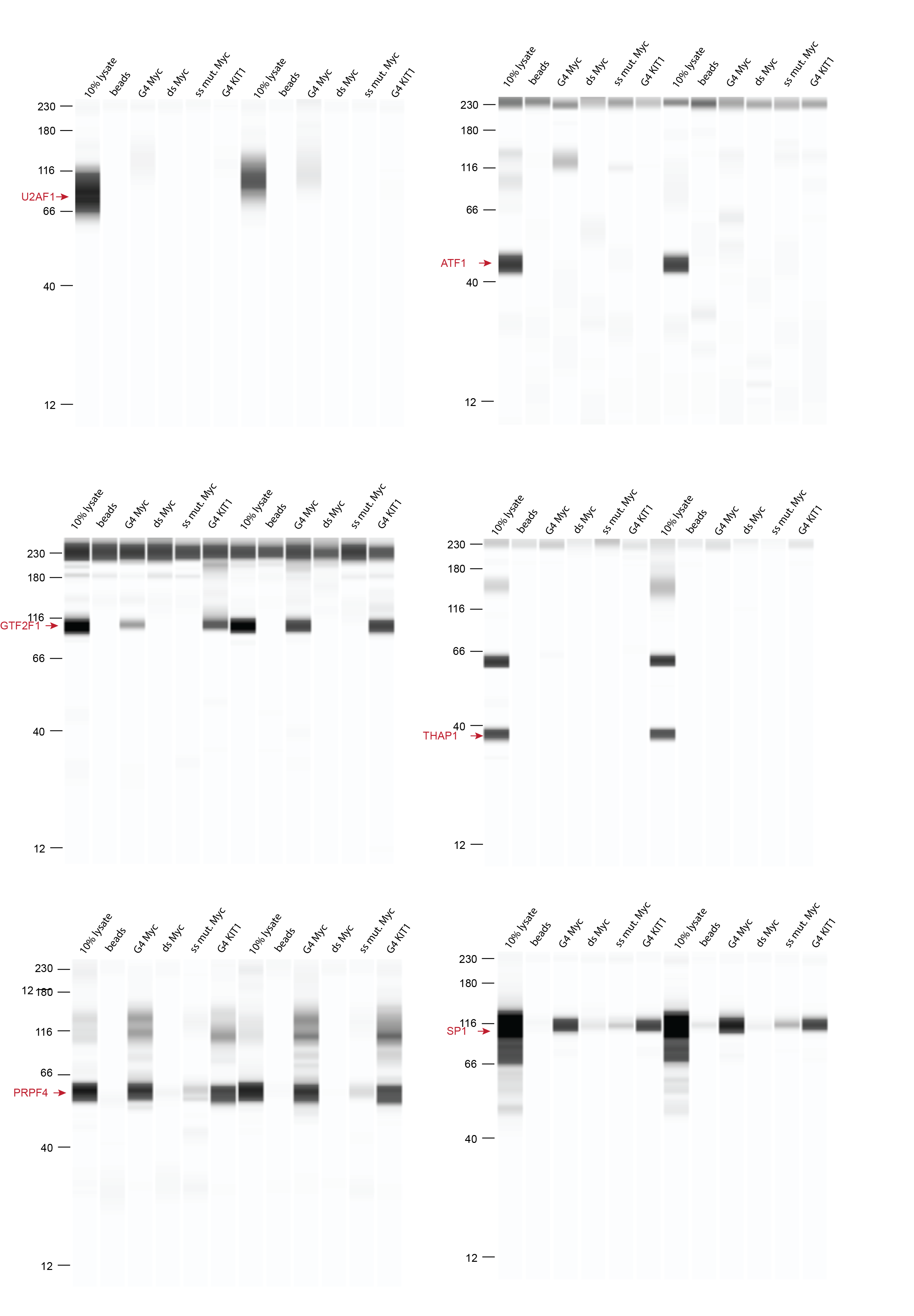
**

**
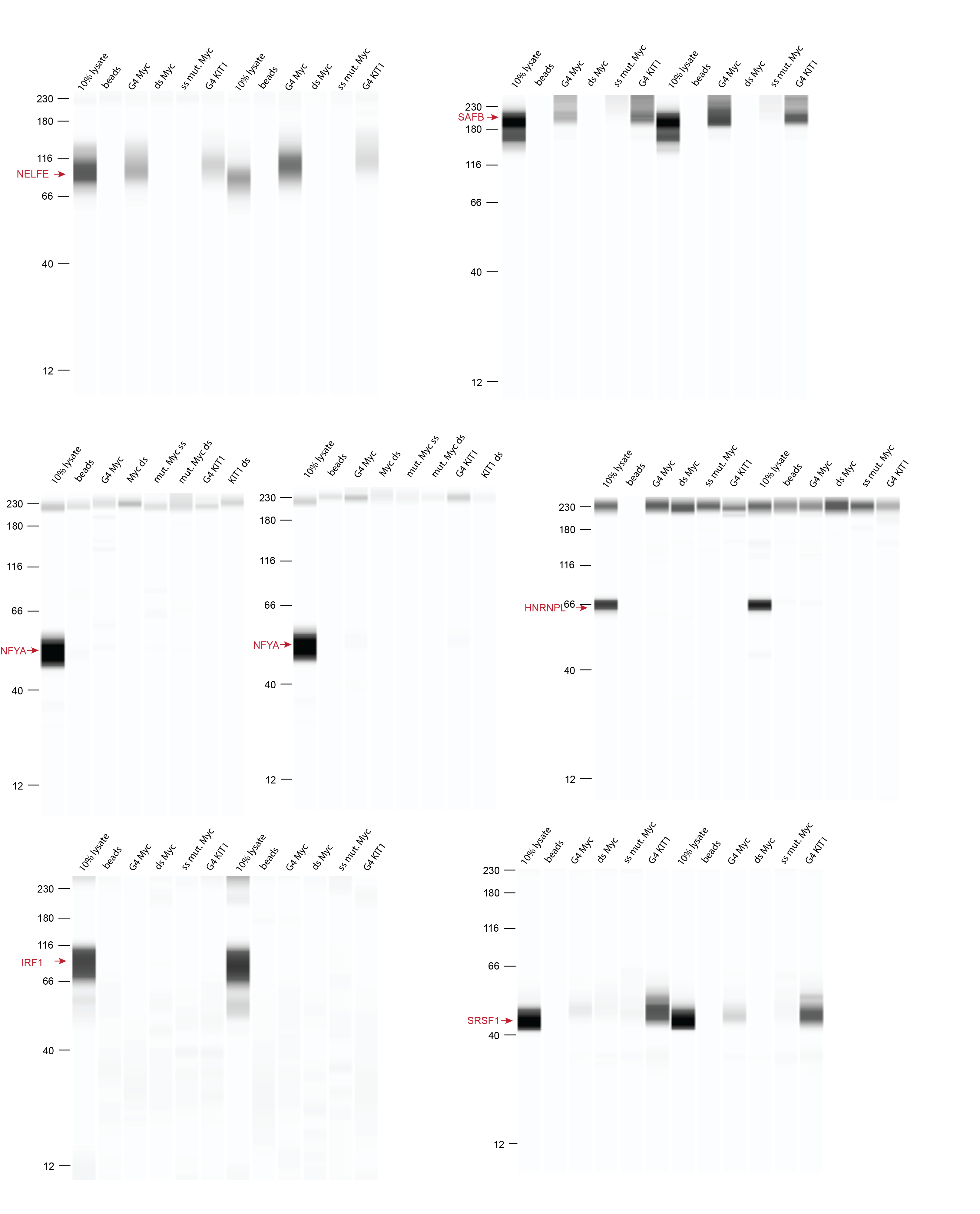
**

**
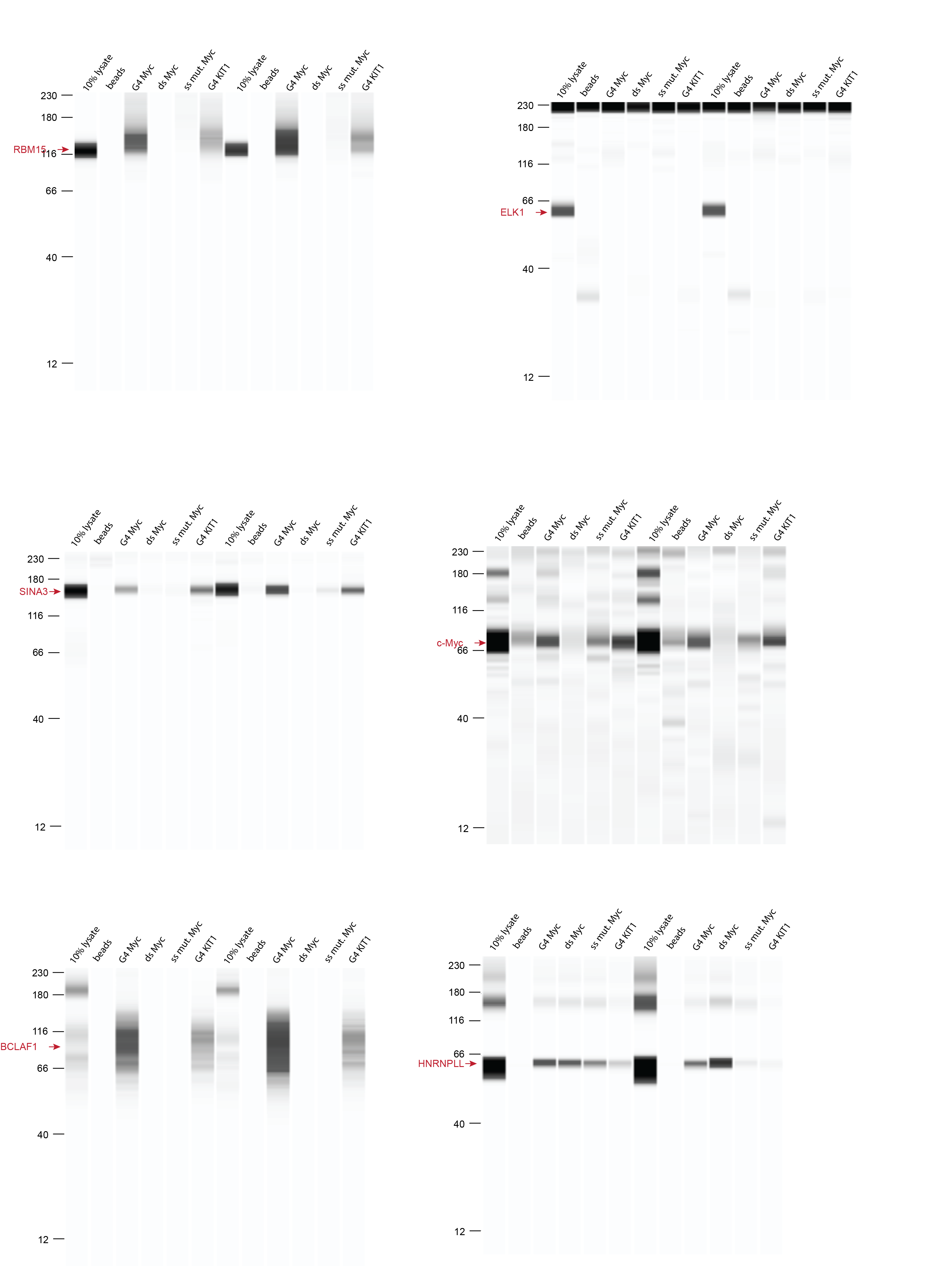
**

**
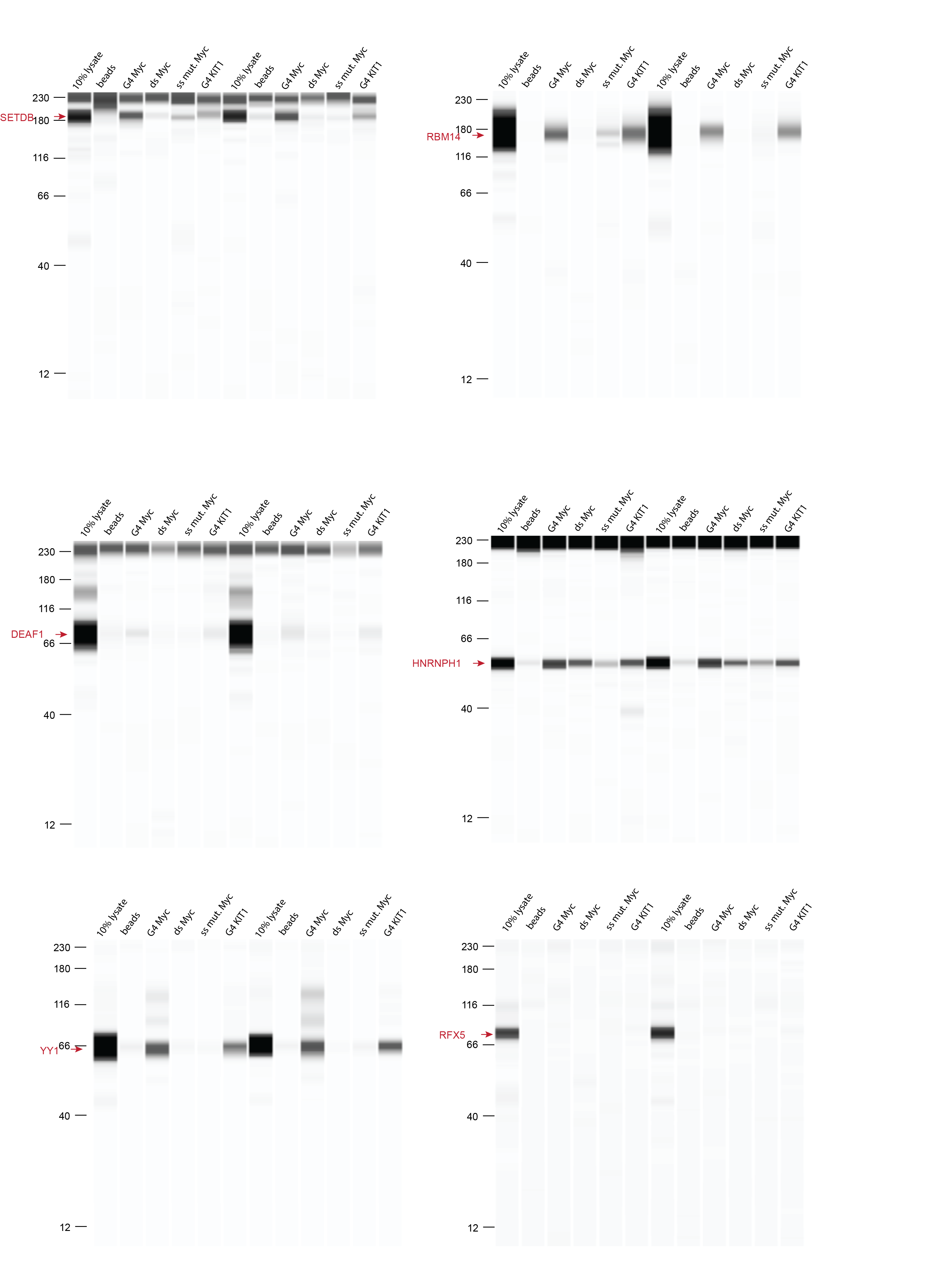
**

**
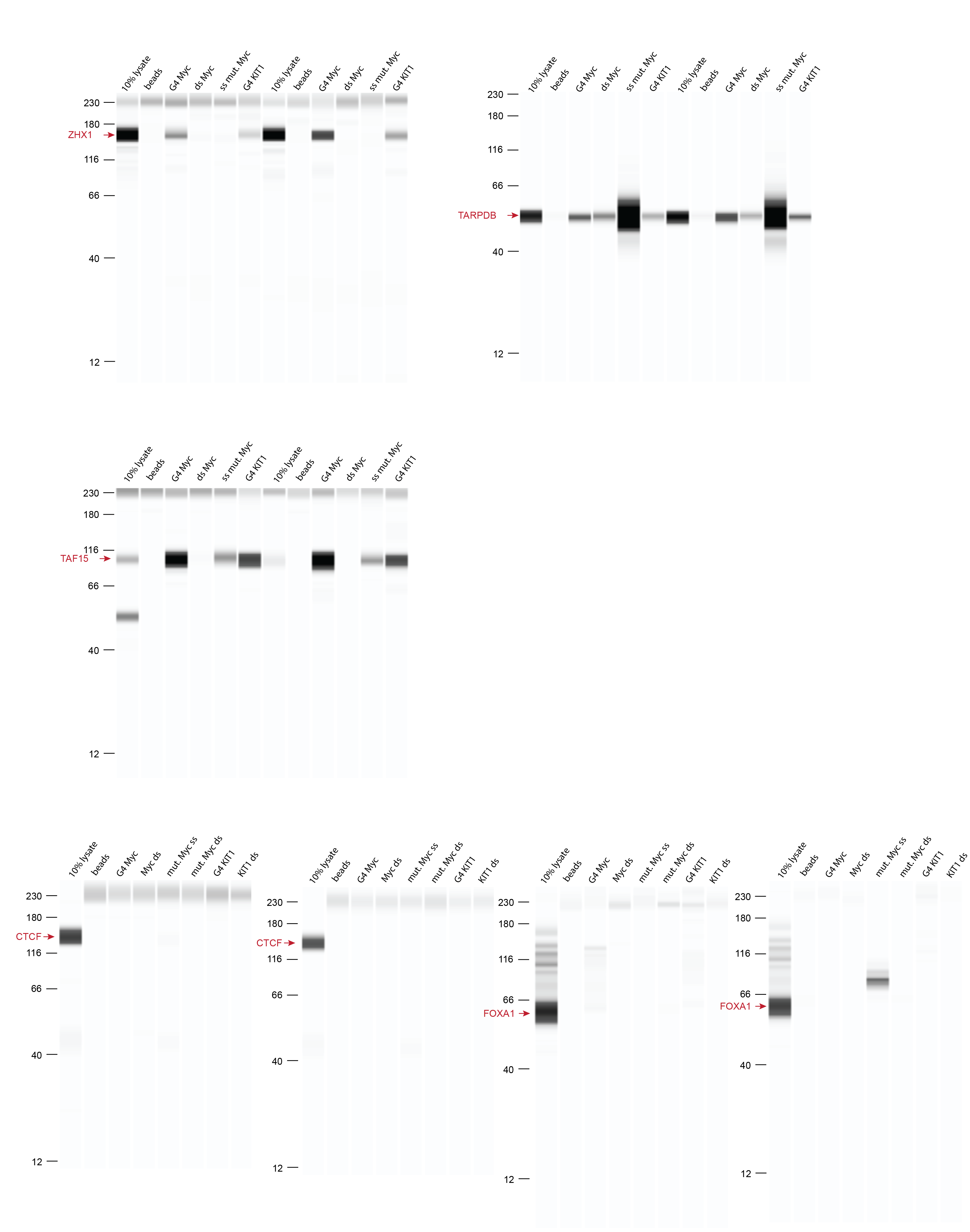
**

**
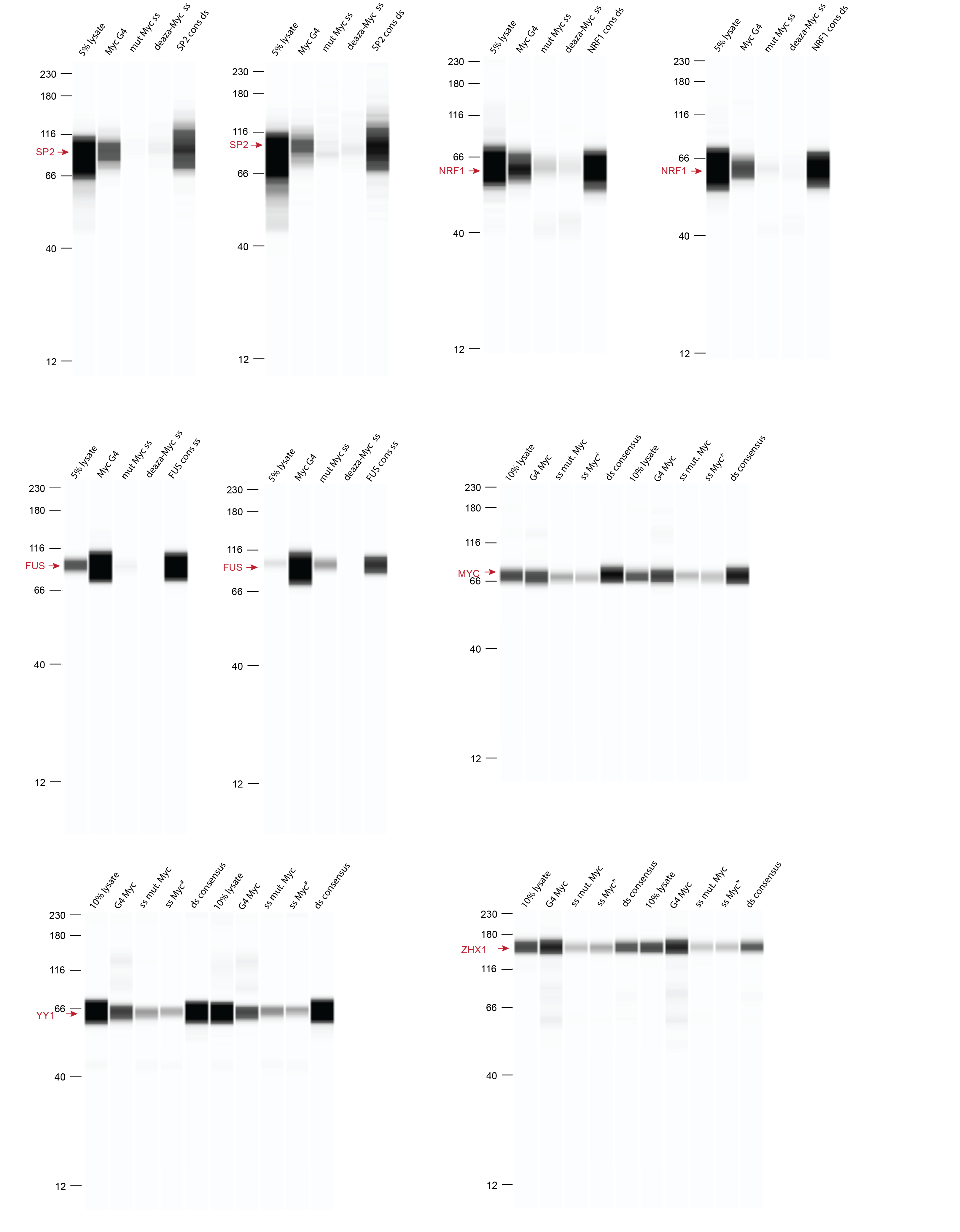
**

**
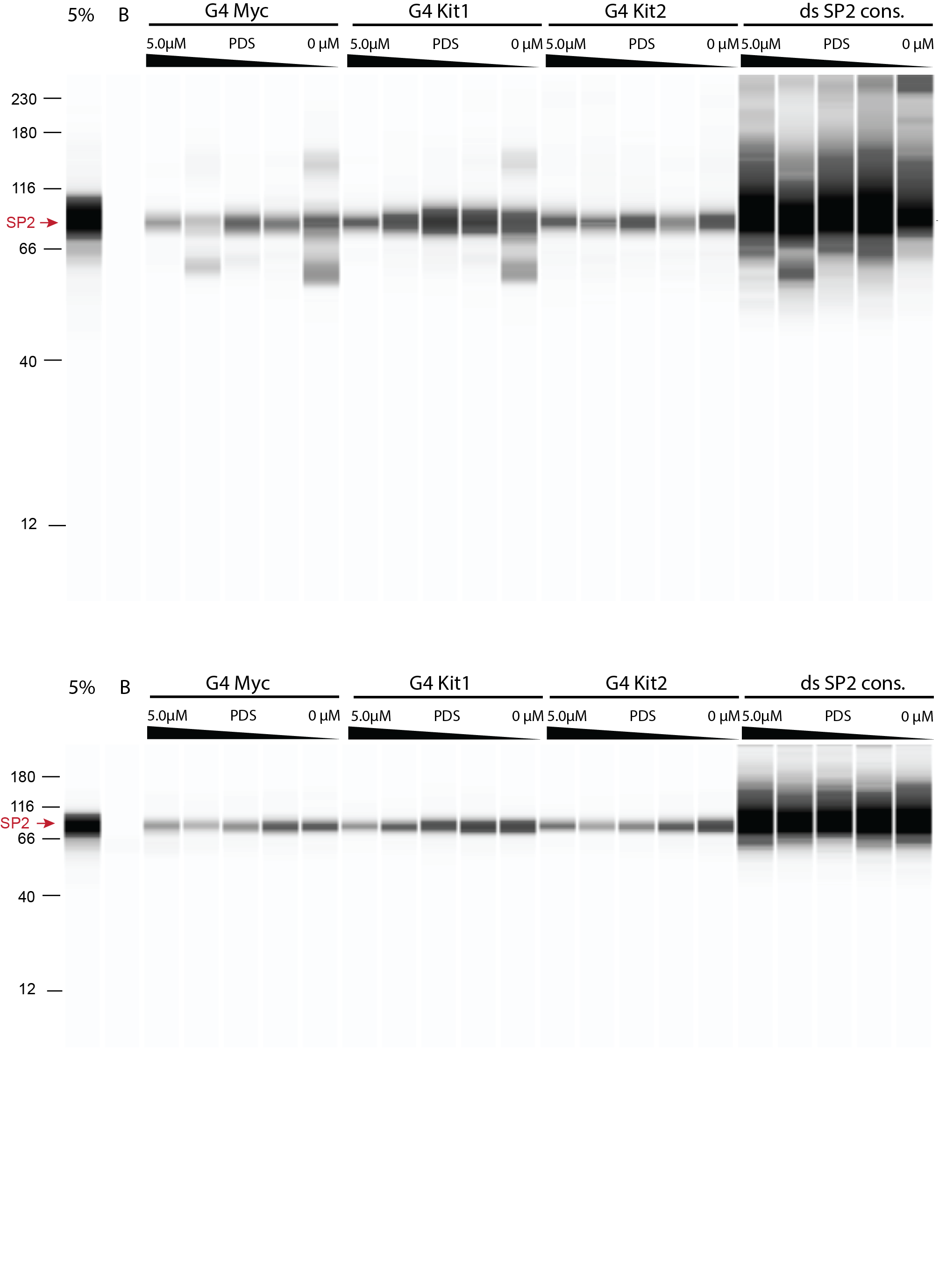
**

**
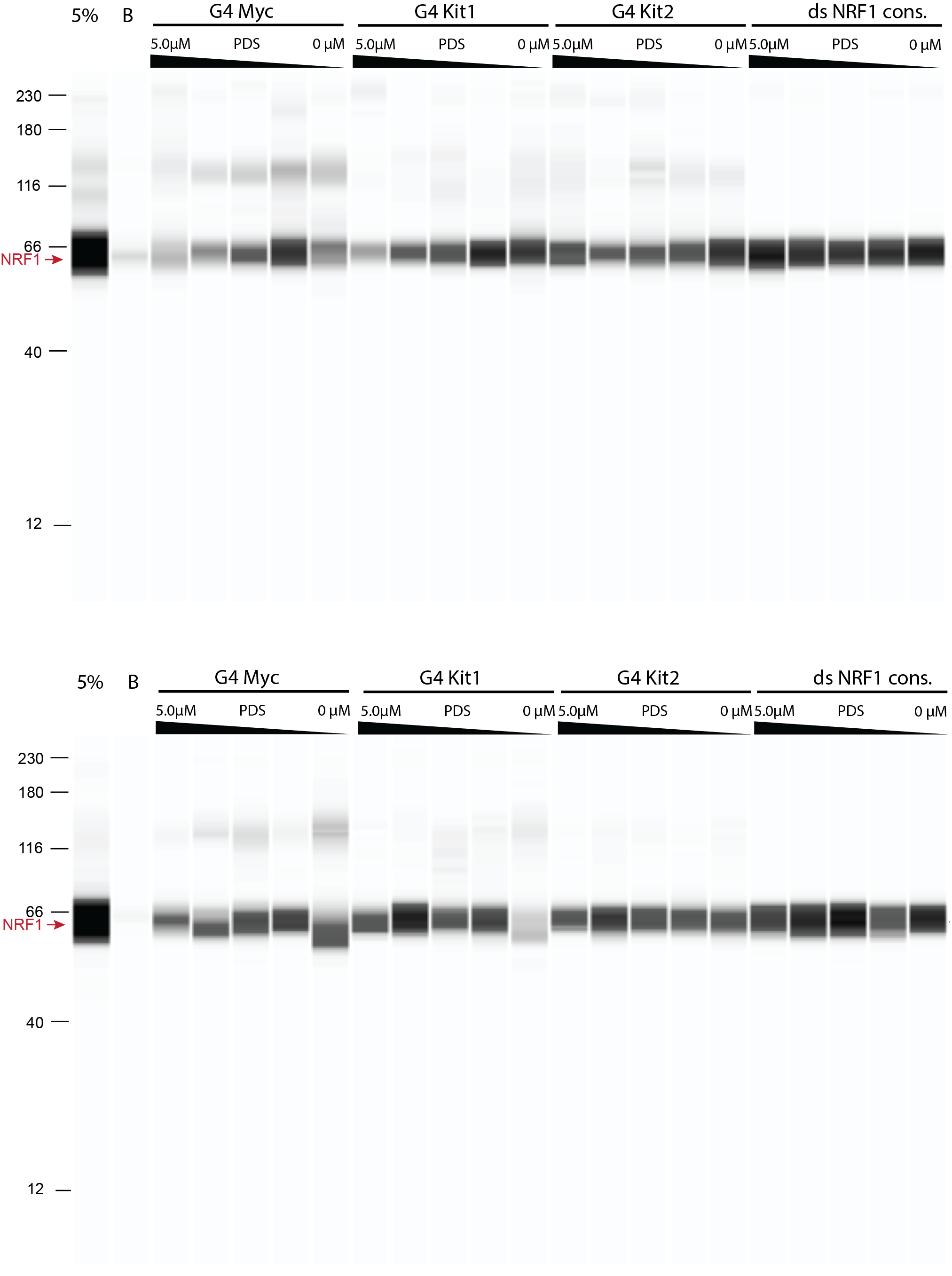
**

**
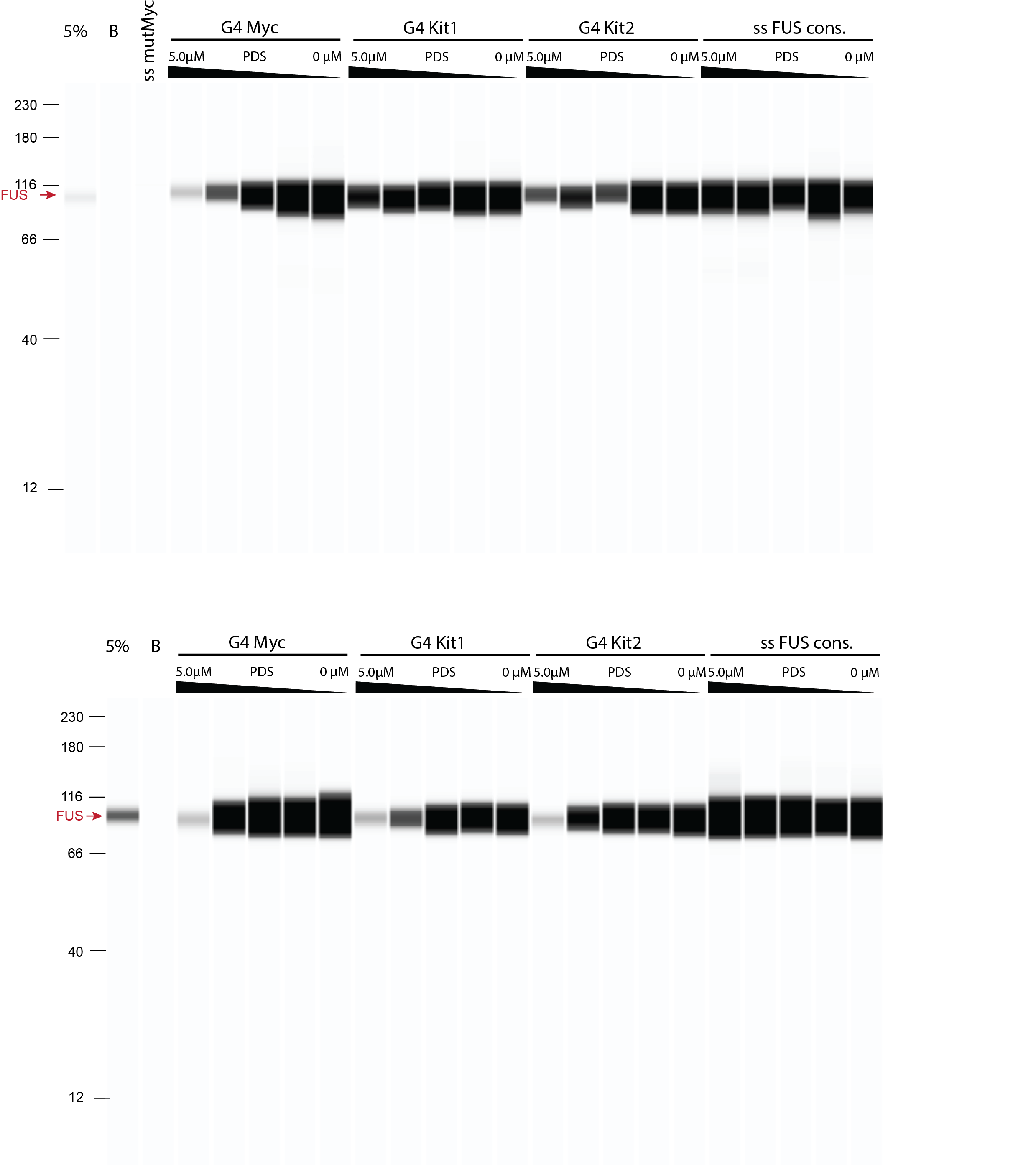
**

Supplement: Supplementary file 8 — Additional file 8. Uncropped western blotting analysis. [file 13059_2021_2324_MOESM8_ESM.docx]
