# Supplementary material for: G-quadruplexes are transcription factor binding hubs in human chromatin
Source: Genome Biol. 2021 Apr 23;22:117. doi: 10.1186/s13059-021-02324-z (PMC8063395; doi:10.1186/s13059-021-02324-z)
Supplement: Supplementary file 9 — Additional file 9. Review history. [file 13059_2021_2324_MOESM9_ESM.docx]

Review History

**First round of review**

**Reviewer 1**

**Are you able to assess all statistics in the manuscript, including the appropriateness of statistical tests used?**

No, I do not feel adequately qualified to assess the statistics.

**Comments to author:**

In this manuscript Spiegel and coauthors analyze the presence and location of G-quadruplexes (G4s) versus transcription factors (TF) binding sites. G4 analysis is acquired genome-wide in two cell lines by ChIP analysis with an anti-G4 antibody that this group previously developed, while TF binding sites data were acquired from ENCODE database. The bioinformatic analysis show that G4 regions are highly enriched in TF binding sites. The authors next prove that some TFs can directly bind to G4 forming sequences and that this interaction can be outcompeted with a G4 ligand. Based on this evidence, they propose G4s as TF binding hubs.

The work is very interesting since it provides new information on the function of G4s in cells. This group in general pioneers the research in the G4 field. However, more substantial experimental evidence to support the authors's main claim is required.

Comments:

The main issue with this work is with the number of TFs that have been considered to confirm the bioinformatic analysis. Only six TFs that bind to G4 have been tested in wet experiments. Of these six, two do not bind directly to G4s. Based on such a small set of data, no conclusion can actually be drawn. Is it possible that only a small number of TFs directly binds to G4s and the other TFs bind indirectly to the G4 region (by interacting with G4-binding TFs)? Which are the proteins that directly bind to G4s? Expand the wet analysis including a much higher number of TFs: about 30 randomly chosen TFs would provide a much better statistically significant indication of the actual percentage of TFs that bind to G4s.

In line with this, were all the TFs recovered by bioinformatic analysis centered at the center of the ChIP G4? Provide this type of information for all TFs. How many TFs actually bind to the G4 peak center? Provide percentage. How many bases around the G4 were considered?

Consensus sequences were tested only against three TFs. Consensus sequences were bound much better than G4s two times out of three, the one that binds best to G4 (FUS) binds also rather well to a ss sequence. This type of analysis has also to be expanded to the increased number of TFs, along with evaluation of the binding affinity.

In Fig S3a indicate which TFs have consensus sequences that can also form or be part of G4s.

Fig S3b and c "G4 ChIP-seq peaks and potential G4 around TSS": please show the extended sequence that can form the G4. G4 formation is hardly expected in the sequences where G bases are not prevalent (three sequences out of six).

Figure 4a shows hundreds of TFs associated to G4s. This result comes from bioinformatic analysis of datasets on the same cell lines but in likely different culturing/experimental conditions which may vary gene expression. The authors state that "More likely, the apparent co-localization for many of the peaks is due to the shuffling of several G4 binding events of various TFs across populations of large numbers of cells." This would imply that within the same cell population the expression of the same gene is controlled by hundreds of different TFs. Do the authors actually think this is the case? How many TFs can actually interact at a given G4 at a given time? Experimental evidence is required here. For instance, test the same 30 TFs on a specific G4 region at a given time: this should give a more reliable % value of the TFs that can actually interact in the same G4 region at the same time.

Also, in Figure 4a specify fraction of "G4" sites in the Y axis.

Despite a global higher expression level of genes that contain promoter G4s (Fig 4c) is shown, the same trend is reported when promoters are grouped according to the number of intersecting TFs (Fig 4b). It seems that there is no statistically significant difference in the expression of genes with more than 100 intersecting TFs, thus questioning the actual role of G4s in these cases. Seemingly, RNA Pol II occupancy is higher downstream of G4 depleted promoters for high density TFs genes (Fig S9), it is instead more prominent upstream of the TSS in G4 promoters. As the authors noted, G4s and R-loops are generally considered as obstacles for RNA pol processivity. Thus is it possible that the observed accumulation of RNA pol II is due to its slower release, mediated by G4s and R-loops? The authors need to measure RNA pol II release and nascent RNA accumulation.

In general, it has to be discerned whether G4s actually act as hub of TFs, as for the authors' hypothesis stated also in the manuscript title, or vice versa, if binding of TFs to G4 regions (not necessarily to G4s themselves) stimulates G4 folding. The requested experiments would likely shed light on this key aspect.

Legend to figure 3. Fix the f panel legend.

**Reviewer 2**

**Are you able to assess all statistics in the manuscript, including the appropriateness of statistical tests used?**

Yes, and I have assessed the statistics in my report.

**Comments to author:**

In this manuscript, the authors discover strong overlap between endogenous DNA quadruplexes (G4s) detected by the G4 specific antibody BG4 and endogenous transcription factors (TF) binding. G4 containing promoters are occupied on average by a larger number of TFs in comparison with G4-less promoters, forming so called "HOT regions". The authors have shown that G4 structures are able to pulldown some of these TFs from nuclear lysates. Affinity enrichment of these TFs from the nuclear extracts by G4 was inhibited by the G4-binding and -stabilizing molecule pyridostatin (PDS) suggesting that the TF-G4 interactions were outcompeted by this G4 selective molecule. Similarly, in the native chromatin context, treatment with PDS results in modest eviction of TFs from selected positive regions tested by ChIP-qPCR. The authors hypothesize that G4 structures are serving as a high traffic dock for the recruitment of many TFs to the same genomic region irrespectively of the primary target sequences.

This reviewer likes the design of the study, experimental output and the emerging hypothesis. The authors have presented more than enough evidence to consider the proposal of this bold hypothesis, but they have not presented sufficient evidence to establish its validity. At this stage either major clarifications and qualifications are required, or else considerably more genome-wide and biochemical analysis is required if they want to assert that their hypothesis is true.

1. Their genomic analysis is interesting and suggestive, but no more than that. The computational analyses of TF-binding to promoter G4s are not conclusive. That TFs are enriched at promoter G4s versus G4s 1 kb upstream may simply attest to TFs being recruited in part by the promoter-bound transcription machinery. And the greater recruitment of factors like the Sp's to promoter G4s than to promoter-randomized Sp-binding motifs (the JASPAR experiment which was not described) may reflect the fact that many G4's include multimerizations of the double stranded DNA binding motifs of "classical" TFs. In that event, is it surprising that a G4 that contains three SP-sites binds 2-3X more Sp than a single-Sp-motif?

2. In Figure 1, the authors indicate TFs with reported G4-binding ability. However, in the cited G4IPDB database the references for the NONO, HRNPK, SAFB and HNRNPL do not show that these TFs possess bona fide G4-interacting capacity (PMID: 23381195). In the case of hnRNPK, it has, in fact, been shown to bind the C-rich strand of the human MYC NHE1 site and not to bind with the G-rich strand. As the C-rich strand may form i-motif, binding to G4 may not be the regulatory driver here. (Nevertheless, the concept of binding to altered conformation is the same.) I was not able to find any other studies that report that NONO, SAFB or HNRNPL bind G4. As the authors noticed, these proteins, as well as FUS, and many others - U2AF1, GTF2F1, RBM15, POU5F1, NELFE, CEBPZ, FIP1L1, are single stranded nucleic acid binding proteins. High enrichment of TFs with this binding pattern is hard to explain only by quadruplex formation. This is unusual and requires additional analysis and explanation. These TFs cannot simply interact with single-stranded loop of quadruplex structure, otherwise PDS treatment would not have so profound effect (if I understand it right, the PDS is binding apical to G4 stack, therefore leaving loops available for binding). Additionally, these TFs- ssDNA/RNA interactions are structure and sequence specific. Consequently, the data presented in the manuscript might suggest the abundance of single-stranded nucleic acid structures in the vicinity of endogenous G4. What are those structures? The authors did not discuss the nature of these possible TFs target structures.

3. The authors have not sufficiently considered the emerging roles of R-loops as a source of altered DNA conformation at promoters. Current thinking is that G4 formation in living cells requires the negative torsional tension of the DNA and an associated stabilization of the opposite strand which is thought to be reached by R-loop formation. In the opinion of this reviewer, formation of R-loop structure in the vicinity of G4 might be an alternative and more logical explanation of the enrichment of single-stranded nucleic acid binding TFs at the studied sites. I assume the authors were aware of this possibility when they performed a comparative analysis of R-loop and G4 maps. But there are several issues that leave this issue equivocal. Their biochemical efforts to eliminate R-loop associated G4s are limited. The use only RNase A to sever RNA-G4s extending from R-loop regions (which assumes that these regions are in fact sensitive RNaseA. But they do not us RNase H to collapse the R-loops that might be stabilizing non-template strand G4s. Then they found that TFs binding centered at G4 but offset by 100 nt from the R-loop peaks. The authors conclude that offset location of R-loop indicates direct TFs binding with G4 rather than with R-loop. However, A) The authors analyze the subset of R-Seq data which they call R-loop confidence sites. R-seq data are generated with random priming of DNA fragment in the DNA/RNA hybrid. Random priming means that average location of R-loop peak in metadata will be shifted to the 5' of the DNA fragments. Similarly, if G4 structure and R-loop is formed at the same genomic location, G4 will be slightly upstream of the R-loop (exactly as seen in Figure S4a). If TFs of interest are binding with single-stranded nucleic acid of R-loop structure, then in metadata, the TF binding peak will appear on the left from R-loop site peak (exactly as seen in Figure S4b). For this reviewer, these are strong arguments that at least some of TFs might be targeted to the HOT regions through R-loop interaction. B) I disagree with the authors' conclusion that a subset of overlapping peaks identified by R-ChIP and DRIP-seq might be considered as R-loop confidence sites. These methods are completely different and produce markedly opposing results. R-ChIP and DRIP-seq data should be analyzed separately and, if possible, supplemented with orthogonal approach such as foot-printing. C) Association of R-loop with HOT regions had been reported. D) According to Figure S9, G4 is forming in the promoters with higher antisense transcription. Once again, one might expect that these are the regions prone to R-loop formation stabilizing G4 structures.

4. The authors associate G4s with transcription activation, but there are no tests of this assumption. The famous G4 in the human MYC gene has been largely considered a repressive motif. The work of Tan et al shows that upstream G4 folding is fostered by ongoing transcription and so could be a consequence, not a cause of high expression. Putting this together with the MYC NHE1 being a negative element might suggest that G4 formation is a mechanism to shut down ongoing transcription. Without more definitive tests of G4 function at several sites, the conclusion that it is an activating motif should be tempered a bit.

5. Though BG4 may be a valuable probe for G4 formation in vivo, it has nevertheless been suggested that it may sometimes recognize some other DNA conformations, a result that should be acknowledged (ACS Chem Biol . 2020 Apr 17;15(4):925-935).

6. The authors should discuss their work in relation to a recent study by Zheng et al who find some areas of agreement and others less so (Nucleic Acids Research, 2020 1doi: 10.1093/nar/gkaa841).

**Authors Response**

**Point-by-point responses to the reviewers’ comments:**

Referee 1

1)	The main issue with this work is with the number of TFs that have been considered to confirm the bioinformatic analysis. Only six TFs that bind to G4 have been tested in wet experiments. Of these six, two do not bind directly to G4s. Based on such a small set of data, no conclusion can actually be drawn. Is it possible that only a small number of TFs directly binds to G4s and the other TFs bind indirectly to the G4 region (by interacting with G4-binding TFs)? Which are the proteins that directly bind to G4s? Expand the wet analysis including a much higher number of TFs: about 30 randomly chosen TFs would provide a much better statistically significant indication of the actual percentage of TFs that bind to G4s.

*We have now greatly expanded our experiments by including an additional 27 TF candidates. These TFs were selected from the top 75 strongest enriched at endogenous G4s in K562 cells and also for the availability of antibodies suitable for use in the WES system. Overall, we now find that 18/27 of the new candidates (22/33 of all tested candidates) bind to G4s in nuclear lysates. These additional data firmly establishes that G4s recruit and/or co-recruit a variety of many different TFs and that this effect spans a substantial proportion of TFs.*

*The results of the additional affinity enrichment experiments are now included as Fig S. 7a in Supplementary Information and Table S2 and the following relevant text has been added to the manuscript (lines 170-194):*

*“G4-binding TFs were affinity captured from nuclear extracts from K562 cells using immobilized oligonucleotides followed by western blotting analysis using specific antibodies. Based on their enrichment at endogenous G4s in K562 and HepG2 cells, we selected 33 highly enriched TFs to investigate their G4 binding properties. Strikingly, a large fraction of TFs (22/33, 66 %) showed capacity to bind to G4 structures (Fig. 2a, Fig. S7 and Table S2). Most of the candidates bound to both Myc G4 and Kit1 G4, while few TFs (e.g. SRSF1, RBM15) had a preference for one G4 structure. Crucially, there was little or no binding to mutant single-stranded and double-stranded controls for the majority of G4-binding TFs (18/22). Furthermore, little or no binding was seen with a single-stranded 7-deazaguanine control sequence (ssMyc*) for the top enriched candidates (SP2, FUS and NRF1; Fig. 2b), which further confirms that G4 structure formation alone and not G-richness is required for binding. In contrast, four candidates (e.g. NONO) were more promiscuous and bound G4s to a similar extent to that of at least one of the control sequences, while TARDBP showed a very strong preference for single strand DNA. No detectable G4 binding was seen for some highly enriched TFs, such as E2F4 and CEBPZ, so these proteins may be recruited to G4s via other indirect interactions. Alternatively, structural features co-incident with endogenous G4s, such as i-motifs[34,35] or R-loops[36], possibly contribute to their recruitment. The enrichment level of SP2, NRF1, FUS, MYC, ZHX1 and YY1 was comparable to their binding to consensus sequence controls (Fig. 2b and S7b), which is in line with previous reports[35-37]. Importantly, two negative controls, FOXA1 and CTCF (Fig. 2a), that show low enrichment at endogenous G4s (Supplemental Data S1), did not bind to the G4 oligonucleotides, with CTCF also serving as a control due to its G-rich consensus binding motif.”*

*Fig. S7. TFs selectively bind to G4 structures.*

*a Affinity pull-down western blot analysis of different G4 oligonucleotides and control sequences. Genomic enrichment at endogenous G4s in K562 for randomization in open chromatin is shown in brackets.*

2)	In line with this, were all the TFs recovered by bioinformatic analysis centered at the center of the ChIP G4? Provide this type of information for all TFs. How many TFs actually bind to the G4 peak center? Provide percentage. How many bases around the G4 were considered?

*We have performed additional bioinformatics analysis on all available TF data sets to investigate the respective signal distribution around endogenous G4s.*

*Assessing the TF ChIP signal maximum relative to the G4 ChIP-seq peak center we calculate that 40/488 TFs are within a 10bp window, 100/488 TFs within a 20bp window and 177/488 TFs within a 40bp window. Notably, the resolution of published ChIP-seq experiments can be variable. For instance, in 7 different Myc ChIP-seq datasets for K562 cells, we observe varying signal maxima relative to G4s (-40, +10, +10, +20, +30, +40, +50), but overall our analysis strongly suggests that numerous TFs are physically positioned at the G4 site. We have now added Fig. S2e to show the distribution of all the TF binding sites relative to BG4 ChIP-seq sites in K562 cells and added the following text to the manuscript (lines 127-131):*

*“The average TF ChIP-seq signal for the strongest enriched TFs was also much higher at endogenous G4s, compared to control sites (Fig. 1d). Consistent with direct recruitment of TFs to G4 structures, the occupancy profile was generally centered around endogenous G4 sites for a large number of TFs (e. g. 100 TFs were within ±20 bp and 177 within ±40 bp) (Fig. S2e).”*

*e TF occupancy relative to G4 ChIP-seq sites in K562. Distance of maximum TF signal relative to the G4 peak center (x-axis) and enrichment at endogenous G4s in K562 cells (y-axis) is shown. TFs centered on G4s within ±20 bp are highlighted in blue (100/488 TFs).*

3)	Consensus sequences were tested only against three TFs. Consensus sequences were bound much better than G4s two times out of three, the one that binds best to G4 (FUS) binds also rather well to a ss sequence. This type of analysis has also to be expanded to the increased number of TFs, along with evaluation of the binding affinity.

*We have investigated further TF affinity enrichments using G4s and respective double strand consensus binding motifs. From our list of enriched TFs, we identified an additional 3 candidates, ZHX1, MYC and YY1, with well-defined consensus motifs. (Note that the majority of enriched TFs have not been reported as having simple consensus binding motifs1). Similar to our previous results, the new 3 selected candidates displayed strong enrichment by G4s. These results have been added to Fig. S7b and Table S3.*

*In-depth biochemical evaluation of individual TF-G4 binding interactions is beyond the scope of this manuscript, as functionally active TFs are not readily available and recombinant expression and purification of TFs is not practical at this scale. Earlier studies focusing on a single TF at a time have shown high affinity binding in vitro to both G4s and consensus motifs for several of the TFs featured and references to this have been added (e.g. SP12, YY13 and FUS4, line 191):*

*“The enrichment level of SP2, NRF1, FUS, MYC, YY1 and ZHX1 and was comparable to their binding to consensus sequence controls (Fig. 2b and S7b), which is mostly in line with previous reports[35–37].”*

*Fig. S7. TFs selectively bind to G4 structures.*

*b Affinity pull-down MYC, YY1 and ZHX1 including a single-stranded, G-rich oligomer unable to form a G4 structure (ss Myc*) and respective double-stranded DNA consensus sequences.*

4)	In Fig S3a indicate which TFs have consensus sequences that can also form or be part of G4s.

*We now highlight TF consensus sequence motifs, where available, that carry G-rich sequences that could be part of a predicted G4 motif (highlighted in green in Fig S3a):*

*a Consensus motifs for the top 20 candidates in K562 cells derived from ChIP-seq and SELEX experiments [1]. Many TFs (e.g. SP1, SP2, E2F4) display a G-rich binding motif (highlighted in green). Several top candidates are known to be single-strand binding proteins.*

5)	Fig S3b and c "G4 ChIP-seq peaks and potential G4 around TSS": please show the extended sequence that can form the G4. G4 formation is hardly expected in the sequences where G bases are not prevalent (three sequences out of six).

*Figure S3b and c show enriched short motifs calculated by MEME-ChIP using a maximum motif width of 40bp. MEME-ChIP is optimized to detect short TF binding motifs within a list of DNA sequences5. However, G4s have diverse topologies and can form sequences containing multiple G-runs that are interrupted by variable sequence and lengths of loops, which can all be recognized by the BG4 antibody with high affinity6. MEME-ChIP thus will potentially collapse diverse G4 sequences into one short conserved G-run element. This is also consistent with our earlier observations with BG4 on in HaCaT cells7.*

6)	Figure 4a shows hundreds of TFs associated to G4s. This result comes from bioinformatic analysis of datasets on the same cell lines but in likely different culturing/experimental conditions which may vary gene expression.

*While these may be confounding factors, ENCODE remains the gold-standard resource for comparative studies of TFs and chromatin features. A major advantage is the comparability of different datasets due to standardized protocols and analyses guidelines8 that we also followed.*

The authors state that "More likely, the apparent co-localization for many of the peaks is due to the shuffling of several G4 binding events of various TFs across populations of large numbers of cells." This would imply that within the same cell population the expression of the same gene is controlled by hundreds of different TFs. Do the authors actually think this is the case? How many TFs can actually interact at a given G4 at a given time?

*Interesting questions. So far, structural information of G4s bound to full-length proteins only exists for a helicase and a yeast telomeric protein, both interacting with 1:1-stoichiometry9,10. These data would suggest that only a small numbers of proteins could directly contact G4 simultaneously. However, we note that co-operative binding has been described for TFs11 raising the possibility that certain TFs may indeed co-bind G4 loci. Our proposal that dozens of TFs dynamically compete for the same genomic site also provides a structural explanation to support a recent model of many TFs binding to so-called HOT regions12. As the shared binding sites did not match known consensus sequences, it was speculated that there was a role for a common chromatin feature. We now provide a possible explanation for this phenomenon. We have now amended the main text as follows (line 263-268):*

*“It is unlikely that dozens or hundreds of TFs simultaneously co-occupy a genomic locus in a single large molecular complex in the same cell [47]. While certain TFs may bind their targets cooperatively[4], it is more likely that many TFs dynamically compete for binding to the same G4 locus. In a large population of cells, this would result in apparent co-localization at the same site due to signal averaging across the cellular population.”*

Experimental evidence is required here. For instance, test the same 30 TFs on a specific G4 region at a given time: this should give a more reliable % value of the TFs that can actually interact in the same G4 region at the same time.

*While this is an interesting idea, the practicalities of such a measurement are extremely challenging and well beyond the scope of this manuscript. This would require single-cell ChIP for multiple TFs, something that is not currently possible.*

7)	Also, in Figure 4a specify fraction of "G4" sites in the Y axis.

*Corrected.*

8)	Despite a global higher expression level of genes that contain promoter G4s (Fig 4c) is shown, the same trend is reported when promoters are grouped according to the number of intersecting TFs (Fig 4b). It seems that there is no statistically significant difference in the expression of genes with more than 100 intersecting TFs, thus questioning the actual role of G4s in these cases.

*A key finding of our study is that while HOT sites can exist in promoters independently of G4s and show high expression levels13, promoters featuring endogenous G4s have a much higher propensity to recruit large numbers of TFs.*

*Fig. 4a and b show that on average, a lot more TFs bind at endogenous G4s than at control sites in both cell lines. This data provides an explanation for overall higher average transcription observed at G4-positive promoters. We have rephrased this point (line 252-256):*

*“Furthermore, we found that as the number of TFs binding at endogenous G4s increased so did RNA Polymerase 2 occupancy and transcriptional activity (Fig. 4b and Fig. S10). A similar correlation was observed for promoters lacking G4s, but it should be noted that endogenous G4s are considerably more occupied by a greater number of TFs (see different categories in Fig. 4b).”*

Seemingly, RNA Pol II occupancy is higher downstream of G4 depleted promoters for high density TFs genes (Fig S9), it is instead more prominent upstream of the TSS in G4 promoters. As the authors noted, G4s and R-loops are generally considered as obstacles for RNA pol processivity. Thus is it possible that the observed accumulation of RNA pol II is due to its slower release, mediated by G4s and R-loops? The authors need to measure RNA pol II release and nascent RNA accumulation.

*It is expected that genes with higher expression would feature higher levels of RNA Pol II occupancy surrounding their promoter. Moreover, previous experiments on G4 containing plasmids14,15 found that G4s inhibit polymerase progression, if they are located on the template strand in gene bodies, but not if they are on the non-template strand. When we derived the strandedness of endogenous G4s from stranded data of potential G4s16 (see METHODS) and repeated the Pol II analysis, we also saw a similar occupancy profile, even if the G4 was located in the non-templated strand (see below). This suggests that the increase in Pol II signal upstream of TSS is not due to stalling at G4s. Despite this, we acknowledge that there is enrichment of Pol II upstream of the TSS in regions close to or overlapping endogenous G4s. In-depth mechanistic investigations on the interplay between G4s, TF recruitment and Pol II kinetics is a focus for future work.*

*RNA Polymerase 2 occupancy does not depend on strandedness of G4s.*

*PolR2A ChIP-seq signal (ENCODE ENCFF000YWV) distribution around theTSS according to different numbers of TF occupancy categories in K562 cells. Occupancy profiles are shown for promoters that contain an endogenous G4 on the non-template (left) or template strand(right). The strandedness of endogenous G4s was derived from stranded data of sequences with G4-forming potential. (Figure not included in manuscript)*

In general, it has to be discerned whether G4s actually act as hub of TFs, as for the authors' hypothesis stated also in the manuscript title, or vice versa, if binding of TFs to G4 regions (not necessarily to G4s themselves) stimulates G4 folding. The requested experiments would likely shed light on this key aspect.

*A recent study has suggested that formation of endogenous G4s is independent of active transcription and occurs prior to transcriptional initiation17. How exactly G4 formation is regulated in a chromatin context remains a major question to be addressed by the field in future studies. This will include whether, for example, certain pioneer TFs, chromatin structure or biophysical mechanisms promote G4 formation at promoters in cells are subjects for future studies.*

Legend to figure 3. Fix the f panel legend.

*Corrected.*

Referee 2

1.	Their genomic analysis is interesting and suggestive, but no more than that. The computational analyses of TF-binding to promoter G4s are not conclusive. That TFs are enriched at promoter G4s versus G4s 1 kb upstream may simply attest to TFs being recruited in part by the promoter-bound transcription machinery. And the greater recruitment of factors like the Sp's to promoter G4s than to promoter-randomized Sp-binding motifs (the JASPAR experiment which was not described) may reflect the fact that many G4's include multimerizations of the double stranded DNA binding motifs of "classical" TFs. In that event, is it surprising that a G4 that contains three SP-sites binds 2-3X more Sp than a single-Sp-motif?

*While we agree that ChIP-seq alone only provides genomic associations indicative of binding interactions, we have now provided extensive additional biophysical evidence that a G4 structure itself is able to bind many different TF (ca. 66% of 33 candidate TFs bind G4s).*

*In our bioinformatics analyses we compared TF occupancy at endogenous G4s (i.e. folded G4s experimentally mapped in chromatin) versus potential G4s (sequences that can form folded G4s, but do not in this cell line) located in G-rich, open chromatin promoters. For many TFs we observed much stronger enrichment at the endogenous G4s showing the importance of the G4 structure formation (see Fig. 1c). Potential G4s have essentially identical GC-content to endogenous, folded G4s so underlying recruitment by the promoter-bound transcription machinery should be very similar. Note also while endogenous G4s can theoretically contain multiple SP-binding motifs, this is also the case for the sites of potential G4s, which were used as the control. Furthermore, we also find significant enrichment at endogenous G4s for TFs (e.g. NRF1) that have no reported G-rich consensus binding sequence, again supporting a structure rather than sequence-based recognition mechanism.*

*TF enrichment at endogenous G4s and consensus binding sites from JASPAR is described in the main text (line 144-153) and in the Methods section.*

2.	In Figure 1, the authors indicate TFs with reported G4-binding ability. However, in the cited G4IPDB database the references for the NONO, HRNPK, SAFB and HNRNPL do not show that these TFs possess bona fide G4-interacting capacity (PMID: 23381195). In the case of hnRNPK, it has, in fact, been shown to bind the C-rich strand of the human MYC NHE1 site and not to bind with the G-rich strand. As the C-rich strand may form i-motif, binding to G4 may not be the regulatory driver here. (Nevertheless, the concept of binding to altered conformation is the same.) I was not able to find any other studies that report that NONO, SAFB or HNRNPL bind G4. As the authors noticed, these proteins, as well as FUS, and many others - U2AF1, GTF2F1, RBM15, POU5F1, NELFE, CEBPZ, FIP1L1, are single stranded nucleic acid binding proteins. High enrichment of TFs with this binding pattern is hard to explain only by quadruplex formation. This is unusual and requires additional analysis and explanation. These TFs cannot simply interact with single-stranded loop of quadruplex structure, otherwise PDS treatment would not have so profound effect (if I understand it right, the PDS is binding apical to G4 stack, therefore leaving loops available for binding). Additionally, these TFs- ssDNA/RNA interactions are structure and sequence specific. Consequently, the data presented in the manuscript might suggest the abundance of single-stranded nucleic acid structures in the vicinity of endogenous G4. What are those structures? The authors did not discuss the nature of these possible TFs target structures.

*There is debate in the literature on the binding properties of individual proteins and this will require detailed biophysical and structural validation beyond the scope of this manuscript. The G4IPDB is currently the most comprehensive resource on reported G4-protein interactions and does in fact catalogue NONO, SAFB and HNRNPL as G4-binders based on the specified reference (PMID: 23381195)18. We agree that the referenced proteomics study would have benefited from further validation. However, NONO has recently been validated to bind G4s elsewhere19. We agree that “HNRNPK” has since then been shown to have a preference for i-motifs and have thus removed it from line 110. This now reads:*

*“This analysis showed that many TFs were enriched at endogenous G4 sites and is immediately suggestive of direct TF-G4 interactions (Fig. 1a and Fig. S2), particularly since several of the most enriched proteins, such as FUS and SP1, have previously been suggested to interact with DNA G4s in vitro [20].”*

*In principle, some single-stranded binding proteins might bind loops in G4s or 5’ or 3’ single strand features at the junction of G4s and double strand DNA, which might be physiologically relevant. However, as several single-stranded binding proteins did not bind to G4 baits in our affinity enrichment analysis, this suggests that single-stranded binding is not a general feature. Furthermore, the use of 2 different G4s suggests that the precise sequence per se is not required for binding. Indeed, we have not made any judgement on the exact binding mode and the referee rightly points out that our chemical intervention validates that G4 structure itself as an element of recognition.*

*We have added a note on the potential contribution of alternative DNA structural features to the manuscript (line 187-189):*

*“Alternatively, structural features co-incident with endogenous G4s, such as i-motifs[32,33] or R-loops[34], possibly contribute to their recruitment.”*

3.	The authors have not sufficiently considered the emerging roles of R-loops as a source of altered DNA conformation at promoters. Current thinking is that G4 formation in living cells requires the negative torsional tension of the DNA and an associated stabilization of the opposite strand which is thought to be reached by R-loop formation. In the opinion of this reviewer, formation of R-loop structure in the vicinity of G4 might be an alternative and more logical explanation of the enrichment of single-stranded nucleic acid binding TFs at the studied sites. I assume the authors were aware of this possibility when they performed a comparative analysis of R-loop and G4 maps. But there are several issues that leave this issue equivocal. Their biochemical efforts to eliminate R-loop associated G4s are limited. The use only RNase A to sever RNA-G4s extending from R-loop regions (which assumes that these regions are in fact sensitive RNaseA. But they do not us RNase H to collapse the R-loops that might be stabilizing non-template strand G4s.

*RNase A treatment is employed to eliminate DNA/RNA-hybrid G4s and RNA G4s prior to performing G4 ChIP with the BG4 antibody. Conversely, we have no evidence that the BG4 antibody has any particular affinity towards R-loops, so it is unlikely that R-loops themselves will lead to false positive peaks in G4 ChIP experiments and thus do not need to be removed. Notably, endogenous G4s mapped by G4 ChIP and R-loops mapped by R-ChIP are clearly shifted by ~140 bp (see Fig. S4a), making it unlikely that BG4 captures R-loops.*

*We recognise that previous work has indicated, a potential crosstalk between G4s and R-loops20. Therefore, we do not disagree that there may be physiologically relevant networks of G4s, transcription and R-loops in chromatin. However, our study aims to investigate the interaction of TFs and G4s. Perturbing the native G4 landscape by removal of R-loops prior to G4 ChIP by genetic manipulation of cells may help to further elucidate the relationship between G4s and R-loops, but this is beyond the scope of the current study.*

Then they found that TFs binding centered at G4 but offset by 100 nt from the R-loop peaks. The authors conclude that offset location of R-loop indicates direct TFs binding with G4 rather than with R-loop. However, A) The authors analyze the subset of R-Seq data which they call R-loop confidence sites.

*This data set for high confidence R-loops in K562 cells was obtained from GEO (GSE97072) as provided by the authors of the original R-ChIP study21.*

R-seq data are generated with random priming of DNA fragment in the DNA/RNA hybrid. Random priming means that average location of R-loop peak in metadata will be shifted to the 5' of the DNA fragments. Similarly, if G4 structure and R-loop is formed at the same genomic location, G4 will be slightly upstream of the R-loop (exactly as seen in Figure S4a). If TFs of interest are binding with single-stranded nucleic acid of R-loop structure, then in metadata, the TF binding peak will appear on the left from R-loop site peak (exactly as seen in Figure S4b). For this reviewer, these are strong arguments that at least some of TFs might be targeted to the HOT regions through R-loop interaction.

*R-ChIP sample preparation and bioinformatics analysis specifically aims to mitigate such technical issues21,22: First, the protocol employs Phi29 DNA polymerase for primer extension to generate dsDNA. Phi29 has strand displacement activity, which means it will displace all shorter DNA segments that are initiated by internal priming (see example figure below). Therefore, the length of the resulted dsDNA should be very close to its original length. Second, R-ChIP libraries are sequenced in a 40bp single-end strand-specific manner. It is not possible to tell where the 5' end of template DNA (or the downstream R-loop boundary) is located, but the reads are computationally extended to the estimated average size of DNA fragments. In addition, the genomic positions of the resulting R-loop peaks revealed a strong G/C skew in the non-template DNA, a sequence feature that has previously been linked to R-loop formation23. Overall, this makes it very unlikely that “bone fide R-loops are located upstream of the TSS, where the majority of endogenous G4s are found. We have also consulted with the authors of the original R-ChIP study to verify this assessment.*

*While most TFs are centred upstream the TSS at endogenous G4s, some are found at R-loop high confidence sites, thus these might also interact via R-loops (see below). We have now amended the text as follows (Line 137-143):*

*“R-loops were located mostly on the opposite strand and downstream (~140 bp) of the G4s (Fig. S4a). While some TFs appear to be enriched downstream of endogenous G4s, possibly indicating interactions with R-loops, the majority of TFs are found at endogenous G4s upstream of the TSS (Fig. S2e). Importantly, ChIP signal profiles for TF highly enriched at G4s, such as SP2, E2F4, NRF1 or FUS, were found to be centered on the G4s rather than R-loops supporting a direct recruitment to G4s rather than R-loops (Fig S4b).”*

*and the discussion (line 319-322):*

*“We propose a fundamental mechanism of transcriptional regulation, that may apply to many genes, whereby G4 structures recruit a multitude of TFs causing more frequent engagement of TFs in promoters and thereby stimulating transcriptional output (Fig. 4d). Further studies will be needed to explore exactly what mechanism maintains the endogenous G4 landscape in chromatin[54] and whether other DNA structural features also contribute to this phenomenon.”*

*(For reviewers only)*

B) I disagree with the authors' conclusion that a subset of overlapping peaks identified by R-ChIP and DRIP-seq might be considered as R-loop confidence sites. These methods are completely different and produce markedly opposing results. R-ChIP and DRIP-seq data should be analyzed separately and, if possible, supplemented with orthogonal approach such as foot-printing.

*Ideally, different approaches to map R-loops should provide similar results. However, we appreciate that R-ChIP and DRIP-seq data show considerable differences in peak widths and distribution22. This raises questions on whether sites that are only detected by one method are true R-loops or technical artefacts. We did not want to dilute our analysis with confounding data leading to noise and so focused on the highest confidence R-loop sites. These should represent the most stable R-loops in chromatin, regions at which potential TF interactions would be the strongest. At these sites we observe a clear difference between endogenous G4s and R-loops (see previous comment above).*

C) Association of R-loop with HOT regions had been reported. D) According to Figure S9, G4 is forming in the promoters with higher antisense transcription. Once again, one might expect that these are the regions prone to R-loop formation stabilizing G4 structures.

*As stated above, we acknowledge that R-loops are associated with G4s and may stabilize G4s. This possibly could influence HOT region binding at R-loops, however, a large number of TFs binding sites are centred on endogenous G4s upstream of the TSS rather than R-loops (see above). The distribution of TF binding sites reveals that some are enriched downstream of G4s raising the possibility interactions with R-loops (see plot below). Additional investigation and discussion on R-loop specific interactors would be the scope of a future study.*

*TF occupancy relative to R-ChIP confidence sites in K562 cells. Distance of maximum TF occupancy relative to the R-ChIP peak center (x-axis) and enrichment at endogenous G4s in K562 (y-axis) is shown. TFs centered on R-loops within ±20 bp are highlighted in purple. (For reviewers only)*

4.	The authors associate G4s with transcription activation, but there are no tests of this assumption. The famous G4 in the human MYC gene has been largely considered a repressive motif. The work of Tan et al shows that upstream G4 folding is fostered by ongoing transcription and so could be a consequence, not a cause of high expression. Putting this together with the MYC NHE1 being a negative element might suggest that G4 formation is a mechanism to shut down ongoing transcription. Without more definitive tests of G4 function at several sites, the conclusion that it is an activating motif should be tempered a bit.

*Early work on MYC suggested G4s might inhibit transcription. However, in these experiments are not definitive and may actually derive an incorrect conclusion based on the methods used at the time. For example, the use of G4-stabilizing ligands has been shown to inhibit transcription in some cases, however a liganded G4 is not the same as an unliganded G4 and the ligand may well interfere with protein (e.g. TF) recruitment, whereas the unliganded G4 may recruit proteins and activate transcription. Also studies that use G4s in plasmid constructs (such as the original MYC experiments) lack chromatin context and also did not confirm actual G4 folded structure at those sites in cellular chromatin. We acknowledge that G4 formation in gene bodies could impair polymerase progression as suggested by in vitro work, but we reiterate that we observe a large fraction of endogenous G4s at promoters. We and others24 have now demonstrated that in native chromatin, endogenous G4s occur at promoters of highly expressed genes. An additional reference has been added to the manuscript in line 314.*

*“In contrast, endogenous promoter G4s are predominantly found at highly active genes [16,17]”*

*In addition, a recent study suggested that endogenous G4 formation happens independently of transcription and prior to transcriptional initiation.17 We have now added the following text to the discussion (line 318-321):*

*“Further studies will be needed to explore exactly what mechanism maintains the endogenous G4 landscape in chromatin[54] and whether other DNA structural features also contribute to this phenomenon..”*

5.	Though BG4 may be a valuable probe for G4 formation in vivo, it has nevertheless been suggested that it may sometimes recognize some other DNA conformations, a result that should be acknowledged (ACS Chem Biol . 2020 Apr 17;15(4):925-935).

*The commercial IgG version of BG4 appears to show some off-target binding towards other DNA structures such as C-rich single-stranded sequences.25,26*

*However, in our hands and others27 no notable off-target binding has been seen with the original scFv version of BG4, which we generated, characterised and used in the present study.*

6.	The authors should discuss their work in relation to a recent study by Zheng et al who find some areas of agreement and others less so (Nucleic Acids Research, 2020 1doi: 10.1093/nar/gkaa841).

*We have now added the following discussion to the manuscript (line 287-296):*

*“Recently, endogenous expression of a small, engineered G4-binding protein was reported for detection of DNA G4s via ChIP-seq in human cells[49]. This alternative mapping approach observed G4s to be enriched at promoters, associated with highly expressed genes, and enrichment of certain proteins (FUS, TAF15, RBM14, TARDBP, HNRNPK, PCBP1) at G4 loci. In contrast to G4 ChIP-seq on fixed chromatin, the study mapped over 100,000 G4s and observed considerable G4 formation downstream of the TSS in addition to promoter G4s. Endogenous expression of a probe may be able to detect weaker, more transient G4s. However, it may also perturb the endogenous G4 landscape and shift the equilibrium to stabilise G4s that do not normally form under physiological conditions. “*

*References*

*1.	Lambert, S. A. et al. The Human Transcription Factors. Cell 172, 650–665 (2018).*

*2.	Raiber, E. A., Kranaster, R., Lam, E., Nikan, M. & Balasubramanian, S. A non-canonical DNA structure is a binding motif for the transcription factor SP1 in vitro. Nucleic Acids Res. 40, 1499–1508 (2012).*

*3.	Li, L. et al. YY1 interacts with guanine quadruplexes to regulate DNA looping and gene expression. Nat. Chem. Biol. (2020). doi:10.1038/s41589-020-00695-1*

*4.	Yagi, R., Miyazaki, T. & Oyoshi, T. G-quadruplex binding ability of TLS/FUS depends on the β-spiral structure of the RGG domain. Nucleic Acids Res. 46, 5894–5901 (2018).*

*5.	Grant, C. E., Bailey, T. L. & Noble, W. S. FIMO: Scanning for occurrences of a given motif. Bioinformatics 27, 1017–1018 (2011).*

*6.	Biffi, G., Tannahill, D., McCafferty, J. & Balasubramanian, S. Quantitative visualization of DNA G-quadruplex structures in human cells. Nat. Chem. 5, 182–6 (2013).*

*7.	Hänsel-Hertsch, R. et al. G-quadruplex structures mark human regulatory chromatin. Nat. Genet. 48, 1267–1272 (2016).*

*8.	Myers, R. M. et al. A user’s guide to the Encyclopedia of DNA elements (ENCODE). PLoS Biol. 9, (2011).*

*9.	Chen, M. C. et al. Structural basis of G-quadruplex unfolding by the DEAH/RHA helicase DHX36. Nature 558, 465–483 (2018).*

*10.	Traczyk, A., Liew, C. W., Gill, D. J. & Rhodes, D. Structural basis of G-quadruplex DNA recognition by the yeast telomeric protein Rap1. Nucleic Acids Res. 48, 4562–4571 (2020).*

*11.	Jolma, A. et al. DNA-dependent formation of transcription factor pairs alters their binding specificity. Nature 527, 384–388 (2015).*

*12.	Partridge, E. C. et al. Occupancy maps of 208 chromatin-associated proteins in one human cell type. Nature 583, 720–728 (2020).*

*13.	Li, H., Liu, F., Ren, C., Bo, X. & Shu, W. Genome-wide identification and characterisation of HOT regions in the human genome. BMC Genomics 17, 1–16 (2016).*

*14.	Agarwal, T., Roy, S., Kumar, S., Chakraborty, T. K. & Maiti, S. In the sense of transcription regulation by G-quadruplexes: Asymmetric effects in sense and antisense strands. Biochemistry 53, 3711–3718 (2014).*

*15.	Holder, I. T. & Hartig, J. S. A matter of location: Influence of G-quadruplexes on escherichia coli gene expression. Chem. Biol. 21, 1511–1521 (2014).*

*16.	Chambers, V. S. et al. High-throughput sequencing of DNA G-quadruplex structures in the human genome. Nat. Biotechnol. 33, 877–881 (2015).*

*17.	Roychoudhury, S. et al. Endogenous oxidized DNA bases and APE1 regulate the formation of G-quadruplex structures in the genome. Proc. Natl. Acad. Sci. 201912355 (2020). doi:10.1073/pnas.1912355117*

*18.	Mori, K. et al. HnRNP A3 binds to GGGGCC repeats and is a constituent of p62-positive/TDP43-negative inclusions in the hippocampus of patients with C9orf72 mutations. Acta Neuropathol. 125, 413–423 (2013).*

*19.	Simko, E. A. J. et al. G-quadruplexes offer a conserved structural motif for NONO recruitment to NEAT1 architectural lncRNA. Nucleic Acids Res. 1–18 (2020). doi:10.1093/nar/gkaa475*

*20.	De Magis, A. et al. DNA damage and genome instability by G-quadruplex ligands are mediated by R loops in human cancer cells. Proc. Natl. Acad. Sci. U. S. A. (2018). doi:10.1073/pnas.1810409116*

*21.	Chen, L. et al. R-ChIP Using Inactive RNase H Reveals Dynamic Coupling of R-loops with Transcriptional Pausing at Gene Promoters. Mol. Cell 68, 745-757.e5 (2017).*

*22.	Chen, J.-Y., Zhang, X., Fu, X.-D. & Chen, L. R-ChIP for genome-wide mapping of R-loops by using catalytically inactive RNASEH1. Nat. Protoc. 9, (2019).*

*23.	Ginno, P. A., Lott, P. L., Christensen, H. C., Korf, I. & Chédin, F. R-Loop Formation Is a Distinctive Characteristic of Unmethylated Human CpG Island Promoters. Mol. Cell 45, 814–825 (2012).*

*24.	Kouzine, F. et al. Permanganate/S1 Nuclease Footprinting Reveals Non-B DNA Structures with Regulatory Potential across a Mammalian Genome. Cell Syst. 4, 344-356.e7 (2017).*

*25.	Zeraati, M. et al. I-motif DNA structures are formed in the nuclei of human cells. Nat. Chem. 10, 631–637 (2018).*

*26.	Ray, S. et al. Custom DNA Microarrays Reveal Diverse Binding Preferences of Proteins and Small Molecules to Thousands of G-Quadruplexes. ACS Chem. Biol. 15, 925–935 (2020).*

*27.	Javadekar, S. M., Nilavar, N. M., Paranjape, A., Das, K. & Raghavan, S. C. Characterization of G-quadruplex antibody reveals differential specificity for G4 DNA forms. DNA Res. (2020). doi:10.1093/dnares/dsaa024*

**Second round of review**

**Reviewer 1**

The authors have experimentally validated additional 27 transcription factors (TFs) providing much more solid support to their bioinformatic analysis.

I though deem that experimental evidence on the TFs that interact at a given G4 is still required, to support the bioinformatics data that indicate a very peculiar and not so predictable feature of TF interaction at G4s, based on our current understanding of the system. There is no need to go single cell and the experiments to be performed are not very demanding: just measure by ChIP one or two G4 regions with the available anti-TF antibodies (those that have been tested in western blot would do nicely) by qPCR. This experiment would give a percentage of TFs (over the tested ones) that do actually interact at a given G4 region in a cell population.

In Fig S3e there are two Sp1 and two NRF1 signals. Is there a reason for this?

Fix this sentence “The enrichment level of SP2, NRF1, FUS, MYC, YY1 and ZHX1 and was comparable to their binding to consensus sequence controls (Fig. 2b and S7b),….” in the text.

**Reviewer 2**

The manuscript is improved, but several overstatements and other points still need to be addressed.

Line 117 is wrong. G4 is associated with active chromatin. This data does not suggest that it plays a positive role in transcription. It could even play a negative-feedback role.

Lines 158-159. With the resolution of ChIP binding of these factors directly to G4 versus direct recruitment to a bona fide G4-interacting protein cannot be distinguished. I would say “It is clear that these factors are tightly associated with G4, directly or indirectly.”

Similarly in the section “TFs selectively bind G4 structures”, the in vitro pulldown experiments used extracts to establish association of factors with G4. Therefore, the authors should say that these factors are associated with G4, or that bind directly or indirectly with G4.

Without functional studies, ascribing a universal positive role to G4 is not warranted. The authors need to qualify that they cannot exclude a role for G4 in moderating versus activating expression.

**Authors Response**

**Point-by-point responses to the reviewers’ comments:**

Reviewer 1

1) I though deem that experimental evidence on the TFs that interact at a given G4 is still required, to support the bioinformatics data that indicate a very peculiar and not so predictable feature of TF interaction at G4s, based on our current understanding of the system. There is no need to go single cell and the experiments to be performed are not very demanding: just measure by ChIP one or two G4 regions with the available anti-TF antibodies (those that have been tested in western blot would do nicely) by qPCR. This experiment would give a percentage of TFs (over the tested ones) that do actually interact at a given G4 region in a cell population.

*They key point being requested is to show explicit experimental data on TF binding at examples of particular G4s in chromatin derived from cells. We actually have experimental data on the G4 sites (from BG4 ChIP-seq) and TF binding data from ENCODE, therefore further ChIP-qPCR isn’t required to address this point. We have now exemplified the binding of several TFs at each of four examples of promoter G4s at different genes (STARD7, ZNF627, FBOX4 and KIF22). The examples show ChIP-seq data for the 22 TFs that were validated for G4 binding in our in vitro affinity enrichment western-blotting analysis (SP2, FUS, NRF1, NONO, GTF2F1, PRPF4, SP1, NELFE, SAFB, SRSF1, RBM15, SIN3A, MYC, BCLAF1, HNRNPLL, SETDB1, RBM14, HNRNPH1, YY1, ZHX1, TARDBP and TAF15). At all of these promoter G4s we observe binding for numerous of the selected TFs. Absolute or relative quantitation of the occupancy of each TF is not possible since the antibody/ChIP signal is non-linear and the efficiencies of different antibodies (many of which are polyclonal) to each TF vary. We have added a new supplementary figure (Additional File 1: Fig S8), which we now refer to in the result section (page 9):*

*“Notably, affinity-enrichment experiments from nuclear lysates cannot distinguish direct G4 binding from co-binding events, however, our findings are consistent with the recruitment of numerous TFs to G4 structures in chromatin (Additional File 1: Fig. S8).”*

*Fig. S8. TFs are recruited to G4s in chromatin.*

*ChIP-seq density from K562 cells for BG4 ChIP-seq (blue, three representative replicates) and ENCODE ChIP-seq (black and grey) for TFs at four different promoters. The 22 TFs (one representative replicate each) are shown that have been validated to bind DNA G4 oligonucleotides from K562 nuclear lysate. Potential G4 sites [10] on the forward (+) and reverse (-) strand (ochre) as well as BG4 high confidence peak sites (blue shade) are indicated. (SP2 (ENCFF153ANI), FUS (ENCFF958RAN), NRF1 (ENCFF674LRE), NONO (ENCFF984PYA), GTF2F1 (ENCFF698GEX), PRPF4 (ENCFF103MEZ), SP1 (ENCFF593LCA), NELFE (ENCFF000YUF), SAFB (ENCFF908KFH), SRSF1 (ENCFF975FFH), RBM15 (ENCFF402MOS), SIN3A (ENCFF154ZWB), MYC (ENCFF000YKV), BCLAF1(ENCFF144FIS), HNRNPLL(ENCFF768SVS), SETDB1 (ENCFF247BJO), RBM14 (ENCFF957SXO), HNRNPH1 (ENCFF594IKC), , YY1 (ENCFF193QAR), ZHX1 (ENCFF899GZH), TARDBP (ENCFF216EBS) and TAF15 (ENCFF818HIH)).*

2) In Fig S3e there are two Sp1 and two NRF1 signals. Is there a reason for this?

*Some of the TFs were investigated independently by different labs. As stated in the Methods section (see “Statistical analysis of genomic association”), ChIP-seq experiments targeting the same factor were treated independently. The fact that two independent experiments for SP1 and NRF1 show the direct proximity of TF and BG4 ChIP-seq peak centres in Fig S2e gives confidence in the robustness of our findings (Fig S3e does not exist).*

3) Fix this sentence “The enrichment level of SP2, NRF1, FUS, MYC, YY1 and ZHX1 and was comparable to their binding to consensus sequence controls (Fig. 2b and S7b),….” in the text.

*This has been corrected.*

Reviewer 2

1) Line 117 is wrong. G4 is associated with active chromatin. This data does not suggest that it plays a positive role in transcription. It could even play a negative-feedback role.

*We have clarified our previous statement in the results section (page 6):*

*“Endogenous G4s were substantially devoid of both transcriptional repressors (e.g. CBX8, ZNF318, EZH2 and PHB2) and repressive histone marks (e.g. H3K27me3, H3K9me3) (Fig. 1a and Fig. S2) which is consistent with previous observations that endogenous promoter G4s are linked to high transcription levels [16,17].”*

2) Lines 158-159. With the resolution of ChIP binding of these factors directly to G4 versus direct recruitment to a bona fide G4-interacting protein cannot be distinguished. I would say "It is clear that these factors are tightly associated with G4, directly or indirectly."

*We have amended our previous statement (page 8):*

*“Our findings support that these factors are tightly associated with G4s and that some of these proteins can be recruited to chromatin via DNA G4 structures.”*

3) Similarly in the section "TFs selectively bind G4 structures", the in vitro pulldown experiments used extracts to establish association of factors with G4. Therefore, the authors should say that these factors are associated with G4, or that bind directly or indirectly with G4.

*We have added the following clarification to the result section (page 9):*

*“Notably, affinity-enrichment experiments from nuclear lysates cannot distinguish direct G4 binding from co-binding events, however, our findings are consistent with the recruitment of numerous TFs to G4 structures in chromatin (Additional File 1: Fig. S8).”*

4) Without functional studies, ascribing a universal positive role to G4 is not warranted. The authors need to qualify that they cannot exclude a role for G4 in moderating versus activating expression.

*We have added this consideration to the discussion (page 14):*

*“Further functional studies are required to ascertain whether there is a universally positive role of promoter G4s in transcription and to explore the details of mechanisms that maintain the endogenous G4 landscape in chromatin[55].”*
